# Assessing the Clinical Treatment Dynamics of Antiplatelet Therapy Following Acute Coronary Syndrome and Percutaneous Coronary Intervention in the US

**DOI:** 10.1001/jamanetworkopen.2023.8585

**Published:** 2023-04-17

**Authors:** Yehua Wang, Larisa H. Cavallari, Joshua D. Brown, Cameron D. Thomas, Almut G. Winterstein

**Affiliations:** 1 Department of Pharmaceutical Outcomes and Policy, College of Pharmacy, University of Florida, Gainesville; 2 Center for Drug Evaluation and Safety, University of Florida, Gainesville; 3 Department of Pharmacotherapy and Translational Research and Center for Pharmacogenomics and Precision Medicine, College of Pharmacy, University of Florida, Gainesville

## Abstract

**Importance:**

A platelet ADP P2Y12 receptor (P2Y12) inhibitor plus aspirin is standard therapy for patients undergoing percutaneous coronary intervention (PCI) for acute coronary syndrome (ACS). Compared with clopidogrel, prasugrel and ticagrelor are associated with superior antiatherothrombotic effects but increased bleeding risk; with recent guideline updates, it is important to describe current treatment patterns and the role of bleeding risk in treatment choice.

**Objective:**

To describe secular trends and determinants of initial P2Y12 inhibitor choice and switching, including deescalation (switch from prasugrel or ticagrelor to clopidogrel).

**Design, Setting, and Participants:**

This retrospective cohort study used MarketScan Commercial Claims Data from 2010 to 2019 for patients aged 18 years or older who underwent PCI for ACS, had no P2Y12 inhibitor use in the past year, and filled a P2Y12 inhibitor prescription within 30 days after PCI hospitalization discharge. Data were analyzed from February to May 2022.

**Exposures:**

Clopidogrel, prasugrel, and ticagrelor, with determinants including bleeding risk measured using Academic Research Consortium for High Bleeding Risk criteria, sociodemographic characteristics, P2Y12 inhibitor copays, and bleeding events during follow-up.

**Main Outcomes and Measures:**

The prevalence of each P2Y12 inhibitor among patients who initiated the drugs and the prevalence of switching within 12 months after PCI were evaluated. The association between baseline bleeding risk and bleeding manifestations during follow-up and initial treatment and deescalation were calculated using multivariable logistic and Cox proportional hazards regression models.

**Results:**

Between 2010 and 2019, 62 423 patients were identified who initiated P2Y12 inhibitors (females, 22.4%; males, 77.6%; mean [SD] age, 54.32 [7.13] years). The prevalence of clopidogrel as initial therapy decreased from 77.5% in 2010 to 29.6% in 2019, while initial use of prasugrel or ticagrelor increased from 22.5% to 60.4%. Within 1 year after PCI, 11.0% of patients switched therapy, mostly for deescalation. Deescalation prevalence increased from 1.8% in 2010 to 12.6% in 2018. Between 2016 and 2018, 8588 of 22 886 (37.5%) patients had major baseline bleeding risk, which decreased the selection of prasugrel or ticagrelor as initial therapy (adjusted odds ratio, 0.78; 95% CI, 0.74-0.84). Among 11 285 patients who initiated prasugrel or ticagrelor, major bleeding risk at baseline (adjusted hazard ratio, 1.11; 95% CI, 1.00-1.23) and the occurrence of bleeding during follow-up (adjusted hazard ratio, 4.42; 95% CI, 3.62-5.93) were associated with deescalation.

**Conclusions and Relevance:**

A strong shift in preference for prasugrel and ticagrelor as initial therapy following PCI for ACS was observed. Deescalation increased over the study period. Major bleeding risk at baseline was moderately associated with initial treatment choice but had a limited association with deescalation. The increasing use of more potent P2Y12 inhibitors emphasizes opportunities to enhance preemptive patient-centered treatment strategies to maintain optimal antiplatelet activity while reducing bleeding risk during the subacute period following PCI for ACS.

## Key Points

**Question:**

What are the treatment dynamics of platelet ADP P2Y12 receptor (P2Y12) inhibitors and factors influencing initial choice and switching following acute coronary syndrome and percutaneous coronary intervention?

**Findings:**

In this cohort study of 62 423 patients between 2010 and 2019, switching from ticagrelor, which was the primary initial P2Y12 inhibitor prescribed in 2019, or prasugrel to clopidogrel (deescalation) increased across the study period, but remained infrequent (12.6%). Baseline bleeding risk played a role in initial P2Y12 inhibitor selection but had a limited association with switching, while manifestation of bleeding was significantly associated with deescalation.

**Meaning:**

The study’s findings suggest that ticagrelor has emerged as the most commonly prescribed P2Y12 inhibitor, there has been an increase in deescalation to clopidogrel during maintenance, and high bleeding risk at baseline can guide initial treatment selection, highlighting the need for preemptive patient-centered P2Y12 inhibitor prescribing to optimize antiplatelet activity and minimize bleeding risk.

## Teaser

This cohort study uses national claims data to describe platelet ADP P2Y12 receptor inhibitor choice and switching among patients who underwent percutaneous coronary intervention for acute coronary syndrome.

## Introduction

Dual antiplatelet therapy (DAPT) with a platelet ADP P2Y12 receptor (P2Y12) inhibitor plus aspirin is critical to prevent major adverse cardiovascular events (MACE) following acute coronary syndrome (ACS) and percutaneous coronary intervention (PCI).^1^ For patients with ACS, the guidelines recommend DAPT for at least 1 year after PCI.^1^ Clopidogrel, prasugrel, and ticagrelor are the 3 most prescribed oral P2Y12 inhibitors in the US.

In early clinical trials, prasugrel and ticagrelor were superior to clopidogrel in reducing MACE.^2,3^ Accordingly, the 2016 American Heart Association/American College of Cardiology guidelines recommend a preference for prasugrel and ticagrelor over clopidogrel for patients with ACS after PCI.^1^ Importantly, the genotype for the *CYP2C19* enzyme, which metabolizes clopidogrel (a prodrug) to its active form, contributes to reduced clopidogrel effectiveness and was not considered in early clinical trials.^4,5^ Approximately one-third of patients have a *CYP2C19* loss-of-function allele,^6^ which results in lower formation of the active clopidogrel metabolite, higher on-treatment platelet reactivity, and higher risk for MACE after PCI with clopidogrel treatment.^4,7,8^

Prasugrel and ticagrelor are more potent antiplatelet agents than clopidogrel,^9^ and the *CYP2C19* genotype has no effect on their efficacy.^4,7^ However, their use is accompanied by a higher risk of non–coronary artery bypass graft–related bleeding.^2,3,10^ Given that the risk of recurrent ischemic events is most pronounced early after PCI, while the bleeding risk increases as the treatment duration extends,^8,11^ initiating treatment with ticagrelor or prasugrel and then deescalating to clopidogrel after the early, high-risk period has been promoted as a means to maximize anti-ischemic benefits and reduce bleeding risk.^12,13^ Since 2016, trials have tested switching regimens at fixed times (eg, 30 days after DAPT initiation)^14,15^ or based on platelet function tests^16^ or *CYP2C19* genotype^17^ to achieve an improved balance between ischemic event prevention and bleeding risk reduction, with mixed results.^18^ Given that guided deescalation methods, at a minimum, did not increase ischemic event risk in clinical trials^16,17^ and have the potential to reduce bleeding risk, 2021 European Society of Cardiology (ESC) guidelines and a 2019 international expert consensus on P2Y12 inhibitor switching recommend consideration of deescalation for select patients, such as those with high bleeding risk.^13,19,20^

Given the emerging evidence supporting more tailored and patient-centered approaches, it is important to describe recent trends in P2Y12 inhibitor prescribing among patients with ACS. The most recent published data on P2Y12 inhibitor use in clinical practice end in 2016, which precedes the time when evidence supporting deescalation approaches started to emerge.^21,22,23^ In addition, there are minimal data on the outcomes of the 2016 guidelines stating preference for prasugrel and ticagrelor associated with P2Y12 inhibitor use. Furthermore, with guidelines emphasizing guided deescalation approaches to mitigate bleeding risk,^19,24^ it is important to identify patient-specific determinants of initial P2Y12 inhibitor selection and P2Y12 inhibitor deescalation.

In this study, we describe secular trends in initial P2Y12 inhibitor choice, changes in P2Y12 inhibitor therapy, and deescalation patterns in the 12 months following PCI. We also assess the factors associated with both initial P2Y12 inhibitor selection and deescalation, including bleeding risk and bleeding events.

## Methods

### Data Source

This retrospective cohort study was conducted using data from the MarketScan Commercial Claims Database between 2010 and 2019. The data include inpatient and outpatient medical encounters and pharmacy dispensing claims for a large national sample of the privately insured US population. The encrypted patient identifiers in the database allow for longitudinal follow-up. The data were certified as deidentified, and the University of Florida institutional review board therefore considered the study exempt from review and informed consent. The study followed the Strengthening the Reporting of Observational Studies in Epidemiology (STROBE) reporting guideline.^25^

### Study Cohorts

We included all patients aged 18 years or older who underwent PCI and had a primary diagnosis code of ACS on their PCI hospital encounter. We specifically focused on patients diagnosed with myocardial infarction. Patients were required to have 365 days of continuous health and prescription drug plan enrollment before their PCI hospitalization discharge to allow measurement of baseline characteristics. We excluded patients with any P2Y12 inhibitor (ie, clopidogrel, prasugrel, ticagrelor) prescription fill in the prior year. Patients were required to have their first P2Y12 inhibitor prescription filled within 30 days after PCI hospitalization discharge. Patients who filled prescriptions for more than 1 P2Y12 inhibitor on the same day were excluded.

The continuous enrollment requirement after discharge varied according to the analysis. To assess initial P2Y12 inhibitor choice and treatment persistence, patients were required to have at least 30 days of continuous enrollment after discharge. For assessment of P2Y12 inhibitor switching, patients were required to have 365 days of continuous enrollment after their first P2Y12 inhibitor fill. Patients could contribute multiple PCI and P2Y12 initiation episodes to the analysis as long as all eligibility criteria were met. For all secular trend analyses, episodes were anchored to 1 calendar year based on the date of PCI hospitalization discharge. The cohort entry criteria, look-back period, and follow-up are summarized in eFigure 1 in Supplement 1.

Percutaneous coronary intervention was identified using *International Classification of Diseases, Ninth Revision, Clinical Modification* (*ICD-9-CM*) procedure codes, the *International Statistical Classification of Diseases, Tenth Revision* (*ICD-10*), *Procedure Coding System*, and *Current Procedural Terminology* codes on inpatient encounters. Acute coronary syndrome diagnoses were identified using *ICD-9-CM* diagnostic codes and *ICD-10-CM* codes. National Drug Codes on outpatient pharmacy dispensing claims were used to identify P2Y12 inhibitor use (eTable 1 in Supplement 1).

### Outcomes

We assessed the initial choice of P2Y12 inhibitor after the PCI discharge, the switching rate, direction, and treatment persistence during 1 year of maintenance. The initial choice of P2Y12 inhibitor was defined as the first P2Y12 inhibitor prescription filled within 30 days after PCI hospitalization discharge. Annual prevalence was calculated as the proportion of patients who initiated each of the 3 study drugs out of all eligible P2Y12 initiators.

A switching event was defined as a prescription fill for a different P2Y12 inhibitor following the initial fill within 365 days. We categorized switching types as escalation (from clopidogrel to prasugrel or ticagrelor), deescalation (from prasugrel or ticagrelor to clopidogrel), and change (prasugrel to ticagrelor or vice versa). Annual switching prevalences were then measured as the proportion of patients who switched within each of these categories among all eligible participants starting a specific P2Y12 inhibitor within 1 year of follow-up. To ensure a 1-year follow-up for this analysis, the latest date for cohort entry was December 2018.

We calculated the time to treatment deescalation as the time from the initial prasugrel or ticagrelor dispensing to the first clopidogrel dispensing. Patients were required to be persistent with treatment to be considered for the time-to-deescalation analysis. We followed up these patients for a maximum of 1 year.

Treatment persistence was defined as the time from the initial P2Y12 inhibitor dispensing to treatment discontinuation. Treatment discontinuation was defined as a more than 30-day gap between the last day of supply from the prior dispensing and the fill date, if any, of the next dispensing. We estimated treatment persistence accounting or not accounting for switching (ie, persistence with P2Y12 inhibitor treatment vs with the initial P2Y12 inhibitor choice) and followed patients for a maximum of 1 year. Patients with shorter enrollment were followed until their disenrollment to account for deaths.

### Exposure and Covariates

To evaluate determinants of initial P2Y12 inhibitor choice and deescalation, considering the emerging evidence on deescalation and the changing P2Y12 inhibitor use pattern, we only included patients discharged since 2016. We chose 2016 based on important evidence related to deescalation published since 2016 that resulted in guidelines (eg, ESC guidelines^13^) and expert consensus^12,19^ on deescalation updated after 2016 to reflect the most recent trend.

We first assessed baseline (ie, 12 months before the first P2Y12 inhibitor fill date) characteristics for their association with the initial choice of P2Y12 inhibitors. We measured baseline bleeding risk using the Academic Research Consortium for High Bleeding Risk (ARC-HBR) criteria.^26^ The ARC-HBR has shown good validity in predicting bleeding events among patients who undergo PCI.^27,28^ Bleeding risk factors included recent major surgery (eg, joint replacement 30 days before PCI admission); recent anticoagulation drug use (3 months before and 30 days after PCI hospitalization discharge); and diagnoses of cancer, liver cirrhosis, thrombocytopenia, severe chronic kidney disease, stroke, anemia, or arteriovenous malformations in the past year (eTable 2 in Supplement 1). In addition, major bleeding risk was analyzed as a composite binary variable per ACR-HBR criteria (ie, having at least 1 major bleeding risk). Details on ARC-HBR items and measurement times and codes for each item are summarized in eTable 3 in Supplement 1. For other covariates, we included cardiovascular-related conditions and bleeding-related factors including diabetes, hypertension, dyslipidemia, heart failure, peripheral vascular disease (PVD), and atrial fibrillation in the model. The comorbidities were measured within the 12 months before the PCI hospitalization discharge date. Other assessed determinants included age at PCI hospitalization discharge, sex, patients’ health plan type, region, and the P2Y12 inhibitor copayment measured at the first fill date. The MarketScan database does not contain information on race and ethnicity; thus, these data were not collected or analyzed.

We also assessed determinants of deescalation during the 12-month follow-up, considering all baseline characteristics from the analysis of initial choice as well as any bleeding event during the 1-year follow-up, including nonmajor (eg, epistaxis) or major (eg, intracranial) bleeding.^29,30,31^ The diagnosis codes for bleeding events are summarized in eTable 4 in Supplement 1.

### Statistical Analysis

Drug-specific initiation and switching prevalence were plotted for each calendar year. To assess determinants of the initial choice of P2Y12 inhibitor, we used multivariable logistic regression considering all baseline covariates and comorbidities. To assess determinants of deescalation, we used a Cox proportional hazards regression considering all baseline covariates and bleeding events (nonmajor or major) during follow-up as a time-varying covariate with a 30-day risk (ie, the influence of a bleeding event as a determinant was considered for 30 days after occurrence). Major bleeding risk was analyzed as a composite per ACR-HBR criteria in 1 model (as a binary variable), and individual ACR-HBR components were analyzed individually in another model. Each model also considered the full set of patient baseline characteristics. We provide descriptive statistics of these variables across the study groups. Since the likelihood of missing values was low (<1%), we used complete case analyses.

Between February and May 2022, all analyses were performed using SAS, version 9.4 (SAS Institute Inc) statistical software, and visualization was performed using RStudio ggplot2 (RStudio). A 2-sided *P* < .05 was set as the threshold of significance.

## Results

### Initial P2Y12 Inhibitor Choice

**Figure 1.**
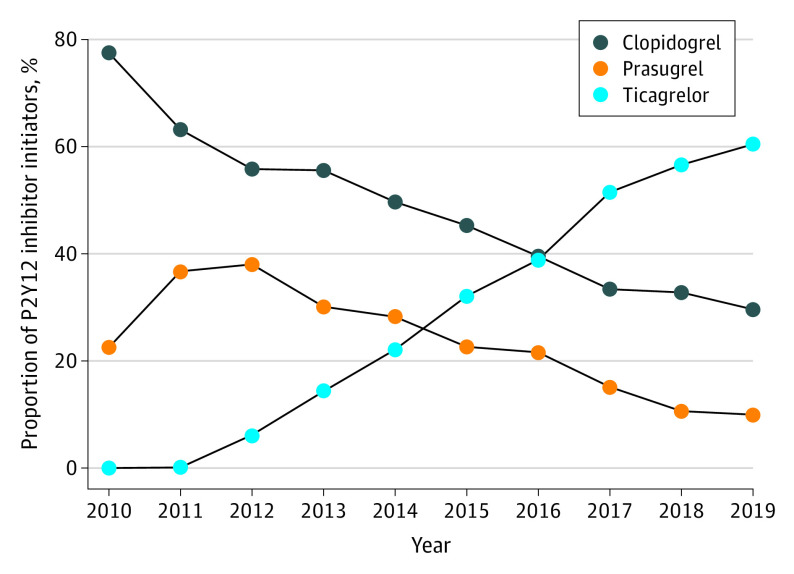
Initial Platelet ADP P2Y12 Receptor (P2Y12) Inhibitor Choice After Percutaneous Coronary Intervention, 2010-2019

Between 2010 and 2019, we identified 62 423 patients who met the eligibility criteria (mean [SD] age, 54.32 [7.13] years; females, 14 001 [22.4%]; males, 48 422 [77.6%]) (eFigure 2 in Supplement 1). Clopidogrel was the initial choice for 77.5% of patients who underwent PCI in 2010, and this proportion steadily decreased to 29.6% in 2019 (Figure 1). Following ticagrelor approval in 2011, there was a consistent increase in initiation prevalence (0.1% in 2011 to 60.4% in 2019) relative to the other 2 P2Y12 inhibitors. The proportion of patients filling prasugrel increased from 22.5% to 38.0% between 2010 and 2012 and decreased thereafter. Overall, the use of alternative therapy (prasugrel and ticagrelor) as the initial treatment option since PCI hospitalization discharge reached 70.4% in 2019.

### Determinants of Initial P2Y12 Inhibitor Choice

We identified 22 886 patients who initiated a P2Y12 inhibitor between 2016 and 2019; 7647 (33.4%) started on clopidogrel, 3421 (15.0%) started on prasugrel, and 11 818 (51.6%) started on ticagrelor. A total of 8588 patients (37.5%) met ARC-HBR major bleeding risk criteria at baseline (Table 1).

**Table 1.** Association Between Patient Baseline Characteristics and Initial Selection of Alternative Therapy vs Clopidogrel Among Patients With Acute Coronary Syndrome Treated With Percutaneous Coronary Intervention

Characteristic	No. (%)		aOR (95% CI)	
	Alternative therapy (n = 15 239)^a^	Clopidogrel (n = 7647)	Model 1^b^	Model 2^b^
Age, mean (SD), y	54.13 (7.15)	54.41 (7.19)	0.99 (0.98-0.99)^c^	0.99 (0.98-0.99)^c^
Sex				
Female	3294 (21.62)	1921 (25.12)	1.18 (1.11-1.26)^c^	1.17 (1.09-1.25)^c^
Male	11 945 (78.38)	5726 (74.88)	1 [Reference]	1 [Reference]
ACS type				
NSTEMI	4912 (32.23)	2919 (38.15)	1 [Reference]	1 [Reference]
STEMI	8096 (53.12)	3649 (47.69)	1.29 (1.21-1.37)^c^	1.30 (1.22-1.38)^c^
Other MI	2230 (14.64)	1083 (14.16)	1.29 (1.17-1.41)^c^	1.29 (1.18-1.42)^c^
Copay of the P2Y12 inhibitor, median (IQR), $	25 (0-50)	2.7 (0-5)	1.06 (1.06-1.06)^c^	1.07 (1.06-1.07)^c^
Health plan type				
PPO	8987 (58.98)	4507 (58.94)	1 [Reference]	1 [Reference]
Comprehensive	491 (3.22)	336 (4.39)	0.78 (0.67-0.92)^c^	0.78 (0.66-0.91)^c^
HMO	2024 (13.29)	994 (13.00)	0.91 (0.83-0.99)^c^	0.91 (0.83-0.99)^c^
Other	3737 (24.52)	1810 (23.67)	1.08 (1.00-1.15)^c^	1.07 (1.00-1.14)^c^
Region				
Northeast	2508 (16.46)	1251 (16.36)	1 [Reference]	1 [Reference]
North central	3432 (22.52)	1874 (24.51)	1.03 (0.94-1.14)	1.02 (0.93-1.13)
South	7574 (49.70)	3290 (43.02)	1.26 (1.15-1.37)^c^	1.23 (1.12-1.34)^c^
West	1679 (11.02)	1186 (15.51)	0.77 (0.69-0.86)^c^	0.76 (0.68-0.85)^c^
Hypertension	10 697 (70.19)	5508 (72.03)	0.91 (0.85-0.98)^c^	0.91 (0.85-0.93)^c^
Diabetes	5435 (35.66)	2833 (37.05)	1.05 (0.99-1.12)	0.99 (0.93-1.07)
Dyslipidemia	11 103 (72.86)	5441 (71.15)	1.11 (1.04-1.18)^c^	1.09 (1.02-1.16)^c^
Heart failure	1630 (10.70)	1045 (13.67)	0.88 (0.80-0.96)^c^	0.90 (0.83-0.99)^c^
PVD	541 (3.55)	383 (5.01)	0.83 (0.72-0.96)^c^	0.84 (0.74-0.99)^c^
Atrial fibrillation	585 (3.84)	568 (7.43)	0.57 (0.50-0.65)^c^	0.69 (0.60-0.79)^c^
ARC-HBR composite major bleeding risk	5356 (35.15)	3232 (42.26)	0.78 (0.74-0.84)^c^	NA
ARC-HBR individual major bleeding risk item^d^				
Recent major surgery	153 (1.00)	110 (1.44)	NA	0.88 (0.67-1.15)
Liver cirrhosis	280 (1.84)	251 (3.28)	NA	0.95 (0.76-1.17)
Anemia	831 (5.45)	676 (8.84)	NA	0.80 (0.71-0.90)^c^
Severe CKD	3517 (23.08)	1929 (25.23)	NA	1.01 (0.94-1.10)
Recent major bleeding	889 (5.83)	664 (8.68)	NA	0.84 (0.75-0.95)^c^
Arteriovenous malformation	37 (0.24)	51 (0.67)	NA	0.51 (0.31-0.86)^c^
Stroke	497 (3.26)	484 (6.33)	NA	0.62 (0.54-0.72)^c^
Cancer	580 (3.81)	358 (4.68)	NA	0.98 (0.84-1.13)
Thrombocytopenia	207 (1.36)	203 (2.65)	NA	0.67 (0.54-0.84)^c^
Anticoagulation drug use	210 (1.38)	342 (4.47)	NA	0.24 (0.20-0.30)^c^

Abbreviations: ACS, acute coronary syndrome; aOR, adjusted odds ratio; ARC-HBR, Academic Research Consortium for High Bleeding Risk; CKD, chronic kidney disease; HMO, health maintenance organization; MI, myocardial infarction; NA, not applicable; NSTEMI, non–ST-segment elevation myocardial infarction; P2Y12, platelet ADP P2Y12 receptor; PPO, preferred provider organization; PVD, peripheral vascular disease; STEMI, ST-segment elevation myocardial infarction.

^a^ Alternative therapy means prasugrel or ticagrelor.

^b^ Model 1 only included ARC-HBR composite bleeding risk (binary), and model 2 included individual ARC-HBR major bleeding risk items.

^c^ Significant statistical results at 2-sided *P* < .05.

^d^ The ARC-HBR criteria definitions and measurement windows are defined in eTable 2 in Supplement 1.

After adjusting for patients’ baseline characteristics and previous conditions, having an ARC-HBR composite major bleeding risk (ie, at least 1 ARC-HBR major bleeding risk factor) at baseline reduced the probability of selecting alternative agents as initial therapy (adjusted odds ratio [aOR], 0.78; 95% CI, 0.74-0.84). Of the individual components of the bleeding risk score, anemia (aOR, 0.80; 95% CI, 0.71-0.90), recent major bleeding event (aOR, 0.84; 95% CI, 0.75-0.95), stroke (aOR, 0.62; 95% CI, 0.54-0.72), arteriovenous malformation (aOR, 0.51; 95% CI, 0.31-0.86), thrombocytopenia (aOR, 0.67; 95% CI, 0.54-0.84), and anticoagulation drug use (aOR, 0.24; 95% CI, 0.20-0.30) were negatively associated with the use of prasugrel and ticagrelor. Besides the ARC-HBR major bleeding risk items, having hypertension (aOR, 0.91; 95% CI, 0.85-0.93), heart failure (aOR, 0.90; 95% CI, 0.83-0.99), PVD (aOR, 0.84; 95% CI, 0.74-0.99), or atrial fibrillation (aOR, 0.69; 95% CI, 0.60-0.79) was negatively associated with the use of alternative therapy, and having dyslipidemia (aOR, 1.09; 95% CI, 0.93-1.13) was positively associated with the use of alternative therapy.

The distribution of ARC-HBR composite major bleeding risk at baseline was similar between patients who initiated prasugrel or ticagrelor (aOR, 0.98; 95% CI, 0.90-1.07). However, when evaluating each risk factor individually, a diagnosis of anemia (aOR, 0.81; 95% CI, 0.67-0.98) and a history of stroke (aOR, 0.79; 95% CI, 0.62-1.00) were negatively associated with the use of prasugrel vs ticagrelor (eTable 5 in Supplement 1). Besides the ARC-HBR major bleeding risk items, having PVD was negatively associated with the use of prasugrel (aOR, 0.78; 95% CI, 0.63-0.98).

### P2Y12 Inhibitor Treatment Persistence

From 2010 to 2018, over the 1-year follow-up, the median time to clopidogrel discontinuation was 330 days (IQR, 150-364 days). For patients who initiated prasugrel and ticagrelor, the median number of days to discontinuation was 326 (IQR, 150-360 days) and 328 (IQR, 150-360 days), respectively.

### Switch Between P2Y12 Inhibitors

**Figure 2.**
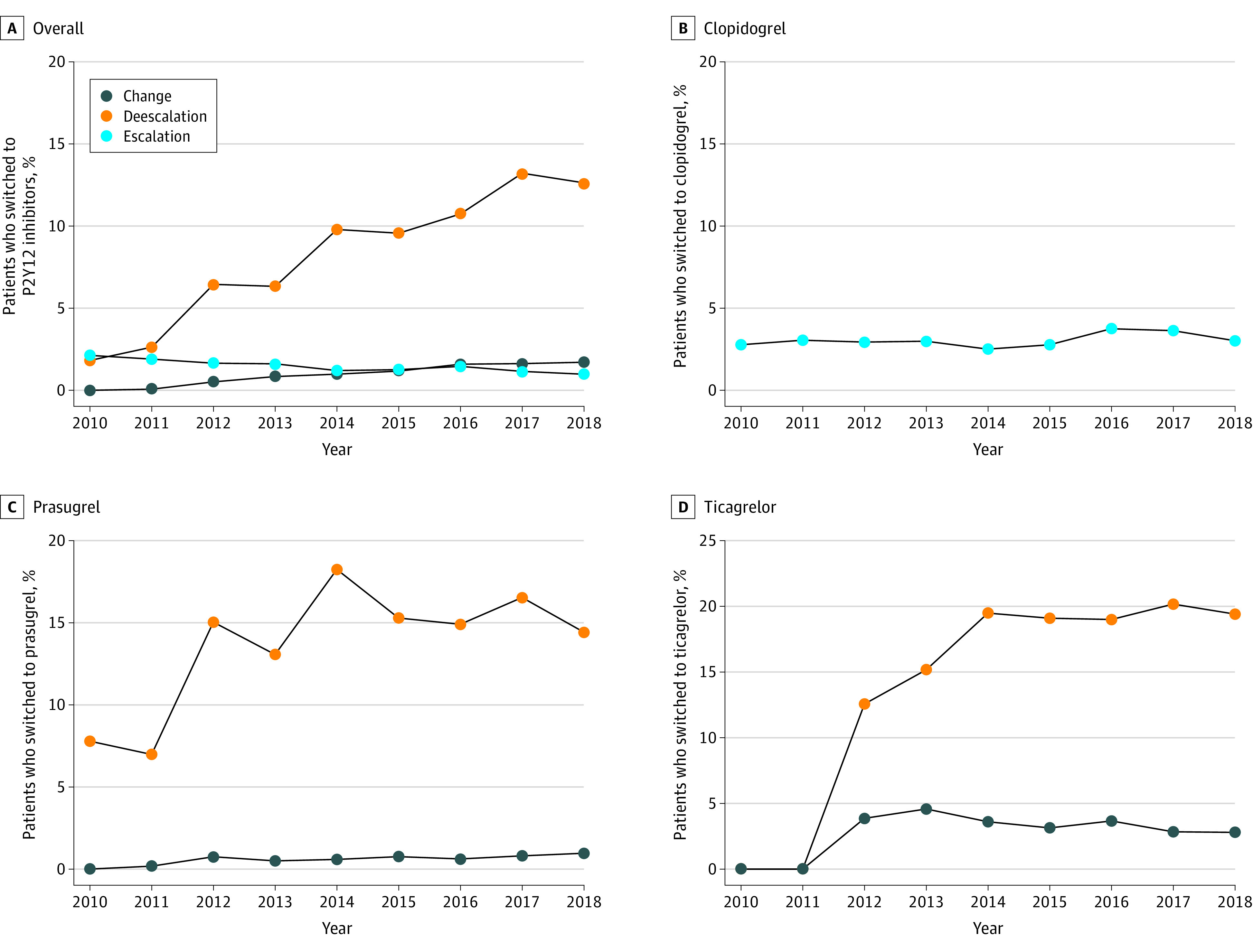
Annual Platelet ADP P2Y12 Receptor (P2Y12) Inhibitor Switching Rate and Direction, 2010-2018

From 2010 to 2018, there were 38 963 patients with 365 days of continuous enrollment after their initial P2Y12 inhibitor fill, including 4113 (11.0%) who switched therapy during the 1-year follow-up (Figure 2). The median time to switch was 55 days (IQR, 18-244 days) for clopidogrel, 313 days (IQR, 125-347 days) for prasugrel, and 284 days (IQR, 86-343 days) for ticagrelor. Deescalation from ticagrelor or prasugrel to clopidogrel was the most frequent switching type compared with escalation (ie, from clopidogrel to ticagrelor or prasugrel) and change between ticagrelor and prasugrel. The percentage of patients who deescalated increased from 1.8% in 2010 to 12.6% in 2018. Both escalation and change between alternative agents remained infrequent, ranging from 1.0% to 1.7%, throughout the study period.

### Determinants of Deescalation During Follow-up

We identified 11 285 patients who initiated prasugrel and ticagrelor between 2016 and 2018 (Table 2). Of these patients, 1898 (16.8%) deescalated to clopidogrel during follow-up, and 36.6% (694 of 1898) had a major bleeding risk at baseline. Of the 11 285 patients, 1145 (10.1%) had at least 1 bleeding event during follow-up (1029 [9.1%] had a major bleeding event). Patients with anticoagulation use (62 [29.5%]), arteriovenous malformation (7 [25.0%]), stroke (71 [20.3%]), anemia (120 [19.9%]), and a bleeding event (204 [17.5%]) had a higher deescalation rate than the whole population.

**Table 2.** Association Between Patient Baseline Characteristics and Bleeding Risk and Deescalation to Clopidogrel Among Patients Initiating Alternative Therapy^a^

Characteristic	No. (%)		aHR (95% CI)^b^	
	Deescalated (n = 1898)	Not deescalated (n = 9387)	Model 1	Model 2
Age, mean (SD), y	54.97 (6.82)	54.26 (7.27)	1.01 (1.01-1.02)^c^	1.01 (1.01-1.02)^c^
Sex				
Female	489 (25.9)	1977 (21.1)	0.77 (0.69-0.85)^c^	0.78 (0.70-0.86)^c^
Male	1409 (74.2)	7401 (78.8)	1 [Reference]	1 [Reference]
ACS type				
NSTEMI	615 (32.4)	2988 (31.8)	1 [Reference]	1 [Reference]
STEMI	1036 (54.6)	5198 (55.4)	0.97 (0.88-1.08)	0.97 (0.88-1.07)
Other MI	247 (13.0)	1201 (12.8)	0.99 (0.85-1.15)	0.99 (0.85-1.15)
Copay of the P2Y12 inhibitor, median (IQR), $	0 (0-40)	0 (0-20)	1.00 (1.00-1.00)^c^	1.00 (1.00-1.00)
Health plan type				
PPO	1134 (59.8)	5650 (60.2)	1 [Reference]	1 [Reference]
Comprehensive	56 (2.9)	318 (3.4)	0.82 (0.62-1.08)	0.83 (0.63-1.09)
HMO	275 (14.5)	1215 (12.9)	1.08 (0.95-1.23)	1.08 (0.95-1.23)
Other	433 (22.8)	2204 (23.5)	0.98 (0.88-1.10)	0.99 (0.88-1.10)
Region				
Northeast	287 (15.1)	1565 (16.7)	1 [Reference]	1 [Reference]
North central	488 (25.7)	2021 (21.5)	1.30 (1.13-1.51)^c^	1.32 (1.14-1.53)^c^
South	862 (45.4)	4741 (50.5)	0.99 (0.87-1.14)	1.00 (0.88-1.15)
West	253 (13.3)	1010 (10.8)	1.42 (1.20-1.68)^c^	1.44 (1.22-1.70)^c^
Hypertension	1326 (69.9)	6581 (70.1)	1.03 (0.93-1.14)	1.03 (0.93-1.14)
Diabetes	665 (35.0)	3334 (35.5)	0.98 (0.88-1.09)	1.00 (0.90-1.44)
Dyslipidemia	1332 (70.2)	6887 (73.4)	0.85 (0.76-0.94)^c^	0.85 (0.77-0.94)^c^
Heart failure	222 (11.7)	974 (10.4)	1.12 (0.97-1.29)	1.10 (0.96-1.27)
PVD	74 (3.9)	310 (3.3)	1.26 (0.90-1.45)	1.14 (0.90-1.44)
Atrial fibrillation	75 (3.9)	351 (3.7)	1.04 (0.83-1.32)	0.94 (0.73-1.19)
ARC-HBR composite major bleeding risk	694 (36.6)	3245 (34.6)	1.11 (1.00-1.23)^c^	NA
ARC-HBR individual major bleeding risk item^d^				
Recent major surgery	23 (1.2)	94 (1.0)	NA	1.10 (0.73-1.67)
Liver cirrhosis	35 (1.8)	168 (1.8)	NA	0.88 (0.60-1.29)
Anemia	120 (6.3)	482 (5.1)	NA	1.22 (1.01-1.49)^c^
Severe CKD	434 (22.9)	2125 (22.6)	NA	1.02 (0.90-1.15)
Recent major bleeding	109 (5.7)	554 (5.9)	NA	0.93 (0.76-1.14)
Arteriovenous malformation	7 (0.4)	21 (0.2)	NA	1.92 (0.83-4.45)
Stroke	71 (3.7)	278 (2.9)	NA	1.71 (0.92-2.50)
Cancer	83 (4.4)	338 (3.6)	NA	1.22 (0.98-1.52)
Thrombocytopenia	27 (1.4)	136 (1.4)	NA	0.86 (0.58-1.28)
Anticoagulation drug use	62 (3.3)	148 (1.6)	NA	1.81 (1.38-2.26)^c^

Abbreviations: ACS, acute coronary syndrome; aHR, adjusted hazard ratio; ARC-HBR, Academic Research Consortium for High Bleeding Risk; CKD, chronic kidney disease; HMO, health maintenance organization; MI, myocardial infarction; NA, not applicable; NSTEMI, non–ST-segment elevation myocardial infarction; P2Y12, platelet ADP P2Y12 receptor; PPO, preferred provider organization; PVD, peripheral vascular disease; STEMI, ST-segment elevation myocardial infarction.

^a^ Alternative therapy means prasugrel or ticagrelor.

^b^ Model 1 only included ARC-HBR composite bleeding risk (binary), and model 2 included individual ARC-HBR major bleeding risk items.

^c^ Significant statistical results at 2-sided *P* < .05.

^d^ The ARC-HBR criteria definition and measurement window are defined in eTable 2 in Supplement 1.

Having a major bleeding risk at baseline was associated with an 11.0% increase in the hazard of deescalation (adjusted hazard ratio [aHR], 1.11; 95% CI, 1.00-1.23), while manifestation of bleeding during follow-up showed a stronger association (aHR, 4.42; 95% CI, 3.62-5.93) (Table 3). Among patients with a bleeding event during follow-up, 98 (49.2%) were not considered as having a major bleeding risk at baseline. The association between baseline bleeding risk and deescalation remained even if bleeding events during follow-up were not considered in the regression model. Other bleeding risk factors that were associated with deescalation included anemia (aHR, 1.22; 95% CI, 1.01-1.49) and anticoagulation use (aHR, 1.81; 95% CI, 1.38-2.26) at baseline (Table 2). Other associations between baseline patient characteristics and bleeding risk and deescalation to clopidogrel are shown in eTable 6 in Supplement 1.

**Table 3.** Association Between Baseline Characteristics and Bleeding Events During Follow-up and Time to Deescalation Among Patients Initiating Prasugrel or Ticagrelor

Characteristic	aHR (95% CI)		
	Alternative therapy^a^	Prasugrel	Ticagrelor
Age, y	1.01 (1.00-1.02)^b^	1.00 (0.98-1.02)	1.02 (1.01-1.03)^b^
Female	0.76 (0.67-0.85)^b^	0.91 (0.64-1.29)	0.69 (0.59-0.80)^b^
ACS type			
NSTEMI	1 [Reference]	1 [Reference]	1 [Reference]
STEMI	1.06 (0.92-1.21)	0.94 (0.62-1.43)	0.94 (0.79-1.12)
Other MI	0.82 (0.64-1.04)	0.59 (0.29-1.19)	0.89 (0.68-1.16)
Copay of the P2Y12 inhibitor	1.00 (1.00-1.00)^b^	0.99 (0.99-1.00)	0.99 (0.99-1.00)
Health plan type			
PPO	1 [Reference]	1 [Reference]	1 [Reference]
Comprehensive	0.85 (0.64-1.13)	1.00 (0.60-1.64)	1.08 (0.51-2.68)
HMO	1.08 (0.93-1.26)	0.96 (0.62-1.47)	1.08 (0.75-1.55)
Other	0.91 (0.80-1.04)	0.88 (0.48-1.59)	1.14 (0.93-1.39)
Region			
Northeast	1 [Reference]	1 [Reference]	1 [Reference]
North central	1.24 (1.05-1.47)^b^	0.74 (0.31-1.73)	1.40 (1.13-1.75)^b^
South	1.18 (1.01-1.37)^b^	1.28 (0.87-1.89)	1.11 (0.90-1.36)
West	1.13 (0.93-1.39)	0.98 (0.67-1.44)	1.44 (1.11-1.88)^b^
Hypertension	0.96 (0.86-1.08)	1.06 (0.77-1.47)	1.05 (0.90-1.22)
Diabetes	1.16 (0.99-1.34)	0.82 (0.56-1.18)	1.08 (0.92-1.27)
Dyslipidemia	0.97 (0.86-1.09)	0.83 (0.59-1.15)	0.88 (0.75-1.02)
Heart failure	1.02 (0.86-1.21)	1.01 (0.62-1.64)	1.07 (0.87-1.32)
PVD	1.00 (0.75-1.32)	1.35 (0.62-2.95)	0.91 (0.62-1.32)
Atrial fibrillation	0.78 (0.58-1.05)	0.51 (0.19-1.34)	0.91 (0.63-1.31)
Any bleeding event during follow-up^c^	4.42 (3.62-5.93)^b^	7.00 (4.26-11.52)^b^	4.21 (3.21-5.50)^b^
ARC-HBR individual major bleeding risk item^d^			
Recent major surgery	1.17 (0.85-1.61)	1.49 (0.35-6.41)	0.93 (0.50-1.75)
Liver cirrhosis	1.03 (0.60-1.46)	1.72 (0.59-5.04)	1.00 (0.59-1.70)
Anemia	0.91 (0.71-1.17)	1.56 (0.84-2.89)	1.15 (0.86-1.54)
Severe CKD	0.90 (0.77-1.05)	1.09 (0.72-1.65)	0.96 (0.80-1.16)
Recent major bleeding	0.70 (0.55-0.88)	0.71 (0.38-1.30)	0.78 (0.57-1.07)
Arteriovenous malformation	1.61 (0.60-4.33)	2.28 (0.24-21.47)	2.25 (0.64-7.90)
Stroke	0.98 (0.74-1.30)	2.30 (1.20-4.43)^b^	1.08 (0.75-1.54)
Cancer	1.13 (0.87-1.48)	1.35 (0.58-3.17)	1.22 (0.89-1.68)
Thrombocytopenia	0.65 (0.38-1.12)	0.73 (0.17-3.20)	0.76 (0.41-1.40)
Anticoagulation drug use	1.04 (0.79-1.36)	2.58 (1.24-5.35)^b^	1.68 (1.18-2.38)^b^

Abbreviations: ACS, acute coronary syndrome; aHR, adjusted hazard ratio; ARC-HBR, Academic Research Consortium for High Bleeding Risk; CKD, chronic kidney disease; HMO, health maintenance organization; MI, myocardial infarction; NSTEMI, non–ST-segment elevation myocardial infarction; P2Y12, platelet ADP P2Y12 receptor; PPO, preferred provider organization; PVD, peripheral vascular disease; STEMI, ST-segment elevation myocardial infarction.

^a^ Alternative therapy means prasugrel or ticagrelor.

^b^ Significant statistical results at 2-sided *P* < .05.

^c^ Any bleeding event, including both major and nonmajor bleeding events, as assessed using a time-varying Cox proportional hazards regression model.

^d^ The ARC-HBR criteria definitions and measurement windows are defined in eTable 2 in Supplement 1.

## Discussion

This retrospective cohort study provides an updated assessment of use patterns and treatment dynamics of P2Y12 inhibitors among a national sample of patients with ACS who underwent PCI in the US. We leveraged the longitudinal structure of the claims database to describe not only the initial P2Y12 inhibitor choice but also drug use dynamics in the year following PCI. This study is also unique in its application of ARC-HBR criteria to evaluate the role of bleeding risk in initial treatment choice and P2Y12 inhibitor deescalation.

Our study found increased use of more potent P2Y12 inhibitors over time, which aligns with previous findings^21,23^ and with the 2016 American Heart Association/American College of Cardiology guideline recommendations.^1^ Interestingly, although prasugrel became available as a generic drug in 2017, ticagrelor was still more commonly prescribed at the end of the study period. According to the price listed in Lexicomp, the daily cost for the brand and generic formulations of prasugrel are $18.61 and $16.50, respectively, while for ticagrelor’s brand formulation, the daily cost is $17.02.^32^ Physicians may have been aware of this minimal cost difference; thus, generic availability of prasugrel may not have had a major impact on treatment selection.

Of note, while several systematic reviews published between 2017 and 2020 showed similar clinical benefits and bleeding risk of prasugrel and ticagrelor among patients with ACS who underwent PCI,^33,34,35^ studies published since 2019 found that prasugrel was superior in reducing myocardial infarction risk.^36,37,38,39^ Accordingly, in 2021, ESC guidelines gave a class IIa recommendation, preferring prasugrel over ticagrelor.^13,39^ Thus, the preference for ticagrelor over prasugrel identified in the current analysis might require further evaluation as newer drug use data become available.

Our analysis of determinants of initial treatment choice suggested that ARC-HBR criteria, as a composite risk factor, may play a role in the initial selection of P2Y12 inhibitor therapy. But major bleeding risk was not associated with the choice between prasugrel and ticagrelor, which is in line with current guideline recommendations.^13^ Consideration of the individual bleeding risk factors included in ARC-HBR criteria varied substantially in this regard. Anticoagulant use was most strongly associated with avoidance of prasugrel or ticagrelor, followed by several atherothrombotic risk factors. Regarding selection of prasugrel vs ticagrelor, similar to a previous study,^23^ we found that history of stroke was associated with the avoidance of prasugrel, which is consistent with previous findings and the prasugrel package insert.^40^ Different from previous findings in patients from 2011 to 2013, right after ticagrelor’s approval in the US, we did not find an association with overall bleeding risk or most bleeding risk components. The strong increase in prescribers’ preference of ticagrelor in more recent years might explain this finding. Broader clinical adoption of bleeding risk assessments in P2Y12 inhibitor selection may aid in individualizing drug selection.

Overall, switching between P2Y12 inhibitors remained fairly uncommon during the study period, although deescalation showed a steady increase to approximately 12% of patients by the end of the study period, which is similar to previous findings.^41,42,43,44^ Although the deescalation rate of ticagrelor has increased to approximately 20%, this increase has been observed since ticagrelor’s approval, before the publication of a deescalation consensus^12^ and recent clinical trials exploring deescalation based on clinical and genetic parameters.^16,17^ Thus, trial findings appear to have had a limited impact so far on switching in clinical practice.

We focused on baseline bleeding risk and bleeding events as potential promoters of P2Y12 inhibitor deescalation. For patients who initiated prasugrel or ticagrelor, having an ARC-HBR major bleeding risk factor was associated with future deescalation; however, most individual bleeding risk factors showed no association except for anticoagulant use and anemia, suggesting that assessment of bleeding risk is more prominent in initial P2Y12 inhibitor selection vs deescalation. In contrast, the occurrence of a bleeding event was significantly associated with deescalation during follow-up (aHR, 4.42; 95% CI 3.62-5.93). Of note, nearly 50% of patients who had a bleeding event and deescalated therapy were not considered as being at major bleeding risk at baseline according to a well-validated bleeding risk score. This relatively high percentage suggests a need for a validated, preemptive risk assessment tool that can estimate bleeding risk before P2Y12 inhibitor selection and then periodic reassessment to determine whether deescalation may be warranted to individualize both P2Y12 inhibitor selection and deescalation approaches to optimize DAPT after PCI.

### Limitations

This study has several limitations. First, we operationalized the ARC-HBR major bleeding risk criteria from claims data, which do not provide as much granularity as electronic health records to ascertain the severity of conditions that increase bleeding risk (eg, anemia). Specifically, while the ARC-HBR criteria use specific platelet count cutoffs, we had to rely on thrombocytopenia diagnoses without such detail, which might have decreased the accuracy of our bleeding risk categorization and estimated associations with treatment selection and deescalation. Second, the measurement of bleeding events relied on health care encounters, and especially minor bleeds may have been missed. Thus, reported frequencies of bleeding events may be underestimated. Third, our study on deescalation focused on the switch from more potent P2Y12 inhibitors to clopidogrel. We recognize that there are other deescalation strategies in the current guidelines to reduce bleeding risk, such as aspirin discontinuation.^13,45^ Because aspirin can be obtained over the counter, which was not captured in the claims database, the current data source does not support analyses of aspirin discontinuation. These strategies will be assessed in other settings and studies in the future. Meanwhile, we tested other variables not included in the ARC-HBR criteria that might be associated with bleeding risk, including diabetes, hypertension, and dyslipidemia, but database limitations did not allow assessment of body mass index or race and ethnicity. Fourth, with regard to generalizability, our study patients were commercially insured individuals younger than 65 years, and our results may not extend to those who have other forms of insurance (eg, Medicaid, Medicare), public insurance, or no insurance.

## Conclusions

This cohort study found that preferences for clopidogrel from 2010 to 2019 shifted to a strong preference for more potent P2Y12 inhibitors and, especially, ticagrelor among privately insured patients with ACS after PCI. During the 1-year treatment period, switching between agents was uncommon, although the prevalence of deescalation increased to more than 12% by the end of the study period. Major bleeding risk at baseline was a factor moderately associated with initial P2Y12 inhibitor treatment choice but played a minor role in future deescalation. In contrast, the occurrence of bleeding events during follow-up was associated with deescalation, emphasizing opportunities to enhance preemptive patient-centered treatment strategies to better assess bleeding risk and tailor therapy accordingly.

## Notes

Supplement 1. **eFigure 1.** Study Design Diagrams **eFigure 2.** Flow Diagram for the Inclusion and Exclusion Criteria **eTable 1.** Codes for PCI, ACS, and P2Y12 Inhibitor Identification **eTable 2.** Summary of ARC-HBR Items and Measurement Time Windows **eTable 3.** Codes for Different ARC-HBR Item Measurements **eTable 4.** Codes for Bleeding Events **eTable 5.** Association Between Patient Characteristics at Baseline and Bleeding Risk and the Initial Prescription for Prasugrel vs Ticagrelor Among Patients With ACS Undergoing PCI **eTable 6.** Association Between Patient Characteristics at Baseline and Bleeding Risk and Deescalation to Clopidogrel Among Patients Started on Prasugrel or Ticagrelor

Supplement 2.
Data Sharing Statement
