# Supplementary material for: Assessing the Clinical Treatment Dynamics of Antiplatelet Therapy Following Acute Coronary Syndrome and Percutaneous Coronary Intervention in the US
Source: JAMA Netw Open. 2023 Apr 17;6(4):e238585. doi: 10.1001/jamanetworkopen.2023.8585 (PMC10111179; doi:10.1001/jamanetworkopen.2023.8585)
Supplement: Supplement 1. — eFigure 1. Study Design Diagrams eFigure 2. Flow Diagram for the Inclusion and Exclusion Criteria eTable 1. Codes for PCI, ACS, and P2Y12 Inhibitor Identification eTable 2. Summary of ARC-HBR Items and Measurement Time Windows eTable 3. Codes for Different ARC-HBR Item Measurements eTable 4. Codes for Bleeding Events eTable 5. Association Between Patient Characteristics at Baseline and Bleeding Risk and the Initial Prescription for Prasugrel vs Ticagrelor Among Patients With ACS Undergoing PCI eTable 6. Association Between Patient Characteristics at Baseline and Bleeding Risk and Deescalation to Clopidogrel Among Patients Started on Prasugrel or Ticagrelor [file jamanetwopen-e238585-s001.pdf]

Supplemental Online Content

Wang Y, Cavallari LH, Brown JD, Thomas CD, Winterstein AG. Assessing the clinical treatment dynamics of antiplatelet therapy following acute coronary syndrome and percutaneous coronary intervention in the US. *JAMA Netw Open*. 2023;6(4):e238585. doi:10.1001/jamanetworkopen.2023.8585

eFigure 1. Study Design Diagrams

eFigure 2. Flow Diagram for the Inclusion and Exclusion Criteria

eTable 1. Codes for PCI, ACS, and P2Y12 Inhibitor Identification

eTable 2. Summary of ARC-HBR Items and Measurement Time Windows

eTable 3. Codes for Different ARC-HBR Item Measurements

eTable 4. Codes for Bleeding Events

eTable 5. Association Between Patient Characteristics at Baseline and Bleeding Risk and the Initial Prescription for Prasugrel vs Ticagrelor Among Patients With ACS Undergoing PCI

eTable 6. Association Between Patient Characteristics at Baseline and Bleeding Risk and Deescalation to Clopidogrel Among Patients Started on Prasugrel or Ticagrelor

This supplemental material has been provided by the authors to give readers additional information about their work.

© 2023 Wang Y et al. *JAMA Network Open.*

eFigure 1. Study Design Diagrams

Supplementary Figure 1a. Study design diagram for the Initial $P2Y_{12}$ choice assessment

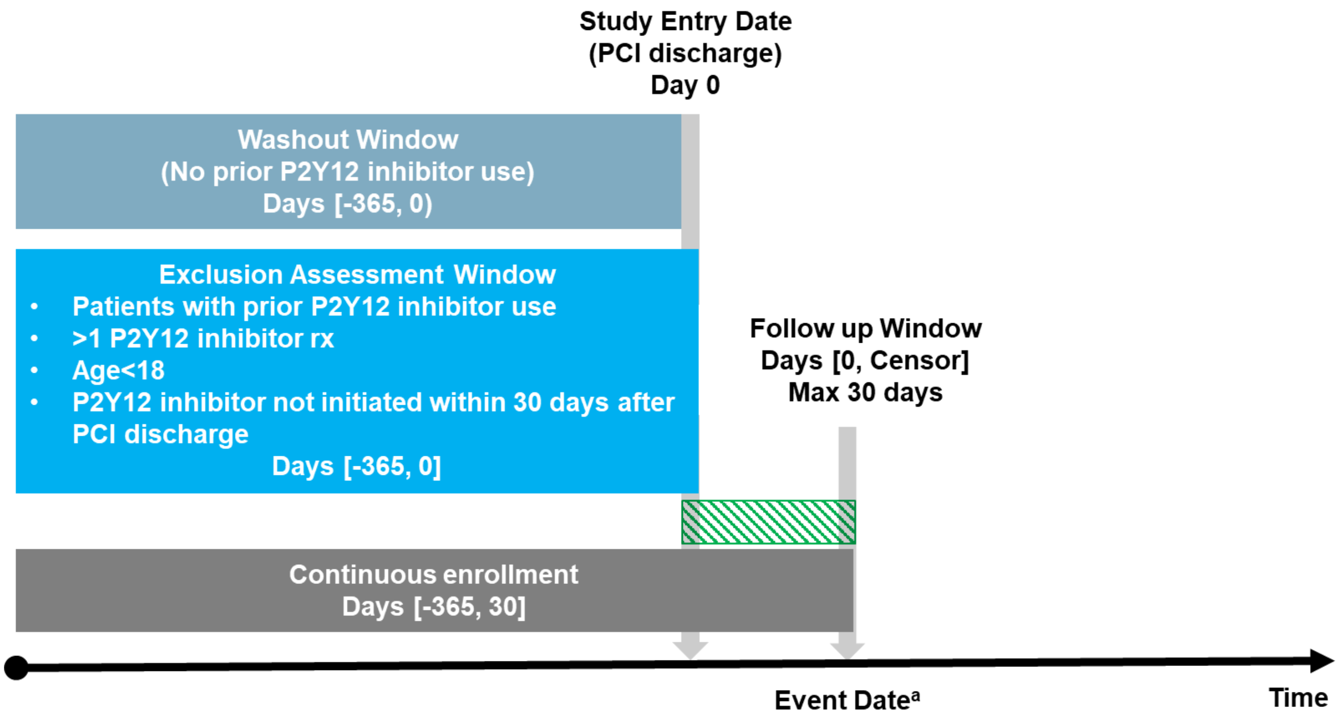

a. The date of the first P2Y12 inhibitor prescription after discharged from PCI

© 2023 Wang Y et al. *JAMA Network Open.*

Supplementary Figure 1b. Study design diagram for the $P2Y_{12}$ inhibitor switching assessment

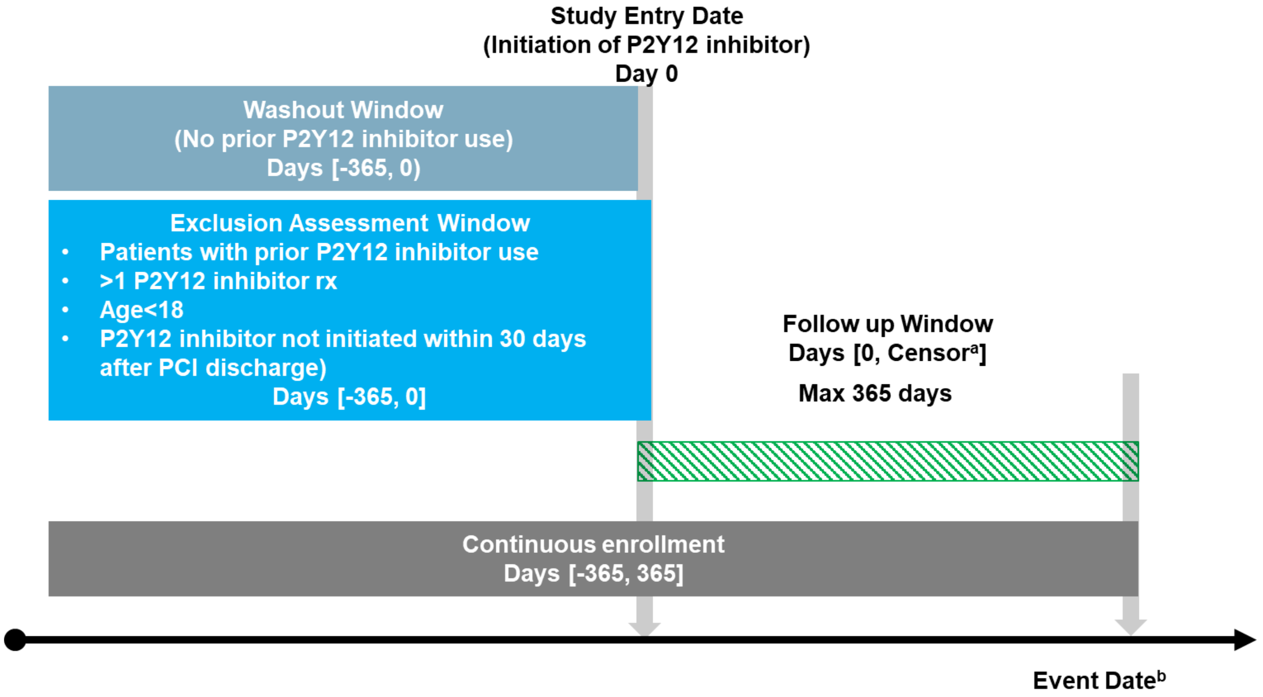

a. End of the prescription, event happening, death, etc.
b. The date when the first switching event happens

Time

© 2023 Wang Y et al. *JAMA Network Open.*

Supplementary Figure 1c. Study design diagram for the $P2Y_{12}$ inhibitor treatment persistence calculation

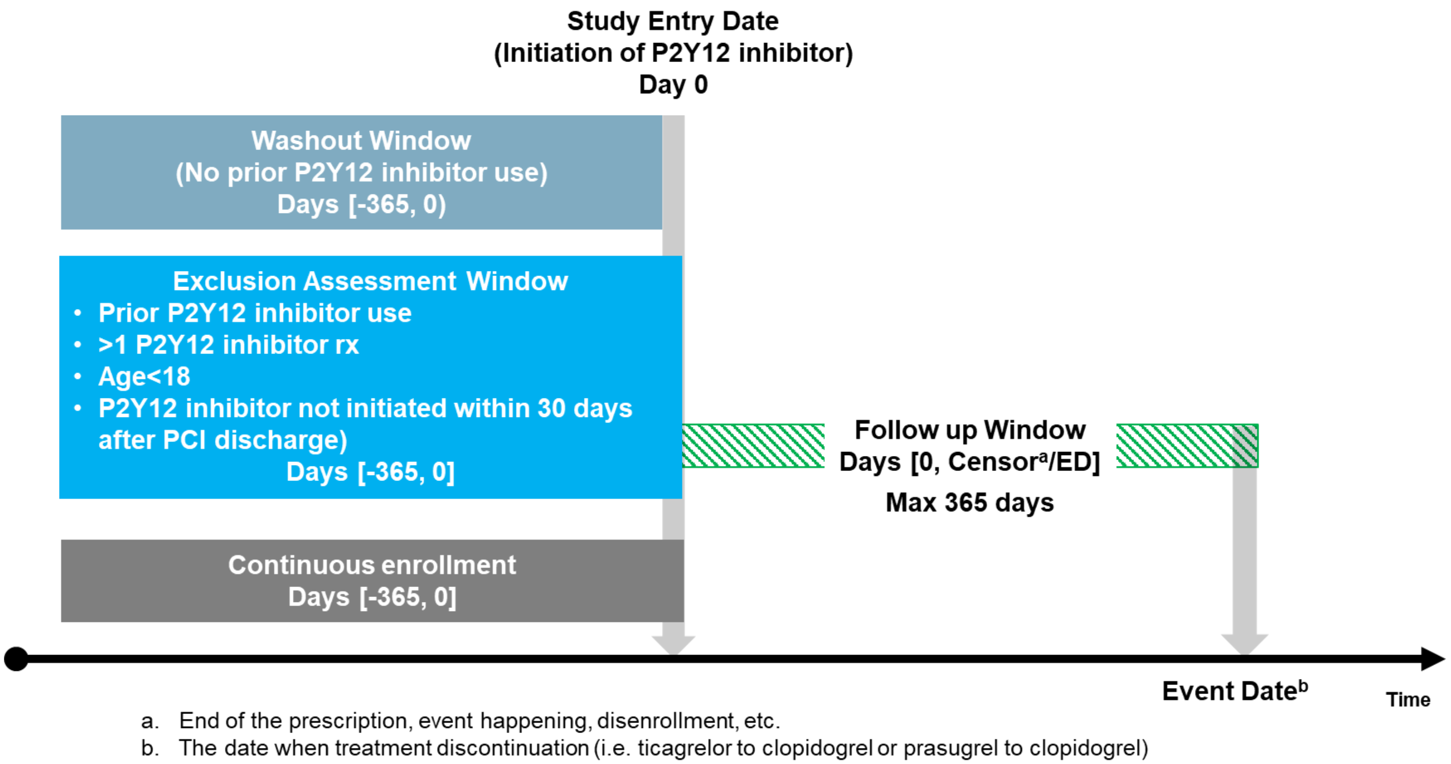

a. End of the prescription, event happening, disenrollment, etc.
b. The date when treatment discontinuation (i.e. ticagrelor to clopidogrel or prasugrel to clopidogrel)

© 2023 Wang Y et al. *JAMA Network Open.*

eFigure 2. Flow Diagram for the Inclusion and Exclusion Criteria

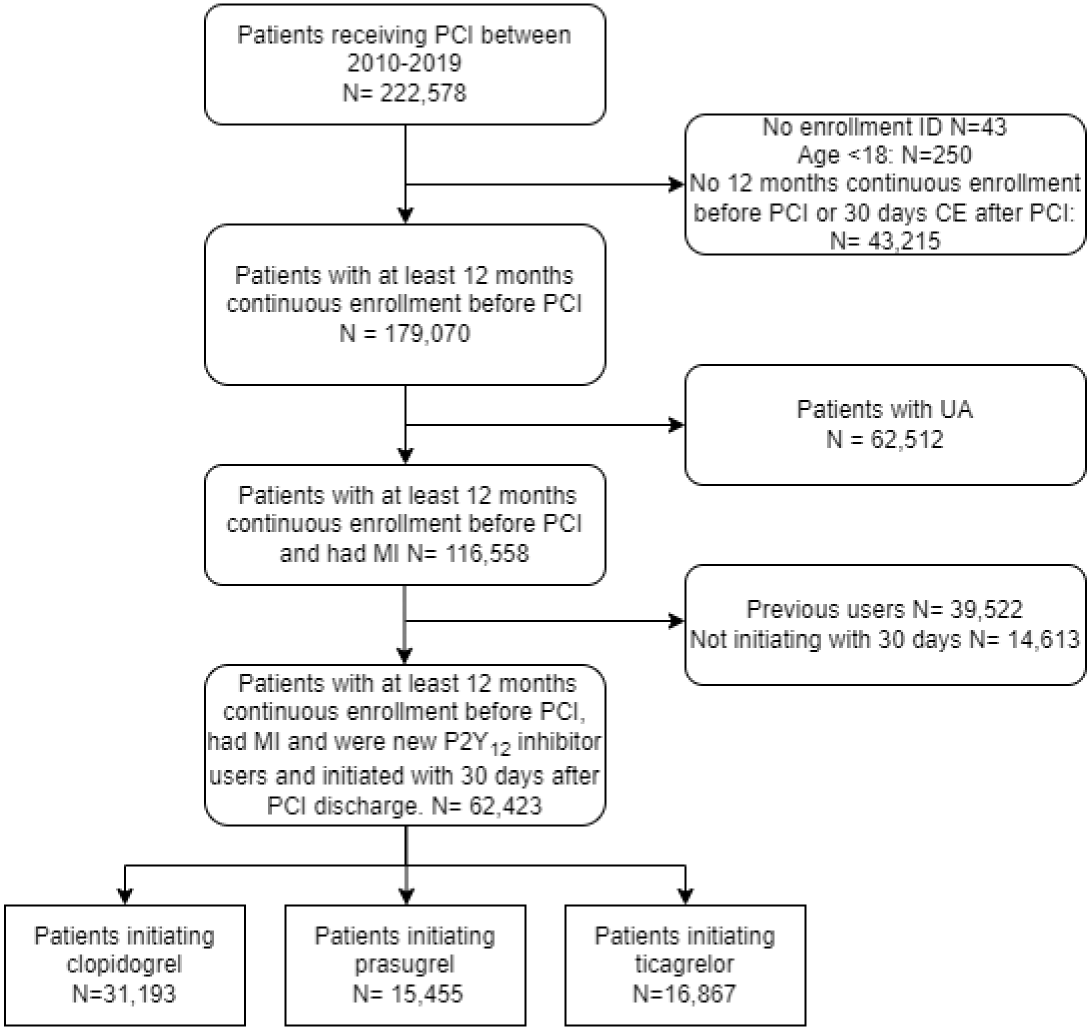

PCI, percutaneous coronary intervention; CE, continuous enrollment; UA, unstable angina; MI, myocardial infraction

© 2023 Wang Y et al. *JAMA Network Open.*

eTable 1. Codes for PCI, ACS, and P2Y12 Inhibitor Identification

Item	Code*
**PCI (ICD-9_PCS)**	066, 3606, 3607, 1755
**PCI (ICD-10-PCS)**	0270046, 027004Z, 0270056, 027005Z, 0270066, 027006Z, 0270076, 027007Z, 02700D6, 02700DZ, 02700E6, 02700EZ, 02700F6, 02700FZ, 02700G6, 02700GZ, 02700T6, 02700TZ, 02700Z6, 02700ZZ, 0270346, 027034Z, 0270356, 027035Z, 0270366, 027036Z, 0270376, 027037Z, 02703D6, 02703DZ, 02703E6, 02703EZ, 02703F6, 02703FZ, 02703G6, 02703GZ, 02703T6, 02703TZ, 02703Z6, 02703ZZ, 0270446, 027044Z, 0270456, 027045Z, 0270466, 027046Z, 0270476, 027047Z, 02704D6, 02704DZ, 02704E6, 02704EZ, 02704F6, 02704FZ, 02704G6, 02704GZ, 02704T6, 02704TZ, 02704Z6, 02704ZZ, 0271046, 027104Z, 0271056, 027105Z, 0271066, 027106Z, 0271076, 027107Z, 02710D6, 02710DZ, 02710E6, 02710EZ, 02710F6, 02710FZ, 02710G6, 02710GZ, 02710T6, 02710TZ, 02710Z6, 02710ZZ, 0271346, 027134Z, 0271356, 027135Z, 0271366, 027136Z, 0271376, 027137Z, 02713D6, 02713DZ, 02713E6, 02713EZ, 02713F6, 02713FZ, 02713G6, 02713GZ, 02713T6, 02713TZ, 02713Z6, 02713ZZ, 0271446, 027144Z, 0271456, 027145Z, 0271466, 027146Z, 0271476, 027147Z, 02714D6, 02714DZ, 02714E6, 02714EZ, 02714F6, 02714FZ, 02714G6, 02714GZ, 02714T6, 02714TZ, 02714Z6, 02714ZZ, 0272046, 027204Z, 0272056, 027205Z, 0272066, 027206Z, 0272076, 027207Z, 02720D6, 02720DZ, 02720E6, 02720EZ, 02720F6, 02720FZ, 02720G6, 02720GZ, 02720T6, 02720TZ, 02720Z6, 02720ZZ, 0272346, 027234Z, 0272356, 027235Z, 0272366, 027236Z, 0272376, 027237Z, 02723D6, 02723DZ, 02723E6, 02723EZ, 02723F6, 02723FZ, 02723G6, 02723GZ, 02723T6, 02723TZ, 02723Z6, 02723ZZ, 0272446, 027244Z, 0272456, 027245Z, 0272466, 027246Z, 0272476, 027247Z, 02724D6, 02724DZ, 02724E6, 02724EZ, 02724F6, 02724FZ, 02724G6, 02724GZ, 02724T6, 02724TZ, 02724Z6, 02724ZZ, 0273046, 027304Z, 0273056, 027305Z, 0273066, 027306Z, 0273076, 027307Z, 02730D6, 02730DZ, 02730E6, 02730EZ, 02730F6, 02730FZ, 02730G6, 02730GZ, 02730T6, 02730TZ, 02730Z6, 02730ZZ, 0273346, 027334Z, 0273356, 027335Z, 0273366, 027336Z, 0273376, 027337Z, 02733D6, 02733DZ, 02733E6, 02733EZ, 02733F6, 02733FZ, 02733G6, 02733GZ, 02733T6, 02733TZ, 02733Z6, 02733ZZ, 0273446, 027344Z, 0273456, 027345Z, 0273466, 027346Z, 0273476, 027347Z, 02734D6, 02734DZ, 02734E6, 02734EZ, 02734F6, 02734FZ, 02734G6, 02734GZ, 02734T6, 02734TZ, 02734Z6, 02734ZZ, 027L04Z, 027L0DZ, 027L0ZZ, 027L34Z, 027L3DZ, 027L3ZZ, 027L44Z, 027L4DZ, 027L4ZZ, 027W04Z, 027W0DZ, 027W0ZZ, 027W34Z, 027W3DZ, 027W3ZZ, 027W44Z, 027W4DZ, 027W4ZZ, 027X04Z, 027X0DZ, 027X0ZZ, 027X34Z, 027X3DZ, 027X3ZZ, 027X44Z, 027X4DZ, 027X4ZZ, 02C00Z6, 02C00ZZ, 02C03Z6, 02C03ZZ, 02C04Z6, 02C04ZZ, 02C10Z6, 02C10ZZ, 02C13Z6, 02C13ZZ, 02C14Z6, 02C14ZZ, 02C20Z6, 02C20ZZ, 02C23Z6, 02C23ZZ, 02C24Z6, 02C24ZZ, 02C30Z6, 02C30ZZ, 02C33Z6, 02C33ZZ, 02C34Z6, 02C34ZZ, 02CW0ZZ, 02CW3ZZ, 02CW4ZZ, 02CX0ZZ, 02CX3ZZ, 02CX4ZZ

© 2023 Wang Y et al. *JAMA Network Open.*

**PCI (CPT)**	92980, 92981, 92982, 92984, 92995, 92996, 92928, 92929, 92933, 92934, 92938, 92944, 92973, 92920, 92921, 92925, 92937, 92941, 92943
**ACS (ICD-9-CM)**	41000, 41001, 41002, 41010, 41011, 41012, 41020, 41021, 41022, 41030, 41031, 41032, 41040, 41041, 41042, 41050, 41051, 41052, 41060, 41061, 41062, 41070, 41071, 41072, 41080, 41081, 41082, 41090, 41091, 41092
**ACS (ICD-10-CM)**	I2101, I2102, I2109, I2111, I2119, I2121, I2129, I213, I214, I219, I21A1, I21A9, I220, I221, I222, I228, I229, I248, I249
**Clopidogrel (NDC)**	24117190, 93731405, 93731456, 93731498, 378362705, 378362777, 378362793, 904629461, 904646707, 13411011701, 13411011702, 13411011703, 13411011706, 13411011709, 13668014105, 13668014130, 13668014171, 13668014174, 13668014190, 16590028830, 16729021810, 16729021815, 16729021816, 16729021946, 16729021975, 21695066530, 21695066590, 31722090105, 31722090130, 31722090190, 33261052830, 33261098700, 33261098730, 33261098760, 33261098790, 33342006007, 33342006010, 33342006015, 42254023830, 42254023890, 42254039530, 42254039590, 42543071305, 42543071310, 42543071330, 42543071390, 43063037130, 43063037160, 43063037190, 47335089413, 47335089481, 47335089483, 49999040230, 49999040290, 49999086230, 50090208200, 50090208201, 50090259800, 50090259801, 50228012405, 50228012410, 50228012430, 50228012490, 50268018411, 50268018412, 51079055701, 51079055720, 51079055801, 51079055803, 51407003210, 51407003230, 51407003290, 54458088810, 54458088816, 54569470000, 54569470002, 54569581700, 54569632700, 54569632701, 54868407000, 54868407001, 54868407002, 54868554600, 55048007830, 55111019605, 55111019630, 55111019690, 55111067131, 55154337600, 55289091115, 55289091130, 55700057090, 58016001400, 58016001430, 58016001460, 58016001490, 58864074830, 58864086815, 60429011033, 60429030101, 60429030110, 60429030130, 60429030177, 60429030190, 60505025301, 60505025302, 60505025303, 60505025304, 60505353205, 60505399203, 60760073530, 60760073590, 61919000730, 61919073790, 62034001577, 63629159801, 63629159802, 63629159803, 63629159804, 63629472801, 63629472802, 63653117101, 63653117103, 63653117104, 63653117105, 63653117106, 63653133202, 63653133203, 63874056410, 63874056430, 63874056490, 64679031400, 64679031401, 64679031402, 64679031403, 64679031501, 64679031502, 65862035701, 65862035705, 65862035730, 65862035790, 65862035799, 66105011901, 66105011903, 66105011906, 66105011909, 66105011915, 66267069615, 66267069660, 66267129903, 68071069615, 68071153303, 68084053601, 68084053611, 68084053725, 68084053795, 68084060901, 68084075225, 68084075295, 68115085530, 68115085590, 68258600603, 68258607303, 68258607309, 68645044370, 68645059090, 68788770003, 68788770006, 68788770009, 69117101001, 69117101002, 69117101003, 69117101004, 69367019405, 69367019430, 70882011630, 70882012301, 71205007330, 71205007390, 71335008001, 71335008002, 71335008003, 71335008004, 71335058101, 71335058102, 71335058103, 71335058104, 71335075202, 71399750005, 71717010103, 71717010109, 71717010150, 76519101602

© 2023 Wang Y et al. *JAMA Network Open.*

**Prasugrel (NDC)**	2475901, 2475930, 2475977, 2476030, 2476076, 2512101, 2512130, 2512152, 2512301, 2512307, 2512330, 2512377, 378518593, 378518693, 440060430, 440060530, 16729027210, 16729027310, 51407024730, 51407024830, 54569627500, 54868623800, 60505464203, 60505464303, 65162000103, 65162000203, 65862082930, 65862083030, 66993057530, 66993057630, 67877060430, 67877060530
**Ticagrelor (NDC)**	186077660, 186077728, 186077739, 186077760

Note: *the decimal point was removed from the codes; ACS, Acute Coronary Syndrome; PCI, Percutaneous Coronary Intervention; NDC, National Drug Codes; CPT, Current Procedural Terminology

© 2023 Wang Y et al. *JAMA Network Open.*

eTable 2. Summary of ARC-HBR Items and Measurement Time Windows

Measurement items	Measurement time window
**Anti-coagulation use**	Having a prescription fill record in the past 3 months prior to day 0 and 30 days after the discharge
**Severe chronic renal disease**	Having a diagnosis or clinical visit in the past 12 months before day 0
**Anemia**	Having a diagnosis or clinical visit in the past 3 months before day 0
**Cirrhosis**	Having a diagnosis or clinical visit in the past 12 months before day 0
**Major bleeding events**	Having a diagnosis or clinical visit in the past 6 months before day 0
**Arteriovenous malformation**	Having a diagnosis or clinical visit in the past 12 months before day 0
**Ischemic stroke**	Having a diagnosis or clinical visit in the past 6 months before day 0
**Cancer**	Having a diagnosis or clinical visit in the past 12 months before day 0
**Thrombocytopenia**	Having a diagnosis or clinical visit in the past 12 months before day 0
**Major surgery**	Having a major surgery in the past 30 days prior to PCI admission date

© 2023 Wang Y et al. *JAMA Network Open.*

eTable 3. Codes for Different ARC-HBR Item Measurements

Item	Category	Code
**Cancer**	**ICD-9-CM**	140, 1401, 1403, 1404, 1405, 1406, 1408, 1409, 141, 1411, 1412, 1413, 1414, 1415, 1416, 1418, 1419, 142, 1421, 1422, 1428, 1429, 143, 1431, 1438, 1439, 144, 1441, 1448, 1449, 145, 1451, 1452, 1453, 1454, 1455, 1456, 1458, 1459, 146, 1461, 1462, 1463, 1464, 1465, 1466, 1467, 1468, 1469, 147, 1471, 1472, 1473, 1478, 1479, 148, 1481, 1482, 1483, 1488, 1489, 149, 1491, 1498, 1499, 150, 1501, 1502, 1503, 1504, 1505, 1508, 1509, 151, 1511, 1512, 1513, 1514, 1515, 1516, 1518, 1519, 152, 1521, 1522, 1523, 1528, 1529, 153, 1531, 1532, 1533, 1534, 1535, 1536, 1537, 1538, 1539, 154, 1541, 1542, 1543, 1548, 155, 1551, 1552, 156, 1561, 1562, 1568, 1569, 157, 1571, 1572, 1573, 1574, 1578, 1579, 158, 1588, 1589, 159, 1591, 1598, 1599, 160, 1601, 1602, 1603, 1604, 1605, 1608, 1609, 161, 1611, 1612, 1613, 1618, 1619, 162, 1622, 1623, 1624, 1625, 1628, 1629, 163, 1631, 1638, 1639, 164, 1641, 1642, 1643, 1648, 1649, 165, 1658, 1659, 170, 1701, 1702, 1703, 1704, 1705, 1706, 1707, 1708, 1709, 171, 1712, 1713, 1714, 1715, 1716, 1717, 1718, 1719, 172, 1721, 1722, 1723, 1724, 1725, 1726, 1727, 1728, 1729, 174, 1741, 1742, 1743, 1744, 1745, 1746, 1748, 1749, 175, 1759, 1761, 1762, 1763, 1764, 1765, 1768, 1769, 179, 180, 1801, 1808, 1809, 181, 182, 1821, 1828, 183, 1832, 1833, 1834, 1835, 1838, 1839, 184, 1841, 1842, 1843, 1844, 1848, 1849, 185, 186, 1869, 1871, 1872, 1873, 1874, 1875, 1876, 1877, 1878, 1879, 188, 1881, 1882, 1883, 1884, 1885, 1886, 1887, 1888, 1889, 189, 1891, 1892, 1893, 1894, 1898, 1899, 190, 1901, 1902, 1903, 1904, 1905, 1906, 1907, 1908, 1909, 191, 1911, 1912, 1913, 1914, 1915, 1916, 1917, 1918, 1919, 192, 1921, 1922, 1923, 1928, 1929, 193, 194, 1941, 1943, 1944, 1945, 1946, 1948, 1949, 195, 1951, 1952, 1953, 1954, 1955, 1958, 196, 1961, 1962, 1963, 1965, 1966, 1968, 1969, 197, 1971, 1972, 1973, 1974, 1975, 1976, 1977, 1978, 198, 1981, 1982, 1983, 1984, 1985, 1986, 1987, 19881, 19882, 19889, 199, 1991, 1992, 200, 20001, 20002, 20003, 20004, 20005, 20006, 20007, 20008, 2001, 20011, 20012, 20013, 20014, 20015, 20016, 20017, 20018, 2002, 20021, 20022, 20023, 20024, 20025, 20026, 20027, 20028, 2003, 20031, 20032, 20033, 20034, 20035, 20036, 20037, 20038, 2004, 20041, 20042, 20043, 20044, 20045, 20046, 20047, 20048, 2005, 20051, 20052, 20053, 20054, 20055, 20056, 20057, 20058, 2006, 20061, 20062, 20063, 20064, 20065, 20066, 20067, 20068, 2007, 20071, 20072, 20073, 20074, 20075, 20076, 20077, 20078, 2008, 20081, 20082, 20083, 20084, 20085, 20086, 20087, 20088, 201, 20101, 20102, 20103, 20104, 20105, 20106, 20107, 20108, 2011, 20111, 20112, 20113, 20114, 20115, 20116, 20117, 20118, 2012, 20121, 20122, 20123, 20124, 20125, 20126, 20127, 20128, 2014, 20141, 20142, 20143, 20144, 20145, 20146, 20147, 20148, 2015, 20151, 20152, 20153, 20154, 20155, 20156, 20157, 20158, 2016, 20161, 20162, 20163, 20164, 20165, 20166, 20167, 20168, 2017, 20171, 20172, 20173, 20174, 20175, 20176, 20177, 20178, 2019, 20191, 20192, 20193, 20194, 20195, 20196, 20197, 20198, 202, 20201, 20202, 20203, 20204, 20205, 20206, 20207, 20208, 2021, 20211, 20212, 20213, 20214, 20215, 20216, 20217, 20218, 2022, 20221, 20222, 20223, 20224, 20225, 20226, 20227, 20228, 2023, 20231, 20232, 20233, 20234, 20235, 20236, 20237, 20238, 2024,

© 2023 Wang Y et al. *JAMA Network Open.*

		20241, 20242, 20243, 20244, 20245, 20246, 20247, 20248, 2025, 20251, 20252, 20253, 20254, 20255, 20256, 20257, 20258, 2026, 20261, 20262, 20263, 20264, 20265, 20266, 20267, 20268, 2027, 20271, 20272, 20273, 20274, 20275, 20276, 20277, 20278, 2028, 20281, 20282, 20283, 20284, 20285, 20286, 20287, 20288, 2029, 20291, 20292, 20293, 20294, 20295, 20296, 20297, 20298, 203, 20301, 20302, 2031, 20311, 20312, 2038, 20381, 20382, 204, 20401, 20402, 2041, 20411, 20412, 2042, 20421, 20422, 2048, 20481, 20482, 2049, 20491, 20492, 205, 20501, 20502, 2051, 20511, 20512, 2052, 20521, 20522, 2053, 20531, 20532, 2058, 20581, 20582, 2059, 20591, 20592, 206, 20601, 20602, 2061, 20611, 20612, 2062, 20621, 20622, 2068, 20681, 20682, 2069, 20691, 20692, 207, 20701, 20702, 2071, 20711, 20712, 2072, 20721, 20722, 2078, 20781, 20782, 208, 20801, 20802, 2081, 20811, 20812, 2082, 20821, 20822, 2088, 20881, 20882, 2089, 20891, 20892, 209, 20901, 20902, 20903, 2091, 20911, 20912, 20913, 20914, 20915, 20916, 20917, 2092, 20921, 20922, 20923, 20924, 20925, 20926, 20927, 20929, 2093, 2097, 20971, 20972, 20973, 20974, 20979
	**ICD-10-CM**	C000, C001, C002, C003, C004, C005, C006, C008, C009, C01, C020, C021, C022, C023, C024, C028, C029, C030, C031, C039, C040, C041, C048, C049, C050, C051, C052, C058, C059, C060, C061, C062, C0680, C0689, C069, C07, C080, C081, C089, C090, C091, C098, C099, C100, C101, C102, C103, C104, C108, C109, C110, C111, C112, C113, C118, C119, C12, C130, C131, C132, C138, C139, C140, C142, C148, C153, C154, C155, C158, C159, C160, C161, C162, C163, C164, C165, C166, C168, C169, C170, C171, C172, C173, C178, C179, C180, C181, C182, C183, C184, C185, C186, C187, C188, C189, C19, C20, C210, C211, C212, C218, C220, C221, C222, C224, C227, C228, C229, C23, C240, C241, C248, C249, C250, C251, C252, C253, C254, C257, C258, C259, C260, C261, C269, C300, C301, C310, C311, C312, C313, C318, C319, C320, C321, C322, C323, C328, C329, C33, C3400, C3401, C3402, C3410, C3411, C3412, C342, C3430, C3431, C3432, C3480, C3481, C3482, C3490, C3491, C3492, C37, C380, C381, C382, C383, C384, C388, C390, C399, C4000, C4001, C4002, C4010, C4011, C4012, C4020, C4021, C4022, C4030, C4031, C4032, C4080, C4081, C4082, C4090, C4091, C4092, C410, C411, C412, C413, C414, C419, C430, C4310, C4311, C4312, C4320, C4321, C4322, C4330, C4331, C4339, C434, C4351, C4352, C4359, C4360, C4361, C4362, C4370, C4371, C4372, C438, C439, C450, C451, C452, C457, C459, C461, C462, C463, C464, C4650, C4651, C4652, C467, C469, C470, C4710, C4711, C4712, C4720, C4721, C4722, C473, C474, C475, C476, C478, C479, C480, C481, C482, C488, C490, C4910, C4911, C4912, C4920, C4921, C4922, C493, C494, C495, C496, C498, C499, C49A0, C49A1, C49A2, C49A3, C49A4, C49A5, C49A9, C50011, C50012, C50019, C50021, C50022, C50029, C50111, C50112, C50119, C50121, C50122, C50129, C50211, C50212, C50219, C50221, C50222, C50229, C50311, C50312, C50319, C50321, C50322, C50329, C50411, C50412, C50419, C50421, C50422, C50429, C50511, C50512, C50519, C50521, C50522, C50529, C50611, C50612, C50619, C50621, C50622, C50629, C50811, C50812, C50819, C50821, C50822, C50829, C50911, C50912, C50919, C50921, C50922, C50929, C510, C511, C512, C518, C519, C52, C530, C531, C538, C539, C540,

© 2023 Wang Y et al. *JAMA Network Open.*

		C541, C542, C543, C548, C549, C55, C561, C562, C569, C5700, C5701, C5702, C5710, C5711, C5712, C5720, C5721, C5722, C573, C574, C577, C578, C579, C58, C600, C601, C602, C608, C609, C61, C6200, C6201, C6202, C6210, C6211, C6212, C6290, C6291, C6292, C6300, C6301, C6302, C6310, C6311, C6312, C632, C637, C638, C639, C641, C642, C649, C651, C652, C659, C661, C662, C669, C670, C671, C672, C673, C674, C675, C676, C677, C678, C679, C680, C681, C688, C689, C6900, C6901, C6902, C6910, C6911, C6912, C6920, C6921, C6922, C6930, C6931, C6932, C6940, C6941, C6942, C6950, C6951, C6952, C6960, C6961, C6962, C6980, C6981, C6982, C6990, C6991, C6992, C700, C701, C709, C710, C711, C712, C713, C714, C715, C716, C717, C718, C719, C720, C721, C7220, C7221, C7222, C7230, C7231, C7232, C7240, C7241, C7242, C7250, C7259, C729, C73, C7400, C7401, C7402, C7410, C7411, C7412, C7490, C7491, C7492, C750, C751, C752, C753, C754, C755, C758, C759, C760, C761, C762, C763, C7640, C7641, C7642, C7650, C7651, C7652, C768, C770, C771, C772, C773, C774, C775, C778, C779, C7800, C7801, C7802, C781, C782, C7830, C7839, C784, C785, C786, C787, C7880, C7889, C7900, C7901, C7902, C7910, C7911, C7919, C792, C7931, C7932, C7940, C7949, C7951, C7952, C7960, C7961, C7962, C7970, C7971, C7972, C7981, C7982, C7989, C799, C7A00, C7A010, C7A011, C7A012, C7A019, C7A020, C7A021, C7A022, C7A023, C7A024, C7A025, C7A026, C7A029, C7A090, C7A091, C7A092, C7A093, C7A094, C7A095, C7A096, C7A098, C7A1, C7A8, C7B00, C7B01, C7B02, C7B03, C7B04, C7B09, C7B8, C800, C801, C802, C8100, C8101, C8102, C8103, C8104, C8105, C8106, C8107, C8108, C8109, C8110, C8111, C8112, C8113, C8114, C8115, C8116, C8117, C8118, C8119, C8120, C8121, C8122, C8123, C8124, C8125, C8126, C8127, C8128, C8129, C8130, C8131, C8132, C8133, C8134, C8135, C8136, C8137, C8138, C8139, C8140, C8141, C8142, C8143, C8144, C8145, C8146, C8147, C8148, C8149, C8170, C8171, C8172, C8173, C8174, C8175, C8176, C8177, C8178, C8179, C8190, C8191, C8192, C8193, C8194, C8195, C8196, C8197, C8198, C8199, C8200, C8201, C8202, C8203, C8204, C8205, C8206, C8207, C8208, C8209, C8210, C8211, C8212, C8213, C8214, C8215, C8216, C8217, C8218, C8219, C8220, C8221, C8222, C8223, C8224, C8225, C8226, C8227, C8228, C8229, C8230, C8231, C8232, C8233, C8234, C8235, C8236, C8237, C8238, C8239, C8240, C8241, C8242, C8243, C8244, C8245, C8246, C8247, C8248, C8249, C8250, C8251, C8252, C8253, C8254, C8255, C8256, C8257, C8258, C8259, C8260, C8261, C8262, C8263, C8264, C8265, C8266, C8267, C8268, C8269, C8280, C8281, C8282, C8283, C8284, C8285, C8286, C8287, C8288, C8289, C8290, C8291, C8292, C8293, C8294, C8295, C8296, C8297, C8298, C8299, C8300, C8301, C8302, C8303, C8304, C8305, C8306, C8307, C8308, C8309, C8310, C8311, C8312, C8313, C8314, C8315, C8316, C8317, C8318, C8319, C8330, C8331, C8332, C8333, C8334, C8335, C8336, C8337, C8338, C8339, C8350, C8351, C8352, C8353, C8354, C8355, C8356, C8357, C8358, C8359, C8370, C8371, C8372, C8373, C8374, C8375, C8376, C8377, C8378, C8379, C8380, C8381, C8382, C8383, C8384, C8385, C8386, C8387, C8388, C8389, C8390, C8391, C8392, C8393, C8394, C8395, C8396, C8397, C8398, C8399, C8400, C8401, C8402, C8403, C8404, C8405, C8406, C8407, C8408, C8409, C8410, C8411, C8412, C8413,

© 2023 Wang Y et al. *JAMA Network Open.*

		C8414, C8415, C8416, C8417, C8418, C8419, C8440, C8441, C8442, C8443, C8444, C8445, C8446, C8447, C8448, C8449, C8460, C8461, C8462, C8463, C8464, C8465, C8466, C8467, C8468, C8469, C8470, C8471, C8472, C8473, C8474, C8475, C8476, C8477, C8478, C8479, C8490, C8491, C8492, C8493, C8494, C8495, C8496, C8497, C8498, C8499, C84Z0, C84Z1, C84Z2, C84Z3, C84Z4, C84Z5, C84Z6, C84Z7, C84Z8, C84Z9, C8510, C8511, C8512, C8513, C8514, C8515, C8516, C8517, C8518, C8519, C8520, C8521, C8522, C8523, C8524, C8525, C8526, C8527, C8528, C8529, C8580, C8581, C8582, C8583, C8584, C8585, C8586, C8587, C8588, C8589, C8590, C8591, C8592, C8593, C8594, C8595, C8596, C8597, C8598, C8599, C860, C861, C862, C863, C864, C865, C866, C880, C882, C883, C884, C888, C889, C9000, C9001, C9002, C9010, C9011, C9012, C9020, C9021, C9022, C9030, C9031, C9032, C9100, C9101, C9102, C9110, C9111, C9112, C9130, C9131, C9132, C9140, C9141, C9142, C9150, C9151, C9152, C9160, C9161, C9162, C9190, C9191, C9192, C91A0, C91A1, C91A2, C91Z0, C91Z1, C91Z2, C9200, C9201, C9202, C9210, C9211, C9212, C9220, C9221, C9222, C9230, C9231, C9232, C9240, C9241, C9242, C9250, C9251, C9252, C9260, C9261, C9262, C9290, C9291, C9292, C92A0, C92A1, C92A2, C92Z0, C92Z1, C92Z2, C9300, C9301, C9302, C9310, C9311, C9312, C9330, C9331, C9332, C9390, C9391, C9392, C93Z0, C93Z1, C93Z2, C9400, C9401, C9402, C9420, C9421, C9422, C9430, C9431, C9432, C9440, C9441, C9442, C946, C9480, C9481, C9482, C9500, C9501, C9502, C9510, C9511, C9512, C9590, C9591, C9592, C960, C962, C9620, C9621, C9622, C9629, C964, C965, C966, C969, C96A, C96Z
**Thromboc ytopenia**	**ICD-9-CM**	2873, 2874, 2875
	**ICD-10-CM**	D693, D694, D695, D696
**Major bleeding**	**ICD-9-CM**	430, 431, 4320, 4329, 4321, 4389, 4380, 43811, 43812, 48313, 43814, 43819, 43831, 43832, 43830, 43841, 43842, 43840, 43821, 43822, 43820, 43851, 43852, 43850, 43853, 43881, 43882, 43883, 43884, 43889, 4389, 85241, 9070, 85242, 85243, 85244, 85245, 85246, 85221, 85222, 85223, 85224, 85225, 85226, 85201, 85202, 85203, 85204, 85205, 85206, 85301, 85302, 85303, 85304, 85305, 85306, 85142, 85162, 85143, 85163, 85144, 85164, 85145, 85165, 85146, 85166, 4560, 4561, 4562, 4568, 5780, 53784, 53100, 53120, 53140, 53160, 53200, 53220, 53240, 53260, 53300, 53320, 53340, 53360, 53400, 53420, 53440, 53460, 53501, 53531, 53511, 53541, 53521, 53551, 53561, 53021, 5307, 5781, 56985, 5550, 5551, 5552, 5559, 5566, 5562, 5563, 5564, 5565, 5568, 5569, 56203, 5695, 56202, 56212, 56213, 56986, 5693, 5812, 5813, 5811, 58189, 5819, 59971, 59972, 59970, 6021, 7848, 7847, 78630, 78631, 78639, 36281, 37742, 36361, 36362, 36372, 37272, 37643, 37923, 570, 6264, 6269, 6262, 6266, 6263, 6265, 6270, 6268, 6267, 6271, 6272, 6273, 6278, 6279, 2867, 28652, 28653, 28659, 2869, 2851, 5789, 23871
	**ICD-10-CM**	I60, I61, I62, I690, I691, I692, S064, S065, S066, S0634, S0635, S0636, S0637, S0638, I85, I864, K920, K3182, K250, K252, K254, K256, K260, K262, K264, K266, K270, K272, K274, K276, K280, K282, K284, K286, K2901, K2921, K2931, K2941, K2951, K2961, K2971, K2981, K2991, K2081,

© 2023 Wang Y et al. *JAMA Network Open.*

		K209, K2101, K2211, K226, K921, K5521, K50011, K50111, K50811, K50911, K51011, K51211, K51311, K51411, K51511, K51811, K51911, K5701, K5711, K5713, K5731, K5733, K5741, K5751, K5753, K5781, K5791, K5793, K6381, K625, N02, R31, N421, R04, H356, H4702, H3130, H3131, H3141, H113, H0532, H431, K762, N92, N930, N938, N939, N950, D683, D699, D62, K922, D473, D683
**Stroke**	**ICD-9-CM**	36234, 430, 431, 432, 433, 434, 435, 436, 437, 438
	**ICD-10-CM**	G45, G46, H340, I60, I61, I62, I63, I64, I65, I66, I67, I68, I69
**Arteriovenous malformation**	**ICD-9-CM**	5712, 5715
	**ICD-10-CM**	K746, K703
**Cirrhosis**	**ICD-9-CM**	5712, 5715, 5716, 5724, 56723, 5723, 7892, 4561, 45621, 4560, 45620, 34830, 34831, 34839, 78959, 5722, 7824, 53082
	**ICD-10-CM**	I8501, I8511, G9340, G9341, G9349, R188, K7290, K7291, R17, I1864, K703, K740, K746, K743, K744, K745, K767, K652, K766, R161, I8500, I8510
**Anemia**	**ICD-9-CM**	2800, 2801, 2808, 2809, 2810, 2811, 2812, 2813, 2814, 2818, 2819, 2820, 2821, 2822, 2823, 28240, 28241, 28242, 28243, 28244, 28245, 28246, 28247, 28249, 2825, 28260, 28261, 28262, 28263, 28264, 28268, 28269, 2827, 2828, 2829, 2830, 28310, 28311, 28319, 2832, 2839, 28401, 28409, 28411, 28412, 28419, 2842, 28481, 28489, 2849, 2850, 2851, 28521, 28522, 28529, 2853, 2858, 2859
	**ICD-10-CM**	D500, D501, D508, D509, D510, D511, D512, D513, D518, D519, D520, D521, D528, D529, D530, D531, D532, D538, D539, D550, D551, D552, D553, D558, D559, D560, D561, D562, D563, D564, D565, D568, D569, D5700, D5701, D5702, D5703, D5709, D571, D5720, D57211, D57212, D57213, D57218, D57219, D573, D5740, D57411, D57412, D57413, D57418, D57419, D5742, D57431, D57432, D57433, D57438, D57439, D5744, D57451, D57452, D57453, D57458, D57459, D5780, D57811, D57812, D57813, D57818, D57819, D580, D581, D582, D588, D589, D590, D591, D5910, D5911, D5912, D5913, D5919, D592, D593, D594, D595, D596, D598, D599, D600, D601, D608, D609, D6101, D6109, D611, D612, D613, D61810, D61811, D61818, D6182, D6189, D619, D62, D630, D631, D638, D640, D641, D642, D643, D644, D6481, D6489, D649
**Severe chronic renal disease**	**ICD-9-CM**	01600, 01601, 01602, 01603, 01604, 01605, 01606, 0954, 1890, 1899, 2230, 23691, 24940, 24941, 25040, 25041, 25042, 25043, 2714, 27410, 28311, 40301, 40311, 40391, 40402, 40403, 40412, 40413, 40492, 40493, 4401, 4421, 5724, 5800, 5804, 58081, 58089, 5809, 5810, 5811, 5812, 5813, 58181, 58189, 5819, 5820, 5821, 5822, 5824, 58281, 58289, 5829, 5830, 5831, 5832, 5834, 5836, 5837, 58381, 58389, 5839, 5845, 5846, 5847, 5848, 5849, 5851, 5852, 5853, 5854, 5855, 5856, 5859, 586, 587, 5880, 5881, 58881, 58889, 5889, 591, 75312, 75313, 75314, 75315, 75316, 75317, 75319, 75320, 75321, 75322, 75323, 75329, 7944

© 2023 Wang Y et al. *JAMA Network Open.*

	**ICD-10-CM**	A1811, A5275, B520, C641, C642, C649, C689, D3000, D3001, D3002, D4100, D4101, D4102, D4110, D4111, D4112, D4120, D4121, D4122, D593, E0821, E0822, E0829, E0865, E0921, E0922, E0929, E1021, E1022, E1029, E1065, E1121, E1122, E1129, E1165, E1321, E1322, E1329, E748, I120, I129, I130, I1310, I1311, I132, I701, I722, K767, M1030, M10311, M10312, M10319, M10321, M10322, M10329, M10331, M10332, M10339, M10341, M10342, M10349, M10351, M10352, M10359, M10361, M10362, M10369, M10371, M10372, M10379, M1038, M1039, M3214, M3215, M3504, N000, N001, N002, N003, N004, N005, N006, N007, N008, N009, N00A, N010, N011, N012, N013, N014, N015, N016, N017, N018, N019, N01A, N020, N021, N022, N023, N024, N025, N026, N027, N028, N029, N02A, N030, N031, N032, N033, N034, N035, N036, N037, N038, N039, N03A, N040, N041, N042, N043, N044, N045, N046, N047, N048, N049, N04A, N050, N051, N052, N053, N054, N055, N056, N057, N058, N059, N05A, N060, N061, N062, N063, N064, N065, N066, N067, N068, N069, N06A, N070, N071, N072, N073, N074, N075, N076, N077, N078, N079, N07A, N08, N131, N132, N1330, N1339, N140, N141, N142, N143, N144, N150, N158, N159, N16, N170, N171, N172, N178, N179, N181, N182, N183, N1830, N1831, N1832, N184, N185, N186, N189, N19, N250, N251, N2581, N2589, N259, N261, N269, Q6102, Q6111, Q6119, Q612, Q613, Q614, Q615, Q618, Q620, Q622, Q6210, Q6211, Q6212, Q6231, Q6232, Q6239, R944
**Major surgery**	**ICD-9-PCS**	0050, 0051, 0052, 0053, 0054, 0056, 0057, 0061, 0062, 0066, 0070, 0071, 0072, 0073, 0080, 0081, 0082, 0083, 0084, 0085, 0086, 0087, 0112, 0114, 0115, 0118, 0119, 0120, 0121, 0122, 0123, 0124, 0125, 0128, 0129, 0131, 0132, 0139, 0141, 0142, 0151, 0152, 0153, 0159, 016 , 0201, 0202, 0203, 0204, 0205, 0206, 0207, 0211, 0212, 0213, 0214, 022 , 0221, 0222, 0231, 0232, 0233, 0234, 0235, 0239, 0242, 0243, 0291, 0292, 0293, 0294, 0299, 0301, 0302, 0309, 031 , 0321, 0329, 0332, 0339, 034 , 0351, 0352, 0353, 0359, 036 , 0371, 0372, 0379, 0393, 0394, 0397, 0398, 0399, 0401, 0402, 0403, 0404, 0405, 0406, 0407, 0412, 0419, 043 , 0441, 0442, 0443, 0444, 0449, 045 , 046 , 0471, 0472, 0473, 0474, 0475, 0476, 0479, 0491, 0492, 0493, 0499, 050 , 0511, 0519, 0521, 0522, 0523, 0524, 0525, 0529, 0581, 0589, 059 , 0602, 0609, 0612, 0613, 0619, 062 , 0631, 0639, 064 , 0650, 0651, 0652, 066 , 067 , 0681, 0689, 0691, 0692, 0693, 0694, 0695, 0698, 0699, 0700, 0701, 0702, 0712, 0713, 0714, 0715, 0716, 0717, 0719, 0721, 0722, 0729, 073 , 0741, 0742, 0743, 0744, 0745, 0749, 0751, 0752, 0753, 0754, 0759, 0761, 0762, 0763, 0764, 0765, 0768, 0769, 0771, 0772, 0779, 0780, 0781, 0782, 0783, 0784, 0791, 0792, 0793, 0794, 0795, 0798, 0799, 0811, 0820, 0821, 0822, 0823, 0824, 0825, 0831, 0832, 0833, 0834, 0835, 0836, 0837, 0838, 0841, 0842, 0843, 0844, 0849, 0851, 0852, 0859, 0861, 0862, 0863, 0864, 0869, 0870, 0871, 0872, 0873, 0874, 0891, 0892, 0893, 0899, 090 , 0911, 0912, 0919, 0920, 0921, 0922, 0923, 093 , 0941, 0942, 0943, 0944, 0949, 0951, 0952, 0953, 0959, 096 , 0971, 0972, 0973, 0981, 0982, 0983, 0991, 0999, 100 , 101 , 1021, 1029, 1031, 1032, 1033, 1041, 1042, 1043, 1044, 1049, 105 , 106 , 1091, 1099, 110 , 111 , 1121, 1122, 1129, 1131, 1132, 1139, 1141, 1142, 1143, 1149, 1151, 1152, 1153, 1159, 1160, 1161, 1162, 1163, 1164, 1169, 1171, 1172, 1173, 1174, 1175, 1176, 1179, 1191, 1192, 1199, 1200, 1201, 1202, 1211,

© 2023 Wang Y et al. *JAMA Network Open.*

		1212, 1213, 1214, 1221, 1222, 1229, 1231, 1232, 1233, 1234, 1235, 1239, 1240, 1241, 1242, 1243, 1244, 1251, 1252, 1253, 1254, 1255, 1259, 1261, 1262, 1263, 1264, 1265, 1266, 1267, 1269, 1271, 1272, 1273, 1274, 1279, 1281, 1282, 1283, 1284, 1285, 1286, 1287, 1288, 1289, 1291, 1292, 1293, 1297, 1298, 1299, 1300, 1301, 1302, 1311, 1319, 132 , 133 , 1341, 1342, 1343, 1351, 1359, 1361, 1362, 1363, 1364, 1365, 1366, 1369, 1370, 1371, 1372, 138 , 139 , 1390, 1391, 1400, 1401, 1402, 1411, 1419, 1421, 1422, 1426, 1427, 1429, 1431, 1432, 1439, 1441, 1449, 1451, 1452, 1453, 1454, 1455, 1459, 146 , 1471, 1472, 1473, 1474, 1475, 1479, 1481, 1482, 1483, 149 , 1501, 1509, 1511, 1512, 1513, 1519, 1521, 1522, 1529, 153 , 154 , 155 , 156 , 157 , 159 , 1601, 1602, 1609, 161 , 1622, 1623, 1629, 1631, 1639, 1641, 1642, 1649, 1651, 1652, 1659, 1661, 1662, 1663, 1664, 1665, 1666, 1669, 1671, 1672, 1681, 1682, 1689, 1692, 1693, 1698, 1699, 1711, 1712, 1713, 1721, 1722, 1723, 1724, 1731, 1732, 1733, 1734, 1735, 1736, 1739, 1751, 1752, 1753, 1754, 1755, 1756, 1761, 1762, 1763, 1769, 1821, 1831, 1839, 185 , 186 , 1871, 1872, 1879, 189 , 190 , 1911, 1919, 1921, 1929, 193 , 194 , 1952, 1953, 1954, 1955, 196 , 199 , 2001, 2021, 2022, 2023, 2032, 2039, 2041, 2042, 2049, 2051, 2059, 2061, 2062, 2071, 2072, 2079, 2091, 2092, 2093, 2095, 2096, 2097, 2098, 2099, 2104, 2105, 2106, 2107, 2109, 214 , 215 , 2161, 2162, 2169, 2172, 2182, 2183, 2184, 2185, 2186, 2187, 2188, 2189, 2199, 2212, 2231, 2239, 2241, 2242, 2250, 2251, 2252, 2253, 2260, 2261, 2262, 2263, 2264, 2271, 2279, 229 , 242 , 244 , 245 , 2502, 251 , 252 , 253 , 254 , 2559, 2594, 2599, 2612, 2621, 2629, 2630, 2631, 2632, 2641, 2642, 2649, 2699, 270 , 271 , 2721, 2722, 2731, 2732, 2742, 2743, 2749, 2753, 2754, 2755, 2756, 2757, 2759, 2761, 2762, 2763, 2769, 2771, 2772, 2773, 2779, 2792, 2799, 280 , 2811, 2819, 282 , 283 , 284 , 285 , 286 , 287 , 2891, 2892, 2899, 290 , 292 , 293 , 2931, 2932, 2933, 2939, 294 , 2951, 2952, 2953, 2954, 2959, 2992, 2999, 3001, 3009, 301 , 3021, 3022, 3029, 303 , 304 , 3121, 3129, 313 , 3145, 315 , 3161, 3162, 3163, 3164, 3169, 3171, 3172, 3173, 3174, 3175, 3179, 3191, 3192, 3198, 3199, 320 , 3209, 321 , 3220, 3221, 3222, 3223, 3224, 3225, 3226, 3227, 3229, 323 , 3230, 3239, 324 , 3241, 3249, 325 , 3250, 3259, 326 , 329 , 330 , 331 , 3320, 3325, 3327, 3328, 3329, 3334, 3339, 3341, 3342, 3343, 3348, 3349, 335 , 3350, 3351, 3352, 336 , 3392, 3393, 3398, 3399, 3402, 3403, 3406, 341 , 3420, 3421, 3422, 3426, 3427, 3428, 3429, 343 , 344 , 3451, 3452, 3459, 346 , 3473, 3474, 3479, 3481, 3482, 3483, 3484, 3485, 3489, 3493, 3499, 3500, 3501, 3502, 3503, 3504, 3505, 3506, 3507, 3508, 3509, 3510, 3511, 3512, 3513, 3514, 3520, 3521, 3522, 3523, 3524, 3525, 3526, 3527, 3528, 3531, 3532, 3533, 3534, 3535, 3539, 3542, 3550, 3551, 3552, 3553, 3554, 3555, 3560, 3561, 3562, 3563, 3570, 3571, 3572, 3573, 3581, 3582, 3583, 3584, 3591, 3592, 3593, 3594, 3595, 3596, 3597, 3598, 3599, 3600, 3601, 3602, 3603, 3605, 3609, 3610, 3611, 3612, 3613, 3614, 3615, 3616, 3617, 3619, 362 , 363 , 3631, 3632, 3633, 3634, 3639, 3691, 3699, 3710, 3711, 3712, 3724, 3731, 3732, 3733, 3734, 3735, 3737, 374 , 3741, 3749, 375 , 3751, 3752, 3753, 3754, 3755, 3760, 3761, 3762, 3763, 3764, 3765, 3766, 3767, 3768, 3774, 3775, 3776, 3777, 3779, 3780, 3785, 3786, 3787, 3789, 3791, 3794, 3795, 3796, 3797, 3798, 3799, 3800, 3801, 3802, 3803, 3804, 3805, 3806, 3807, 3808, 3809, 3810, 3811, 3812, 3813, 3814, 3815, 3816,

© 2023 Wang Y et al. *JAMA Network Open.*

		3818, 3821, 3826, 3829, 3830, 3831, 3832, 3833, 3834, 3835, 3836, 3837, 3838, 3839, 3840, 3841, 3842, 3843, 3844, 3845, 3846, 3847, 3848, 3849, 3850, 3851, 3852, 3853, 3855, 3857, 3859, 3860, 3861, 3862, 3863, 3864, 3865, 3866, 3867, 3868, 3869, 387 , 3880, 3881, 3882, 3883, 3884, 3885, 3886, 3887, 3888, 3889, 390 , 391 , 3921, 3922, 3923, 3924, 3925, 3926, 3927, 3928, 3929, 3930, 3931, 3932, 3941, 3942, 3943, 3949, 3950, 3951, 3952, 3953, 3954, 3955, 3956, 3957, 3958, 3959, 3965, 397 , 3971, 3972, 3973, 3974, 3975, 3976, 3977, 3978, 3979, 398 , 3981, 3982, 3983, 3984, 3985, 3986, 3987, 3988, 3989, 3991, 3992, 3993, 3994, 3998, 3999, 400 , 4011, 4019, 4021, 4022, 4023, 4024, 4029, 403 , 4040, 4041, 4042, 4050, 4051, 4052, 4053, 4054, 4059, 4061, 4062, 4063, 4064, 4069, 409 , 410 , 4100, 4101, 4102, 4103, 4104, 4105, 4106, 4107, 4108, 4109, 412 , 4133, 4141, 4142, 4143, 415 , 4193, 4194, 4195, 4199, 4201, 4209, 4210, 4211, 4212, 4219, 4221, 4225, 4231, 4232, 4239, 4240, 4241, 4242, 4251, 4252, 4253, 4254, 4255, 4256, 4258, 4259, 4261, 4262, 4263, 4264, 4265, 4266, 4268, 4269, 427 , 4282, 4283, 4284, 4285, 4286, 4287, 4289, 4291, 430 , 431 , 432 , 433 , 4342, 4349, 435 , 436 , 437 , 4381, 4382, 4389, 4391, 4399, 4400, 4401, 4402, 4403, 4411, 4415, 442 , 4421, 4429, 4431, 4432, 4438, 4439, 4440, 4441, 4442, 445 , 4461, 4463, 4464, 4465, 4466, 4467, 4468, 4469, 4491, 4492, 4495, 4496, 4497, 4498, 4499, 4500, 4501, 4502, 4503, 4511, 4515, 4521, 4526, 4531, 4532, 4533, 4534, 4541, 4549, 4550, 4551, 4552, 4561, 4562, 4563, 4571, 4572, 4573, 4574, 4575, 4576, 4579, 458 , 4581, 4582, 4583, 4590, 4591, 4592, 4593, 4594, 4595, 4601, 4602, 4603, 4604, 4610, 4611, 4612, 4613, 4620, 4621, 4622, 4623, 4640, 4641, 4642, 4643, 4650, 4651, 4652, 4660, 4661, 4662, 4663, 4664, 4671, 4672, 4673, 4674, 4675, 4676, 4679, 4680, 4681, 4682, 4691, 4692, 4693, 4694, 4697, 4699, 470 , 4701, 4709, 471 , 4711, 4719, 472 , 4791, 4792, 4799, 480 , 481 , 4821, 4825, 4835, 4840, 4841, 4842, 4843, 4849, 485 , 4850, 4851, 4852, 4859, 4861, 4862, 4863, 4864, 4865, 4866, 4869, 4871, 4872, 4873, 4874, 4875, 4876, 4879, 4881, 4882, 4891, 4892, 4893, 4899, 4901, 4902, 4904, 4911, 4912, 493 , 4939, 4944, 4945, 4946, 4949, 4951, 4952, 4959, 496 , 4971, 4972, 4973, 4974, 4975, 4976, 4979, 4991, 4992, 4993, 4994, 4995, 4999, 500 , 5012, 5014, 5019, 5021, 5022, 5023, 5024, 5025, 5026, 5029, 503 , 504 , 5051, 5059, 5061, 5069, 5102, 5103, 5104, 5113, 5119, 5121, 5122, 5123, 5124, 5131, 5132, 5133, 5134, 5135, 5136, 5137, 5139, 5141, 5142, 5143, 5149, 5151, 5159, 5161, 5162, 5163, 5169, 5171, 5172, 5179, 5181, 5182, 5183, 5189, 5191, 5192, 5193, 5194, 5195, 5199, 5201, 5209, 5212, 5219, 522 , 5222, 523 , 524 , 5251, 5252, 5253, 5259, 526 , 527 , 5280, 5281, 5282, 5283, 5291, 5292, 5295, 5296, 5299, 5300, 5301, 5302, 5303, 5304, 5305, 5310, 5311, 5312, 5313, 5314, 5315, 5316, 5317, 5321, 5329, 5331, 5339, 5341, 5342, 5343, 5349, 5351, 5359, 5361, 5362, 5363, 5369, 537 , 5371, 5372, 5375, 5380, 5381, 5382, 5383, 5384, 539 , 540 , 5411, 5412, 5419, 5421, 5422, 5423, 5429, 543 , 544 , 545 , 5451, 5459, 5461, 5462, 5463, 5464, 5471, 5472, 5473, 5474, 5475, 5492, 5493, 5494, 5495, 5501, 5502, 5503, 5504, 5511, 5512, 5524, 5529, 5531, 5532, 5533, 5534, 5535, 5539, 554 , 5551, 5552, 5553, 5554, 5561, 5569, 557 , 5581, 5582, 5583, 5584, 5585, 5586, 5587, 5589, 5591, 5597, 5598, 5599, 560 , 561 , 562 , 5634, 5639, 5640, 5641, 5642, 5651, 5652, 5661, 5662,

© 2023 Wang Y et al. *JAMA Network Open.*

		5671, 5672, 5673, 5674, 5675, 5679, 5681, 5682, 5683, 5684, 5685, 5686, 5689, 5692, 5693, 5694, 5695, 5699, 5712, 5718, 5719, 5721, 5722, 5733, 5734, 5739, 5741, 5749, 5751, 5759, 576 , 5771, 5779, 5781, 5782, 5783, 5784, 5785, 5786, 5787, 5788, 5789, 5791, 5793, 5796, 5797, 5798, 5799, 580 , 581 , 5841, 5842, 5843, 5844, 5845, 5846, 5847, 5849, 585 , 5891, 5892, 5893, 5899, 5900, 5901, 5902, 5903, 5909, 5911, 5912, 5919, 5921, 5929, 593 , 594 , 595 , 596 , 5971, 5979, 5991, 5992, 600 , 6012, 6014, 6015, 6018, 6019, 602 , 6021, 6029, 603 , 604 , 605 , 6061, 6062, 6069, 6072, 6073, 6079, 6081, 6082, 6093, 6094, 6095, 6096, 6097, 6099, 612 , 6142, 6149, 6192, 6199, 620 , 6212, 6219, 622 , 623 , 6241, 6242, 625 , 6261, 6269, 627 , 6299, 6309, 631 , 632 , 633 , 634 , 6351, 6353, 6359, 6381, 6382, 6383, 6385, 6389, 6392, 6393, 6394, 6395, 6399, 640 , 6411, 642 , 643 , 6441, 6442, 6443, 6444, 6445, 6449, 645 , 6492, 6493, 6495, 6496, 6497, 6498, 6499, 650 , 6501, 6509, 6511, 6512, 6513, 6514, 6519, 6521, 6522, 6523, 6524, 6525, 6529, 653 , 6531, 6539, 654 , 6541, 6549, 6551, 6552, 6553, 6554, 6561, 6562, 6563, 6564, 6571, 6572, 6573, 6574, 6575, 6576, 6579, 658 , 6581, 6589, 6591, 6592, 6593, 6594, 6595, 6599, 660 , 6601, 6602, 6611, 6619, 6621, 6622, 6629, 6631, 6632, 6639, 664 , 6651, 6652, 6661, 6662, 6663, 6669, 6671, 6672, 6673, 6674, 6679, 6692, 6693, 6694, 6695, 6696, 6697, 6699, 6711, 6712, 6719, 672 , 6731, 6732, 6733, 6739, 674 , 675 , 6751, 6759, 6761, 6762, 6769, 680 , 6813, 6814, 6815, 6816, 6819, 6821, 6822, 6823, 6824, 6825, 6829, 683 , 6831, 6839, 684 , 6841, 6849, 685 , 6851, 6859, 686 , 6861, 6869, 687 , 6871, 6879, 688 , 689 , 6901, 6902, 6909, 6911, 6919, 6921, 6922, 6923, 6929, 693 , 6941, 6942, 6949, 6951, 6952, 6995, 6997, 6998, 6999, 7012, 7013, 7014, 7023, 7024, 7029, 7031, 7032, 7033, 704 , 7050, 7051, 7052, 7053, 7054, 7055, 7061, 7062, 7063, 7064, 7071, 7072, 7073, 7074, 7075, 7076, 7077, 7078, 7079, 708 , 7091, 7092, 7093, 7101, 7109, 7111, 7119, 7122, 7123, 7124, 7129, 713 , 714 , 715 , 7161, 7162, 7171, 7172, 7179, 718 , 719 , 7394, 7399, 740 , 741 , 742 , 743 , 744 , 7491, 7499, 7536, 7550, 7551, 7552, 7561, 7593, 7599, 7601, 7609, 7611, 7619, 762 , 7631, 7639, 7641, 7642, 7643, 7644, 7645, 7646, 765 , 7661, 7662, 7663, 7664, 7665, 7666, 7667, 7668, 7669, 7670, 7672, 7674, 7676, 7677, 7679, 7691, 7692, 7694, 7697, 7699, 7700, 7701, 7702, 7703, 7704, 7705, 7706, 7707, 7708, 7709, 7710, 7711, 7712, 7713, 7714, 7715, 7716, 7717, 7718, 7719, 7720, 7721, 7722, 7723, 7724, 7725, 7726, 7727, 7728, 7729, 7730, 7731, 7732, 7733, 7734, 7735, 7736, 7737, 7738, 7739, 7740, 7741, 7742, 7743, 7744, 7745, 7746, 7747, 7748, 7749, 7751, 7752, 7753, 7754, 7756, 7757, 7758, 7759, 7760, 7761, 7762, 7763, 7764, 7765, 7766, 7767, 7768, 7769, 7770, 7771, 7772, 7773, 7774, 7775, 7776, 7777, 7778, 7779, 7780, 7781, 7782, 7783, 7784, 7785, 7786, 7787, 7788, 7789, 7790, 7791, 7792, 7793, 7794, 7795, 7796, 7797, 7798, 7799, 7800, 7801, 7802, 7803, 7804, 7805, 7806, 7807, 7808, 7809, 7810, 7811, 7812, 7813, 7814, 7815, 7816, 7817, 7818, 7819, 7820, 7822, 7823, 7824, 7825, 7827, 7828, 7829, 7830, 7831, 7832, 7833, 7834, 7835, 7837, 7838, 7839, 7840, 7841, 7842, 7843, 7844, 7845, 7846, 7847, 7848, 7849, 7850, 7851, 7852, 7853, 7854, 7855, 7856, 7857, 7858, 7859, 7860, 7861, 7862, 7863, 7864, 7865, 7866, 7867, 7868, 7869, 7870, 7871, 7872, 7873, 7874, 7875, 7876, 7877, 7878, 7879, 7880, 7881, 7882, 7883, 7884, 7885, 7886,

© 2023 Wang Y et al. *JAMA Network Open.*

		7887, 7888, 7889, 7890, 7891, 7892, 7893, 7894, 7895, 7896, 7897, 7898, 7899, 7910, 7911, 7912, 7913, 7914, 7915, 7916, 7917, 7918, 7919, 7920, 7921, 7922, 7923, 7924, 7925, 7926, 7927, 7928, 7929, 7930, 7931, 7932, 7933, 7934, 7935, 7936, 7937, 7938, 7939, 7940, 7941, 7942, 7945, 7946, 7949, 7950, 7951, 7952, 7955, 7956, 7959, 7960, 7961, 7962, 7963, 7964, 7965, 7966, 7967, 7968, 7969, 7980, 7981, 7982, 7983, 7984, 7985, 7986, 7987, 7988, 7989, 7990, 7991, 7992, 7993, 7994, 7995, 7996, 7997, 7998, 7999, 8000, 8001, 8002, 8003, 8004, 8005, 8006, 8007, 8008, 8009, 8010, 8011, 8012, 8013, 8014, 8015, 8016, 8017, 8018, 8019, 8020, 8021, 8022, 8023, 8024, 8025, 8026, 8027, 8028, 8029, 8040, 8041, 8042, 8043, 8044, 8045, 8046, 8047, 8048, 8049, 805 , 8050, 8051, 8053, 8054, 8059, 806 , 8070, 8071, 8072, 8073, 8074, 8075, 8076, 8077, 8078, 8079, 8080, 8081, 8082, 8083, 8084, 8085, 8086, 8087, 8088, 8089, 8090, 8091, 8092, 8093, 8094, 8095, 8096, 8097, 8098, 8099, 8100, 8101, 8102, 8103, 8104, 8105, 8106, 8107, 8108, 8109, 8111, 8112, 8113, 8114, 8115, 8116, 8117, 8118, 8120, 8121, 8122, 8123, 8124, 8125, 8126, 8127, 8128, 8129, 8130, 8131, 8132, 8133, 8134, 8135, 8136, 8137, 8138, 8139, 8140, 8141, 8142, 8143, 8144, 8145, 8146, 8147, 8148, 8149, 8151, 8152, 8153, 8154, 8155, 8156, 8157, 8159, 8161, 8162, 8163, 8164, 8165, 8166, 8169, 8171, 8172, 8173, 8174, 8175, 8179, 8180, 8181, 8182, 8183, 8184, 8185, 8186, 8187, 8188, 8193, 8194, 8195, 8196, 8197, 8198, 8199, 8201, 8202, 8203, 8209, 8211, 8212, 8219, 8221, 8222, 8229, 8231, 8232, 8233, 8234, 8235, 8236, 8239, 8241, 8242, 8243, 8244, 8245, 8246, 8251, 8252, 8253, 8254, 8255, 8256, 8257, 8258, 8259, 8261, 8269, 8271, 8272, 8279, 8281, 8282, 8283, 8284, 8285, 8286, 8289, 8291, 8299, 8301, 8302, 8303, 8309, 8311, 8312, 8313, 8314, 8319, 8321, 8329, 8331, 8332, 8339, 8341, 8342, 8343, 8344, 8345, 8349, 835 , 8361, 8362, 8363, 8364, 8365, 8371, 8372, 8373, 8374, 8375, 8376, 8377, 8379, 8381, 8382, 8383, 8384, 8385, 8386, 8387, 8388, 8389, 8391, 8392, 8393, 8399, 8400, 8401, 8402, 8403, 8404, 8405, 8406, 8407, 8408, 8409, 8410, 8411, 8412, 8413, 8414, 8415, 8416, 8417, 8418, 8419, 8421, 8422, 8423, 8424, 8425, 8426, 8427, 8428, 8429, 843 , 8440, 8444, 8448, 8458, 8459, 8460, 8461, 8462, 8463, 8464, 8465, 8466, 8467, 8468, 8469, 8480, 8481, 8482, 8483, 8484, 8485, 8491, 8492, 8493, 8494, 8499, 8512, 8520, 8521, 8522, 8523, 8524, 8525, 8531, 8532, 8533, 8534, 8535, 8536, 8541, 8542, 8543, 8544, 8545, 8546, 8547, 8548, 8550, 8553, 8554, 8555, 856 , 857 , 8570, 8571, 8572, 8573, 8574, 8575, 8576, 8579, 8582, 8583, 8584, 8585, 8586, 8587, 8589, 8593, 8594, 8595, 8596, 8599, 8606, 8621, 8622, 8625, 864 , 8660, 8661, 8662, 8663, 8665, 8666, 8667, 8669, 8670, 8671, 8672, 8673, 8674, 8675, 8681, 8682, 8683, 8684, 8685, 8686, 8687, 8689, 8690, 8691, 8693, 8694, 8695, 8696, 8697, 8698, 8753, 9227, 9504
	**ICD-10-PCS**	00900ZX, 00904ZX, 00910ZX, 00914ZX, 00920ZX, 00924ZX, 00930ZX, 00934ZX, 00940ZX, 00944ZX, 00950ZX, 00954ZX, 00960ZX, 00964ZX, 00970ZX, 00974ZX, 00980ZX, 00984ZX, 00990ZX, 00994ZX, 009A0ZX, 009A4ZX, 009B0ZX, 009B4ZX, 009C0ZX, 009C4ZX, 009D0ZX, 009D4ZX, 009F0ZX, 009F4ZX, 009G0ZX, 009G4ZX, 009H0ZX, 009H4ZX, 009J0ZX, 009J4ZX, 009K0ZX, 009K4ZX, 009L0ZX, 009L4ZX, 009M0ZX, 009M4ZX,

© 2023 Wang Y et al. *JAMA Network Open.*

		009N0ZX, 009N4ZX, 009P0ZX, 009P4ZX, 009Q0ZX, 009Q4ZX, 009R0ZX, 009R4ZX, 009S0ZX, 009S4ZX, 009T0ZX, 009T4ZX, 009U0ZX, 009U4ZX, 009W0ZX, 009W4ZX, 009X0ZX, 009X4ZX, 009Y0ZX, 009Y4ZX, 00B00ZX, 00B04ZX, 00B10ZX, 00B14ZX, 00B20ZX, 00B24ZX, 00B60ZX, 00B64ZX, 00B70ZX, 00B74ZX, 00B80ZX, 00B84ZX, 00B90ZX, 00B94ZX, 00BA0ZX, 00BA4ZX, 00BB0ZX, 00BB4ZX, 00BC0ZX, 00BC4ZX, 00BD0ZX, 00BD4ZX, 00BF0ZX, 00BF4ZX, 00BG0ZX, 00BG4ZX, 00BH0ZX, 00BH4ZX, 00BJ0ZX, 00BJ4ZX, 00BK0ZX, 00BK4ZX, 00BL0ZX, 00BL4ZX, 00BM0ZX, 00BM4ZX, 00BN0ZX, 00BN4ZX, 00BP0ZX, 00BP4ZX, 00BQ0ZX, 00BQ4ZX, 00BR0ZX, 00BR4ZX, 00BS0ZX, 00BS4ZX, 00BT0ZX, 00BT3ZX, 00BT4ZX, 00BW0ZX, 00BW3ZX, 00BW4ZX, 00BX0ZX, 00BX3ZX, 00BX4ZX, 00BY0ZX, 00BY3ZX, 00BY4ZX, 00H002Z, 00H042Z, 00H602Z, 00H642Z, 00HE02Z, 00HE42Z, 00HU02Z, 00HU42Z, 00HV02Z, 00HV42Z, 00J00ZZ, 00J04ZZ, 00JE0ZZ, 00JE4ZZ, 00JU0ZZ, 00JU4ZZ, 00JV0ZZ, 00JV4ZZ, 00K00ZZ, 00K03ZZ, 00K04ZZ, 00K70ZZ, 00K73ZZ, 00K74ZZ, 00K80ZZ, 00K83ZZ, 00K84ZZ, 00K90ZZ, 00K93ZZ, 00K94ZZ, 00KA0ZZ, 00KA3ZZ, 00KA4ZZ, 00KB0ZZ, 00KB3ZZ, 00KB4ZZ, 00KC0ZZ, 00KC3ZZ, 00KC4ZZ, 00KD0ZZ, 00KD3ZZ, 00KD4ZZ, 01900ZX, 01904ZX, 01910ZX, 01914ZX, 01920ZX, 01924ZX, 01930ZX, 01934ZX, 01940ZX, 01944ZX, 01950ZX, 01954ZX, 01960ZX, 01964ZX, 01980ZX, 01984ZX, 01990ZX, 01994ZX, 019A0ZX, 019A4ZX, 019B0ZX, 019B4ZX, 019C0ZX, 019C4ZX, 019D0ZX, 019D4ZX, 019F0ZX, 019F4ZX, 019G0ZX, 019G4ZX, 019H0ZX, 019H4ZX, 019K0ZX, 019K4ZX, 019L0ZX, 019L4ZX, 019M0ZX, 019M4ZX, 019N0ZX, 019N4ZX, 019P0ZX, 019P4ZX, 019Q0ZX, 019Q4ZX, 019R0ZX, 019R4ZX, 01B00ZX, 01B04ZX, 01B10ZX, 01B14ZX, 01B20ZX, 01B24ZX, 01B30ZX, 01B34ZX, 01B40ZX, 01B44ZX, 01B50ZX, 01B54ZX, 01B60ZX, 01B64ZX, 01B80ZX, 01B84ZX, 01B90ZX, 01B94ZX, 01BA0ZX, 01BA4ZX, 01BB0ZX, 01BB4ZX, 01BC0ZX, 01BC4ZX, 01BD0ZX, 01BD4ZX, 01BF0ZX, 01BF4ZX, 01BG0ZX, 01BG4ZX, 01BH0ZX, 01BH4ZX, 01BK0ZX, 01BK3ZX, 01BK4ZX, 01BL0ZX, 01BL3ZX, 01BL4ZX, 01BM0ZX, 01BM3ZX, 01BM4ZX, 01BN0ZX, 01BN3ZX, 01BN4ZX, 01BP0ZX, 01BP3ZX, 01BP4ZX, 01BQ0ZX, 01BQ4ZX, 01BR0ZX, 01BR4ZX, 01HY02Z, 01HY32Z, 01HY42Z, 01JY0ZZ, 01JY4ZZ, 02B44ZX, 02B54ZX, 02B64ZX, 02B74ZX, 02B84ZX, 02B94ZX, 02BD4ZX, 02BF4ZX, 02BG4ZX, 02BH4ZX, 02BJ4ZX, 02BK4ZX, 02BL4ZX, 02BM4ZX, 02BN0ZX, 02BN3ZX, 02BN4ZX, 02BP0ZX, 02BP3ZX, 02BP4ZX, 02BQ0ZX, 02BQ3ZX, 02BQ4ZX, 02BR0ZX, 02BR3ZX, 02BR4ZX, 02BS0ZX, 02BS3ZX, 02BS4ZX, 02BT0ZX, 02BT3ZX, 02BT4ZX, 02BV0ZX, 02BV3ZX, 02BV4ZX, 02BW0ZX, 02BW3ZX, 02BW4ZX, 02BX0ZX, 02BX3ZX, 02BX4ZX, 02H400Z, 02H402Z, 02H430Z, 02H440Z, 02H442Z, 02H600Z, 02H602Z, 02H630Z, 02H640Z, 02H642Z, 02H700Z, 02H702Z, 02H730Z, 02H740Z, 02H742Z, 02HK00Z, 02HK02Z, 02HK40Z, 02HK42Z, 02HL00Z, 02HL02Z, 02HL30Z, 02HL40Z, 02HL42Z, 02HN00Z, 02HN02Z, 02HN30Z, 02HN40Z,

© 2023 Wang Y et al. *JAMA Network Open.*

		02HN42Z, 02HP40Z, 02HP42Z, 02HQ40Z, 02HQ42Z, 02HR40Z, 02HR42Z, 02HS00Z, 02HS02Z, 02HS30Z, 02HS40Z, 02HS42Z, 02HT00Z, 02HT02Z, 02HT30Z, 02HT40Z, 02HT42Z, 02HV30Z, 02HV40Z, 02HV42Z, 02HW02Z, 02HW40Z, 02HW42Z, 02HX00Z, 02HX02Z, 02HX40Z, 02HX42Z, 02JA0ZZ, 02JA4ZZ, 02JY0ZZ, 02JY4ZZ, 02K84ZZ, 03900ZX, 03904ZX, 03910ZX, 03914ZX, 03920ZX, 03924ZX, 03930ZX, 03934ZX, 03940ZX, 03944ZX, 03950ZX, 03954ZX, 03960ZX, 03964ZX, 03970ZX, 03974ZX, 03980ZX, 03984ZX, 03990ZX, 03994ZX, 039A0ZX, 039A4ZX, 039B0ZX, 039B4ZX, 039C0ZX, 039C4ZX, 039D0ZX, 039D4ZX, 039F0ZX, 039F4ZX, 039G0ZX, 039G4ZX, 039H0ZX, 039H4ZX, 039J0ZX, 039J4ZX, 039K0ZX, 039K4ZX, 039L0ZX, 039L4ZX, 039M0ZX, 039M4ZX, 039N0ZX, 039N4ZX, 039P0ZX, 039P4ZX, 039Q0ZX, 039Q4ZX, 039R0ZX, 039R4ZX, 039S0ZX, 039S4ZX, 039T0ZX, 039T4ZX, 039U0ZX, 039U4ZX, 039V0ZX, 039V4ZX, 039Y0ZX, 039Y4ZX, 03B00ZX, 03B03ZX, 03B04ZX, 03B10ZX, 03B13ZX, 03B14ZX, 03B20ZX, 03B23ZX, 03B24ZX, 03B30ZX, 03B33ZX, 03B34ZX, 03B40ZX, 03B43ZX, 03B44ZX, 03B50ZX, 03B53ZX, 03B54ZX, 03B60ZX, 03B63ZX, 03B64ZX, 03B70ZX, 03B73ZX, 03B74ZX, 03B80ZX, 03B83ZX, 03B84ZX, 03B90ZX, 03B93ZX, 03B94ZX, 03BA0ZX, 03BA3ZX, 03BA4ZX, 03BB0ZX, 03BB3ZX, 03BB4ZX, 03BC0ZX, 03BC3ZX, 03BC4ZX, 03BD0ZX, 03BD3ZX, 03BD4ZX, 03BF0ZX, 03BF3ZX, 03BF4ZX, 03BG0ZX, 03BG3ZX, 03BG4ZX, 03BH0ZX, 03BH3ZX, 03BH4ZX, 03BJ0ZX, 03BJ3ZX, 03BJ4ZX, 03BK0ZX, 03BK3ZX, 03BK4ZX, 03BL0ZX, 03BL3ZX, 03BL4ZX, 03BM0ZX, 03BM3ZX, 03BM4ZX, 03BN0ZX, 03BN3ZX, 03BN4ZX, 03BP0ZX, 03BP3ZX, 03BP4ZX, 03BQ0ZX, 03BQ3ZX, 03BQ4ZX, 03BR0ZX, 03BR3ZX, 03BR4ZX, 03BS0ZX, 03BS3ZX, 03BS4ZX, 03BT0ZX, 03BT3ZX, 03BT4ZX, 03BU0ZX, 03BU3ZX, 03BU4ZX, 03BV0ZX, 03BV3ZX, 03BV4ZX, 03BY0ZX, 03BY3ZX, 03BY4ZX, 03HY02Z, 03HY42Z, 03JY0ZZ, 03JY4ZZ, 04900ZX, 04904ZX, 04910ZX, 04914ZX, 04920ZX, 04924ZX, 04930ZX, 04934ZX, 04940ZX, 04944ZX, 04950ZX, 04954ZX, 04960ZX, 04964ZX, 04970ZX, 04974ZX, 04980ZX, 04984ZX, 04990ZX, 04994ZX, 049A0ZX, 049A4ZX, 049B0ZX, 049B4ZX, 049C0ZX, 049C4ZX, 049D0ZX, 049D4ZX, 049E0ZX, 049E4ZX, 049F0ZX, 049F4ZX, 049H0ZX, 049H4ZX, 049J0ZX, 049J4ZX, 049K0ZX, 049K4ZX, 049L0ZX, 049L4ZX, 049M0ZX, 049M4ZX, 049N0ZX, 049N4ZX, 049P0ZX, 049P4ZX, 049Q0ZX, 049Q4ZX, 049R0ZX, 049R4ZX, 049S0ZX, 049S4ZX, 049T0ZX, 049T4ZX, 049U0ZX, 049U4ZX, 049V0ZX, 049V4ZX, 049W0ZX, 049W4ZX, 049Y0ZX, 049Y4ZX, 04B00ZX, 04B03ZX, 04B04ZX, 04B10ZX, 04B13ZX, 04B14ZX, 04B20ZX, 04B23ZX, 04B24ZX, 04B30ZX, 04B33ZX, 04B34ZX, 04B40ZX, 04B43ZX, 04B44ZX, 04B50ZX, 04B53ZX, 04B54ZX, 04B60ZX, 04B63ZX, 04B64ZX, 04B70ZX, 04B73ZX, 04B74ZX, 04B80ZX, 04B83ZX, 04B84ZX, 04B90ZX, 04B93ZX, 04B94ZX, 04BA0ZX, 04BA3ZX, 04BA4ZX, 04BB0ZX, 04BB3ZX, 04BB4ZX, 04BC0ZX, 04BC3ZX, 04BC4ZX, 04BD0ZX, 04BD3ZX, 04BD4ZX, 04BE0ZX, 04BE3ZX,

© 2023 Wang Y et al. *JAMA Network Open.*

		04BE4ZX, 04BF0ZX, 04BF3ZX, 04BF4ZX, 04BH0ZX, 04BH3ZX, 04BH4ZX, 04BJ0ZX, 04BJ3ZX, 04BJ4ZX, 04BK0ZX, 04BK3ZX, 04BK4ZX, 04BL0ZX, 04BL3ZX, 04BL4ZX, 04BM0ZX, 04BM3ZX, 04BM4ZX, 04BN0ZX, 04BN3ZX, 04BN4ZX, 04BP0ZX, 04BP3ZX, 04BP4ZX, 04BQ0ZX, 04BQ3ZX, 04BQ4ZX, 04BR0ZX, 04BR3ZX, 04BR4ZX, 04BS0ZX, 04BS3ZX, 04BS4ZX, 04BT0ZX, 04BT3ZX, 04BT4ZX, 04BU0ZX, 04BU3ZX, 04BU4ZX, 04BV0ZX, 04BV3ZX, 04BV4ZX, 04BW0ZX, 04BW3ZX, 04BW4ZX, 04BY0ZX, 04BY3ZX, 04BY4ZX, 04H042Z, 04HY02Z, 04HY42Z, 04JY0ZZ, 04JY4ZZ, 05900ZX, 05904ZX, 05910ZX, 05914ZX, 05930ZX, 05934ZX, 05940ZX, 05944ZX, 05950ZX, 05954ZX, 05960ZX, 05964ZX, 05970ZX, 05974ZX, 05980ZX, 05984ZX, 05990ZX, 05994ZX, 059A0ZX, 059A4ZX, 059B0ZX, 059B4ZX, 059C0ZX, 059C4ZX, 059D0ZX, 059D4ZX, 059F0ZX, 059F4ZX, 059G0ZX, 059G4ZX, 059H0ZX, 059H4ZX, 059L0ZX, 059L4ZX, 059M0ZX, 059M4ZX, 059N0ZX, 059N4ZX, 059P0ZX, 059P4ZX, 059Q0ZX, 059Q4ZX, 059R0ZX, 059R4ZX, 059S0ZX, 059S4ZX, 059T0ZX, 059T4ZX, 059V0ZX, 059V4ZX, 059Y0ZX, 059Y4ZX, 05B00ZX, 05B03ZX, 05B04ZX, 05B10ZX, 05B13ZX, 05B14ZX, 05B30ZX, 05B33ZX, 05B34ZX, 05B40ZX, 05B43ZX, 05B44ZX, 05B50ZX, 05B53ZX, 05B54ZX, 05B60ZX, 05B63ZX, 05B64ZX, 05B70ZX, 05B73ZX, 05B74ZX, 05B80ZX, 05B83ZX, 05B84ZX, 05B90ZX, 05B93ZX, 05B94ZX, 05BA0ZX, 05BA3ZX, 05BA4ZX, 05BB0ZX, 05BB3ZX, 05BB4ZX, 05BC0ZX, 05BC3ZX, 05BC4ZX, 05BD0ZX, 05BD3ZX, 05BD4ZX, 05BF0ZX, 05BF3ZX, 05BF4ZX, 05BG0ZX, 05BG3ZX, 05BG4ZX, 05BH0ZX, 05BH3ZX, 05BH4ZX, 05BL0ZX, 05BL3ZX, 05BL4ZX, 05BM0ZX, 05BM3ZX, 05BM4ZX, 05BN0ZX, 05BN3ZX, 05BN4ZX, 05BP0ZX, 05BP3ZX, 05BP4ZX, 05BQ0ZX, 05BQ3ZX, 05BQ4ZX, 05BR0ZX, 05BR3ZX, 05BR4ZX, 05BS0ZX, 05BS3ZX, 05BS4ZX, 05BT0ZX, 05BT3ZX, 05BT4ZX, 05BV0ZX, 05BV3ZX, 05BV4ZX, 05BY0ZX, 05BY3ZX, 05BY4ZX, 05H002Z, 05H042Z, 05HY02Z, 05HY42Z, 05JY0ZZ, 05JY4ZZ, 06900ZX, 06904ZX, 06910ZX, 06914ZX, 06920ZX, 06924ZX, 06930ZX, 06934ZX, 06940ZX, 06944ZX, 06950ZX, 06954ZX, 06960ZX, 06964ZX, 06970ZX, 06974ZX, 06980ZX, 06984ZX, 06990ZX, 06994ZX, 069B0ZX, 069B4ZX, 069C0ZX, 069C4ZX, 069D0ZX, 069D4ZX, 069F0ZX, 069F4ZX, 069G0ZX, 069G4ZX, 069H0ZX, 069H4ZX, 069J0ZX, 069J4ZX, 069M0ZX, 069M4ZX, 069N0ZX, 069N4ZX, 069P0ZX, 069P4ZX, 069Q0ZX, 069Q4ZX, 069R0ZX, 069R4ZX, 069S0ZX, 069S4ZX, 069T0ZX, 069T4ZX, 069V0ZX, 069V4ZX, 069Y0ZX, 069Y4ZX, 06B00ZX, 06B03ZX, 06B04ZX, 06B10ZX, 06B13ZX, 06B14ZX, 06B20ZX, 06B23ZX, 06B24ZX, 06B30ZX, 06B33ZX, 06B34ZX, 06B40ZX, 06B43ZX, 06B44ZX, 06B50ZX, 06B53ZX, 06B54ZX, 06B60ZX, 06B63ZX, 06B64ZX, 06B70ZX, 06B73ZX, 06B74ZX, 06B80ZX, 06B83ZX, 06B84ZX, 06B90ZX, 06B93ZX, 06B94ZX, 06BB0ZX, 06BB3ZX, 06BB4ZX, 06BC0ZX, 06BC3ZX, 06BC4ZX, 06BD0ZX, 06BD3ZX, 06BD4ZX, 06BF0ZX, 06BF3ZX, 06BF4ZX, 06BG0ZX, 06BG3ZX, 06BG4ZX, 06BH0ZX, 06BH3ZX, 06BH4ZX, 06BJ0ZX, 06BJ3ZX,

© 2023 Wang Y et al. *JAMA Network Open.*

		06BJ4ZX, 06BM0ZX, 06BM3ZX, 06BM4ZX, 06BN0ZX, 06BN3ZX, 06BN4ZX, 06BP0ZX, 06BP3ZX, 06BP4ZX, 06BQ0ZX, 06BQ3ZX, 06BQ4ZX, 06BR0ZX, 06BR3ZX, 06BR4ZX, 06BS0ZX, 06BS3ZX, 06BS4ZX, 06BT0ZX, 06BT3ZX, 06BT4ZX, 06BV0ZX, 06BV3ZX, 06BV4ZX, 06BY0ZX, 06BY3ZX, 06BY4ZX, 06HY02Z, 06HY42Z, 06JY0ZZ, 06JY4ZZ, 07900ZX, 07904ZX, 07910ZX, 07914ZX, 07920ZX, 07924ZX, 07930ZX, 07934ZX, 07940ZX, 07944ZX, 07950ZX, 07954ZX, 07960ZX, 07964ZX, 07970ZX, 07974ZX, 07980ZX, 07984ZX, 07990ZX, 07994ZX, 079B0ZX, 079B4ZX, 079C0ZX, 079C4ZX, 079D0ZX, 079D4ZX, 079F0ZX, 079F4ZX, 079G0ZX, 079G4ZX, 079H0ZX, 079H4ZX, 079J0ZX, 079J4ZX, 079K0ZX, 079K4ZX, 079L0ZX, 079L4ZX, 079M0ZX, 079M4ZX, 079P0ZX, 079P4ZX, 079T4ZX, 07B00ZX, 07B03ZX, 07B04ZX, 07B10ZX, 07B13ZX, 07B14ZX, 07B20ZX, 07B23ZX, 07B24ZX, 07B30ZX, 07B33ZX, 07B34ZX, 07B40ZX, 07B43ZX, 07B44ZX, 07B50ZX, 07B53ZX, 07B54ZX, 07B60ZX, 07B63ZX, 07B64ZX, 07B70ZX, 07B73ZX, 07B74ZX, 07B80ZX, 07B83ZX, 07B84ZX, 07B90ZX, 07B93ZX, 07B94ZX, 07BB0ZX, 07BB3ZX, 07BB4ZX, 07BC0ZX, 07BC3ZX, 07BC4ZX, 07BD0ZX, 07BD3ZX, 07BD4ZX, 07BF0ZX, 07BF3ZX, 07BF4ZX, 07BG0ZX, 07BG3ZX, 07BG4ZX, 07BH0ZX, 07BH3ZX, 07BH4ZX, 07BJ0ZX, 07BJ3ZX, 07BJ4ZX, 07BK0ZX, 07BK3ZX, 07BK4ZX, 07BL0ZX, 07BL3ZX, 07BL4ZX, 07BM0ZX, 07BM3ZX, 07BM4ZX, 07BP0ZX, 07BP4ZX, 07D04ZX, 07D14ZX, 07D24ZX, 07D34ZX, 07D44ZX, 07D54ZX, 07D64ZX, 07D74ZX, 07D84ZX, 07D94ZX, 07DB4ZX, 07DC4ZX, 07DD4ZX, 07DF4ZX, 07DG4ZX, 07DH4ZX, 07DJ4ZX, 07DK4ZX, 07DL4ZX, 07DM4ZX, 07DP4ZX, 07JK0ZZ, 07JK4ZZ, 07JL0ZZ, 07JL4ZZ, 07JM0ZZ, 07JM4ZZ, 07JN0ZZ, 07JN4ZZ, 07JP0ZZ, 07JP4ZZ, 07JT4ZZ, 08923ZX, 08933ZX, 08943ZX, 08953ZX, 089A0ZX, 089A3ZX, 089B0ZX, 089B3ZX, 089C3ZX, 089D3ZX, 089E3ZX, 089F3ZX, 089G3ZX, 089H3ZX, 089J3ZX, 089K3ZX, 089L0ZX, 089L3ZX, 089M0ZX, 089M3ZX, 089N0ZX, 089P0ZX, 089Q0ZX, 089R0ZX, 089V0ZX, 089V3ZX, 089W0ZX, 089W3ZX, 089X0ZX, 089X3ZX, 089X7ZX, 089X8ZX, 089Y0ZX, 089Y3ZX, 089Y7ZX, 089Y8ZX, 08B00ZX, 08B03ZX, 08B0XZX, 08B10ZX, 08B13ZX, 08B1XZX, 08B43ZX, 08B53ZX, 08B6XZX, 08B7XZX, 08B8XZX, 08B9XZX, 08BA0ZX, 08BA3ZX, 08BB0ZX, 08BB3ZX, 08BC3ZX, 08BD3ZX, 08BE3ZX, 08BF3ZX, 08BJ3ZX, 08BK3ZX, 08BL0ZX, 08BL3ZX, 08BM0ZX, 08BM3ZX, 08BN0ZX, 08BN3ZX, 08BNXZX, 08BP0ZX, 08BP3ZX, 08BPXZX, 08BQ0ZX, 08BQ3ZX, 08BQXZX, 08BR0ZX, 08BR3ZX, 08BRXZX, 08BSXZX, 08BTXZX, 08BV0ZX, 08BV3ZX, 08BW0ZX, 08BW3ZX, 08BX0ZX, 08BX3ZX, 08BX7ZX, 08BX8ZX, 08BY0ZX, 08BY3ZX, 08BY7ZX, 08BY8ZX, 08D8XZX, 08D9XZX, 08JL0ZZ, 08JM0ZZ, 09904ZX, 09914ZX, 09934ZX, 09944ZX, 09950ZX, 09960ZX, 09970ZX, 09973ZX, 09974ZX, 09980ZX, 09983ZX, 09984ZX, 09990ZX, 09997ZX, 09998ZX, 099A0ZX, 099A7ZX, 099A8ZX, 099B0ZX, 099B4ZX, 099B7ZX, 099B8ZX, 099C0ZX, 099C4ZX, 099C7ZX, 099C8ZX, 099D0ZX, 099D7ZX, 099D8ZX, 099E0ZX,

© 2023 Wang Y et al. *JAMA Network Open.*

		099E7ZX, 099E8ZX, 099F0ZX, 099F4ZX, 099G0ZX, 099G4ZX, 099K4ZX, 099L4ZX, 099M4ZX, 099N4ZX, 099P0ZX, 099P4ZX, 099Q0ZX, 099Q4ZX, 099R0ZX, 099R4ZX, 099S0ZX, 099S4ZX, 099T0ZX, 099T4ZX, 099U0ZX, 099U4ZX, 099V0ZX, 099V4ZX, 099W0ZX, 099W4ZX, 099X0ZX, 099X4ZX, 09B04ZX, 09B14ZX, 09B34ZX, 09B44ZX, 09B50ZX, 09B58ZX, 09B60ZX, 09B68ZX, 09B70ZX, 09B73ZX, 09B74ZX, 09B77ZX, 09B78ZX, 09B80ZX, 09B83ZX, 09B84ZX, 09B87ZX, 09B88ZX, 09B90ZX, 09B98ZX, 09BA0ZX, 09BA8ZX, 09BB0ZX, 09BB3ZX, 09BB4ZX, 09BB8ZX, 09BC0ZX, 09BC3ZX, 09BC4ZX, 09BC8ZX, 09BD0ZX, 09BD8ZX, 09BE0ZX, 09BE8ZX, 09BF4ZX, 09BG4ZX, 09BK4ZX, 09BL4ZX, 09BM4ZX, 09BN4ZX, 09BP0ZX, 09BP4ZX, 09BQ0ZX, 09BQ4ZX, 09BR0ZX, 09BR4ZX, 09BS0ZX, 09BS4ZX, 09BT0ZX, 09BT4ZX, 09BU0ZX, 09BU4ZX, 09BV0ZX, 09BV4ZX, 09BW0ZX, 09BW4ZX, 09BX0ZX, 09BX4ZX, 09J70ZZ, 09J74ZZ, 09J80ZZ, 09J84ZZ, 09JD0ZZ, 09JD4ZZ, 09JE0ZZ, 09JE4ZZ, 09JH4ZZ, 09JJ4ZZ, 09JK4ZZ, 09JY4ZZ, 0B910ZX, 0B914ZX, 0B920ZX, 0B924ZX, 0B930ZX, 0B934ZX, 0B940ZX, 0B944ZX, 0B950ZX, 0B954ZX, 0B960ZX, 0B964ZX, 0B970ZX, 0B974ZX, 0B980ZX, 0B984ZX, 0B990ZX, 0B994ZX, 0B9B0ZX, 0B9B4ZX, 0B9C0ZX, 0B9C4ZX, 0B9D0ZX, 0B9D4ZX, 0B9F0ZX, 0B9F4ZX, 0B9G0ZX, 0B9G4ZX, 0B9H0ZX, 0B9H4ZX, 0B9J0ZX, 0B9J4ZX, 0B9K0ZX, 0B9K4ZX, 0B9L0ZX, 0B9L4ZX, 0B9M0ZX, 0B9M4ZX, 0B9N4ZX, 0B9P4ZX, 0B9R0ZX, 0B9R4ZX, 0B9S0ZX, 0B9S4ZX, 0B9T0ZX, 0B9T4ZX, 0BB10ZX, 0BB14ZX, 0BB20ZX, 0BB24ZX, 0BB30ZX, 0BB34ZX, 0BB40ZX, 0BB44ZX, 0BB50ZX, 0BB54ZX, 0BB60ZX, 0BB64ZX, 0BB70ZX, 0BB74ZX, 0BB80ZX, 0BB84ZX, 0BB90ZX, 0BB94ZX, 0BBB0ZX, 0BBB4ZX, 0BBC0ZX, 0BBC4ZX, 0BBC7ZX, 0BBC8ZX, 0BBD0ZX, 0BBD4ZX, 0BBD7ZX, 0BBD8ZX, 0BBF0ZX, 0BBF4ZX, 0BBF7ZX, 0BBF8ZX, 0BBG0ZX, 0BBG4ZX, 0BBG7ZX, 0BBG8ZX, 0BBH0ZX, 0BBH4ZX, 0BBH7ZX, 0BBH8ZX, 0BBJ0ZX, 0BBJ4ZX, 0BBJ7ZX, 0BBJ8ZX, 0BBK0ZX, 0BBK4ZX, 0BBK7ZX, 0BBK8ZX, 0BBL0ZX, 0BBL4ZX, 0BBL7ZX, 0BBL8ZX, 0BBM0ZX, 0BBM4ZX, 0BBM7ZX, 0BBM8ZX, 0BBN4ZX, 0BBN8ZX, 0BBP4ZX, 0BBP8ZX, 0BBR0ZX, 0BBR3ZX, 0BBR4ZX, 0BBS0ZX, 0BBS3ZX, 0BBS4ZX, 0BBT0ZX, 0BBT3ZX, 0BBT4ZX, 0BD14ZX, 0BD24ZX, 0BD34ZX, 0BD44ZX, 0BD54ZX, 0BD64ZX, 0BD74ZX, 0BD84ZX, 0BD94ZX, 0BDB4ZX, 0BDC4ZX, 0BDD4ZX, 0BDF4ZX, 0BDG4ZX, 0BDH4ZX, 0BDJ4ZX, 0BDK4ZX, 0BDL4ZX, 0BDM4ZX, 0BDN0ZX, 0BDN3ZX, 0BDN4ZX, 0BDP0ZX, 0BDP3ZX, 0BDP4ZX, 0BH002Z, 0BH032Z, 0BH042Z, 0BH102Z, 0BHK02Z, 0BHK32Z, 0BHK42Z, 0BHL02Z, 0BHL32Z, 0BHL42Z, 0BHR02Z, 0BHR32Z, 0BHR42Z, 0BHS02Z, 0BHS32Z, 0BHS42Z, 0BHT02Z, 0BHT32Z, 0BHT42Z, 0BJ00ZZ, 0BJ04ZZ, 0BJ10ZZ, 0BJ14ZZ, 0BJK0ZZ, 0BJK4ZZ, 0BJL0ZZ, 0BJL4ZZ, 0BJQ0ZZ, 0BJQ4ZZ, 0BJT0ZZ, 0BJT4ZZ, 0C920ZX, 0C92XZX, 0C930ZX, 0C93XZX, 0C970ZX, 0C980ZX, 0C990ZX, 0C9B0ZX, 0C9C0ZX, 0C9D0ZX, 0C9F0ZX, 0C9G0ZX, 0C9H0ZX, 0C9J0ZX, 0C9M4ZX, 0C9N0ZX, 0C9NXZX, 0C9P0ZX, 0C9PXZX, 0C9Q0ZX,

© 2023 Wang Y et al. *JAMA Network Open.*

		0C9QXZX, 0C9R0ZX, 0C9R4ZX, 0C9S0ZX, 0C9S4ZX, 0C9T0ZX, 0C9T4ZX, 0C9V0ZX, 0C9V4ZX, 0CB20ZX, 0CB23ZX, 0CB2XZX, 0CB30ZX, 0CB33ZX, 0CB3XZX, 0CB70ZX, 0CB80ZX, 0CB90ZX, 0CBB0ZX, 0CBC0ZX, 0CBD0ZX, 0CBF0ZX, 0CBG0ZX, 0CBH0ZX, 0CBJ0ZX, 0CBM4ZX, 0CBN0ZX, 0CBN3ZX, 0CBNXZX, 0CBP0ZX, 0CBP3ZX, 0CBPXZX, 0CBQ0ZX, 0CBQ3ZX, 0CBQXZX, 0CBR0ZX, 0CBR4ZX, 0CBS0ZX, 0CBS4ZX, 0CBT0ZX, 0CBT4ZX, 0CBV0ZX, 0CBV4ZX, 0CJS4ZZ, 0CJY4ZZ, 0D910ZX, 0D914ZX, 0D920ZX, 0D924ZX, 0D930ZX, 0D934ZX, 0D940ZX, 0D944ZX, 0D950ZX, 0D954ZX, 0D960ZX, 0D964ZX, 0D970ZX, 0D974ZX, 0D980ZX, 0D984ZX, 0D990ZX, 0D994ZX, 0D9A0ZX, 0D9A4ZX, 0D9B0ZX, 0D9B4ZX, 0D9C0ZX, 0D9C4ZX, 0D9E0ZX, 0D9E4ZX, 0D9F0ZX, 0D9F4ZX, 0D9G0ZX, 0D9G4ZX, 0D9H0ZX, 0D9H4ZX, 0D9J0ZX, 0D9J4ZX, 0D9J7ZX, 0D9J8ZX, 0D9K0ZX, 0D9K4ZX, 0D9L0ZX, 0D9L4ZX, 0D9M0ZX, 0D9M4ZX, 0D9N0ZX, 0D9N4ZX, 0D9P0ZX, 0D9P4ZX, 0D9Q4ZX, 0D9R4ZX, 0D9S0ZX, 0D9S4ZX, 0D9T0ZX, 0D9T4ZX, 0D9U0ZX, 0D9U4ZX, 0D9V0ZX, 0D9V4ZX, 0D9W0ZX, 0D9W4ZX, 0DB10ZX, 0DB14ZX, 0DB20ZX, 0DB24ZX, 0DB30ZX, 0DB34ZX, 0DB40ZX, 0DB44ZX, 0DB50ZX, 0DB54ZX, 0DB60ZX, 0DB64ZX, 0DB70ZX, 0DB74ZX, 0DB80ZX, 0DB84ZX, 0DB90ZX, 0DB94ZX, 0DBA0ZX, 0DBA4ZX, 0DBB0ZX, 0DBB4ZX, 0DBC0ZX, 0DBC4ZX, 0DBE0ZX, 0DBE4ZX, 0DBF0ZX, 0DBF4ZX, 0DBG0ZX, 0DBG4ZX, 0DBH0ZX, 0DBH4ZX, 0DBJ0ZX, 0DBJ3ZX, 0DBJ4ZX, 0DBJ7ZX, 0DBJ8ZX, 0DBK0ZX, 0DBK4ZX, 0DBL0ZX, 0DBL4ZX, 0DBM0ZX, 0DBM4ZX, 0DBN0ZX, 0DBN4ZX, 0DBP0ZX, 0DBP4ZX, 0DBQ4ZX, 0DBR4ZX, 0DBS0ZX, 0DBT0ZX, 0DBU0ZX, 0DBU4ZX, 0DBV0ZX, 0DBV4ZX, 0DBW0ZX, 0DBW4ZX, 0DD14ZX, 0DD24ZX, 0DD34ZX, 0DD44ZX, 0DD54ZX, 0DD64ZX, 0DD74ZX, 0DD84ZX, 0DD94ZX, 0DDA4ZX, 0DDB4ZX, 0DDC4ZX, 0DDE4ZX, 0DDF4ZX, 0DDG4ZX, 0DDH4ZX, 0DDJ3ZX, 0DDJ4ZX, 0DDJ8ZX, 0DDK4ZX, 0DDL4ZX, 0DDM4ZX, 0DDN4ZX, 0DDP4ZX, 0DDQ4ZX, 0DH502Z, 0DH532Z, 0DH542Z, 0DH602Z, 0DH632Z, 0DH642Z, 0DH802Z, 0DH832Z, 0DH842Z, 0DH902Z, 0DH932Z, 0DH942Z, 0DHA02Z, 0DHA32Z, 0DHA42Z, 0DHB02Z, 0DHB32Z, 0DHB42Z, 0DJ00ZZ, 0DJ04ZZ, 0DJ60ZZ, 0DJ64ZZ, 0DJD0ZZ, 0DJD4ZZ, 0DJU0ZZ, 0DJU4ZZ, 0DJV0ZZ, 0DJV4ZZ, 0DJW0ZZ, 0DJW4ZZ, 0F900ZX, 0F904ZX, 0F910ZX, 0F914ZX, 0F920ZX, 0F924ZX, 0F940ZX, 0F944ZX, 0F950ZX, 0F954ZX, 0F960ZX, 0F964ZX, 0F970ZX, 0F974ZX, 0F980ZX, 0F984ZX, 0F990ZX, 0F994ZX, 0F9C0ZX, 0F9C4ZX, 0F9D0ZX, 0F9D4ZX, 0F9F0ZX, 0F9F4ZX, 0F9G0ZX, 0F9G4ZX, 0FB00ZX, 0FB04ZX, 0FB10ZX, 0FB14ZX, 0FB20ZX, 0FB24ZX, 0FB40ZX, 0FB44ZX, 0FB50ZX, 0FB54ZX, 0FB60ZX, 0FB64ZX, 0FB70ZX, 0FB74ZX, 0FB80ZX, 0FB84ZX, 0FB90ZX, 0FB94ZX, 0FBC0ZX, 0FBC4ZX, 0FBD0ZX, 0FBD4ZX, 0FBF0ZX, 0FBF4ZX, 0FBG0ZX, 0FBG4ZX, 0FD04ZX, 0FD14ZX, 0FD24ZX, 0FD44ZX, 0FD54ZX, 0FD64ZX, 0FD74ZX, 0FD84ZX, 0FD94ZX, 0FDC4ZX, 0FDD4ZX, 0FDF4ZX, 0FDG4ZX, 0FH002Z, 0FH032Z, 0FH042Z, 0FH102Z, 0FH132Z, 0FH142Z, 0FH202Z, 0FH232Z,

© 2023 Wang Y et al. *JAMA Network Open.*

		0FH242Z, 0FH402Z, 0FH432Z, 0FH442Z, 0FHB02Z, 0FHB32Z, 0FHB42Z, 0FHD02Z, 0FHD32Z, 0FHD42Z, 0FHG02Z, 0FHG32Z, 0FHG42Z, 0FJ00ZZ, 0FJ04ZZ, 0FJ40ZZ, 0FJ44ZZ, 0FJB0ZZ, 0FJB4ZZ, 0FJD0ZZ, 0FJD4ZZ, 0FJG0ZZ, 0FJG4ZZ, 0G900ZX, 0G904ZX, 0G910ZX, 0G914ZX, 0G920ZX, 0G924ZX, 0G930ZX, 0G934ZX, 0G940ZX, 0G944ZX, 0G964ZX, 0G974ZX, 0G984ZX, 0G994ZX, 0G9B4ZX, 0G9C4ZX, 0G9D4ZX, 0G9F4ZX, 0G9G0ZX, 0G9G4ZX, 0G9H0ZX, 0G9H4ZX, 0G9K0ZX, 0G9K4ZX, 0G9L0ZX, 0G9L4ZX, 0G9M0ZX, 0G9M4ZX, 0G9N0ZX, 0G9N4ZX, 0G9P0ZX, 0G9P4ZX, 0G9Q0ZX, 0G9Q4ZX, 0G9R0ZX, 0G9R4ZX, 0GB00ZX, 0GB03ZX, 0GB04ZX, 0GB10ZX, 0GB13ZX, 0GB14ZX, 0GB20ZX, 0GB24ZX, 0GB30ZX, 0GB34ZX, 0GB40ZX, 0GB44ZX, 0GB64ZX, 0GB74ZX, 0GB84ZX, 0GB94ZX, 0GBB4ZX, 0GBC4ZX, 0GBD4ZX, 0GBF4ZX, 0GBG0ZX, 0GBG4ZX, 0GBH0ZX, 0GBH4ZX, 0GBJ0ZX, 0GBJ4ZX, 0GBL0ZX, 0GBL3ZX, 0GBL4ZX, 0GBM0ZX, 0GBM3ZX, 0GBM4ZX, 0GBN0ZX, 0GBN3ZX, 0GBN4ZX, 0GBP0ZX, 0GBP3ZX, 0GBP4ZX, 0GBQ0ZX, 0GBQ3ZX, 0GBQ4ZX, 0GBR0ZX, 0GBR3ZX, 0GBR4ZX, 0GHS02Z, 0GHS32Z, 0GHS42Z, 0GJ00ZZ, 0GJ04ZZ, 0GJ10ZZ, 0GJ14ZZ, 0GJ50ZZ, 0GJ54ZZ, 0GJK0ZZ, 0GJK4ZZ, 0GJR0ZZ, 0GJR4ZZ, 0GJS0ZZ, 0GJS4ZZ, 0H9T0ZX, 0H9U0ZX, 0H9V0ZX, 0H9W0ZX, 0H9X0ZX, 0HBT0ZX, 0HBU0ZX, 0HBV0ZX, 0HBW0ZX, 0HBX0ZX, 0HBY0ZX, 0JH600Z, 0JH630Z, 0JH800Z, 0JH830Z, 0K900ZX, 0K904ZX, 0K910ZX, 0K914ZX, 0K920ZX, 0K924ZX, 0K930ZX, 0K934ZX, 0K940ZX, 0K944ZX, 0K950ZX, 0K954ZX, 0K960ZX, 0K964ZX, 0K970ZX, 0K974ZX, 0K980ZX, 0K984ZX, 0K990ZX, 0K994ZX, 0K9B0ZX, 0K9B4ZX, 0K9C0ZX, 0K9C4ZX, 0K9D0ZX, 0K9D4ZX, 0K9F0ZX, 0K9F4ZX, 0K9G0ZX, 0K9G4ZX, 0K9H0ZX, 0K9H4ZX, 0K9J0ZX, 0K9J4ZX, 0K9K0ZX, 0K9K4ZX, 0K9L0ZX, 0K9L4ZX, 0K9M0ZX, 0K9M4ZX, 0K9N0ZX, 0K9N4ZX, 0K9P0ZX, 0K9P4ZX, 0K9Q0ZX, 0K9Q4ZX, 0K9R0ZX, 0K9R4ZX, 0K9S0ZX, 0K9S4ZX, 0K9T0ZX, 0K9T4ZX, 0K9V0ZX, 0K9V4ZX, 0K9W0ZX, 0K9W4ZX, 0KB00ZX, 0KB03ZX, 0KB04ZX, 0KB10ZX, 0KB13ZX, 0KB14ZX, 0KB20ZX, 0KB23ZX, 0KB24ZX, 0KB30ZX, 0KB33ZX, 0KB34ZX, 0KB40ZX, 0KB43ZX, 0KB44ZX, 0KB50ZX, 0KB53ZX, 0KB54ZX, 0KB60ZX, 0KB63ZX, 0KB64ZX, 0KB70ZX, 0KB73ZX, 0KB74ZX, 0KB80ZX, 0KB83ZX, 0KB84ZX, 0KB90ZX, 0KB93ZX, 0KB94ZX, 0KBB0ZX, 0KBB3ZX, 0KBB4ZX, 0KBC0ZX, 0KBC3ZX, 0KBC4ZX, 0KBD0ZX, 0KBD3ZX, 0KBD4ZX, 0KBF0ZX, 0KBF3ZX, 0KBF4ZX, 0KBG0ZX, 0KBG3ZX, 0KBG4ZX, 0KBH0ZX, 0KBH3ZX, 0KBH4ZX, 0KBJ0ZX, 0KBJ3ZX, 0KBJ4ZX, 0KBK0ZX, 0KBK3ZX, 0KBK4ZX, 0KBL0ZX, 0KBL3ZX, 0KBL4ZX, 0KBM0ZX, 0KBM3ZX, 0KBM4ZX, 0KBN0ZX, 0KBN3ZX, 0KBN4ZX, 0KBP0ZX, 0KBP3ZX, 0KBP4ZX, 0KBQ0ZX, 0KBQ3ZX, 0KBQ4ZX, 0KBR0ZX, 0KBR3ZX, 0KBR4ZX, 0KBS0ZX, 0KBS3ZX, 0KBS4ZX, 0KBT0ZX, 0KBT3ZX, 0KBT4ZX, 0KBV0ZX, 0KBV3ZX, 0KBV4ZX, 0KBW0ZX, 0KBW3ZX, 0KBW4ZX, 0KJX0ZZ, 0KJX4ZZ, 0KJY0ZZ, 0KJY4ZZ, 0L900ZX, 0L904ZX, 0L910ZX, 0L914ZX, 0L920ZX, 0L924ZX, 0L930ZX, 0L934ZX, 0L940ZX, 0L944ZX, 0L950ZX, 0L954ZX,

© 2023 Wang Y et al. *JAMA Network Open.*

		0L960ZX, 0L964ZX, 0L970ZX, 0L974ZX, 0L980ZX, 0L984ZX, 0L990ZX, 0L994ZX, 0L9B0ZX, 0L9B4ZX, 0L9C0ZX, 0L9C4ZX, 0L9D0ZX, 0L9D4ZX, 0L9F0ZX, 0L9F4ZX, 0L9G0ZX, 0L9G4ZX, 0L9H0ZX, 0L9H4ZX, 0L9J0ZX, 0L9J4ZX, 0L9K0ZX, 0L9K4ZX, 0L9L0ZX, 0L9L4ZX, 0L9M0ZX, 0L9M4ZX, 0L9N0ZX, 0L9N4ZX, 0L9P0ZX, 0L9P4ZX, 0L9Q0ZX, 0L9Q4ZX, 0L9R0ZX, 0L9R4ZX, 0L9S0ZX, 0L9S4ZX, 0L9T0ZX, 0L9T4ZX, 0L9V0ZX, 0L9V4ZX, 0L9W0ZX, 0L9W4ZX, 0LB00ZX, 0LB03ZX, 0LB04ZX, 0LB10ZX, 0LB13ZX, 0LB14ZX, 0LB20ZX, 0LB23ZX, 0LB24ZX, 0LB30ZX, 0LB33ZX, 0LB34ZX, 0LB40ZX, 0LB43ZX, 0LB44ZX, 0LB50ZX, 0LB53ZX, 0LB54ZX, 0LB60ZX, 0LB63ZX, 0LB64ZX, 0LB70ZX, 0LB73ZX, 0LB74ZX, 0LB80ZX, 0LB83ZX, 0LB84ZX, 0LB90ZX, 0LB93ZX, 0LB94ZX, 0LBB0ZX, 0LBB3ZX, 0LBB4ZX, 0LBC0ZX, 0LBC3ZX, 0LBC4ZX, 0LBD0ZX, 0LBD3ZX, 0LBD4ZX, 0LBF0ZX, 0LBF3ZX, 0LBF4ZX, 0LBG0ZX, 0LBG3ZX, 0LBG4ZX, 0LBH0ZX, 0LBH3ZX, 0LBH4ZX, 0LBJ0ZX, 0LBJ3ZX, 0LBJ4ZX, 0LBK0ZX, 0LBK3ZX, 0LBK4ZX, 0LBL0ZX, 0LBL3ZX, 0LBL4ZX, 0LBM0ZX, 0LBM3ZX, 0LBM4ZX, 0LBN0ZX, 0LBN3ZX, 0LBN4ZX, 0LBP0ZX, 0LBP3ZX, 0LBP4ZX, 0LBQ0ZX, 0LBQ3ZX, 0LBQ4ZX, 0LBR0ZX, 0LBR3ZX, 0LBR4ZX, 0LBS0ZX, 0LBS3ZX, 0LBS4ZX, 0LBT0ZX, 0LBT3ZX, 0LBT4ZX, 0LBV0ZX, 0LBV3ZX, 0LBV4ZX, 0LBW0ZX, 0LBW3ZX, 0LBW4ZX, 0LJX0ZZ, 0LJX4ZZ, 0LJY0ZZ, 0LJY4ZZ, 0M904ZX, 0M914ZX, 0M924ZX, 0M934ZX, 0M944ZX, 0M954ZX, 0M964ZX, 0M974ZX, 0M984ZX, 0M990ZX, 0M994ZX, 0M9B0ZX, 0M9B4ZX, 0M9C4ZX, 0M9D4ZX, 0M9F4ZX, 0M9G4ZX, 0M9H0ZX, 0M9H4ZX, 0M9J0ZX, 0M9J4ZX, 0M9K0ZX, 0M9K4ZX, 0M9L4ZX, 0M9M4ZX, 0M9N4ZX, 0M9P4ZX, 0M9Q4ZX, 0M9R4ZX, 0M9S4ZX, 0M9T4ZX, 0M9V0ZX, 0M9V4ZX, 0M9W0ZX, 0M9W4ZX, 0MB04ZX, 0MB14ZX, 0MB24ZX, 0MB34ZX, 0MB44ZX, 0MB54ZX, 0MB64ZX, 0MB74ZX, 0MB84ZX, 0MB90ZX, 0MB93ZX, 0MB94ZX, 0MBB4ZX, 0MBC4ZX, 0MBD4ZX, 0MBF4ZX, 0MBG4ZX, 0MBH0ZX, 0MBH3ZX, 0MBH4ZX, 0MBJ0ZX, 0MBJ3ZX, 0MBJ4ZX, 0MBK0ZX, 0MBK3ZX, 0MBK4ZX, 0MBL4ZX, 0MBM4ZX, 0MBN4ZX, 0MBP4ZX, 0MBQ4ZX, 0MBR4ZX, 0MBS4ZX, 0MBT4ZX, 0MBV0ZX, 0MBV3ZX, 0MBV4ZX, 0MBW0ZX, 0MBW3ZX, 0MBW4ZX, 0MJX0ZZ, 0MJX4ZZ, 0MJY0ZZ, 0MJY4ZZ, 0N900ZX, 0N904ZX, 0N910ZX, 0N914ZX, 0N920ZX, 0N924ZX, 0N930ZX, 0N934ZX, 0N940ZX, 0N944ZX, 0N950ZX, 0N954ZX, 0N960ZX, 0N964ZX, 0N970ZX, 0N974ZX, 0N980ZX, 0N984ZX, 0N9B4ZX, 0N9C0ZX, 0N9C4ZX, 0N9D0ZX, 0N9D4ZX, 0N9F0ZX, 0N9F4ZX, 0N9G0ZX, 0N9G4ZX, 0N9H0ZX, 0N9H4ZX, 0N9J0ZX, 0N9J4ZX, 0N9K0ZX, 0N9K4ZX, 0N9L0ZX, 0N9L4ZX, 0N9M0ZX, 0N9M4ZX, 0N9N0ZX, 0N9N4ZX, 0N9P0ZX, 0N9P4ZX, 0N9Q0ZX, 0N9Q4ZX, 0N9R0ZX, 0N9R4ZX, 0N9S0ZX, 0N9S4ZX, 0N9T0ZX, 0N9T4ZX, 0N9V0ZX, 0N9V4ZX, 0N9X0ZX, 0N9X4ZX, 0NB00ZX, 0NB03ZX, 0NB04ZX, 0NB10ZX, 0NB13ZX, 0NB14ZX, 0NB20ZX, 0NB23ZX, 0NB24ZX, 0NB30ZX, 0NB33ZX, 0NB34ZX, 0NB40ZX, 0NB43ZX, 0NB44ZX, 0NB50ZX, 0NB53ZX, 0NB54ZX, 0NB60ZX, 0NB63ZX, 0NB64ZX, 0NB70ZX, 0NB73ZX, 0NB74ZX, 0NB80ZX,

© 2023 Wang Y et al. *JAMA Network Open.*

		0NB83ZX, 0NB84ZX, 0NBB4ZX, 0NBC0ZX, 0NBC3ZX, 0NBC4ZX, 0NBD0ZX, 0NBD3ZX, 0NBD4ZX, 0NBF0ZX, 0NBF3ZX, 0NBF4ZX, 0NBG0ZX, 0NBG3ZX, 0NBG4ZX, 0NBH0ZX, 0NBH3ZX, 0NBH4ZX, 0NBJ0ZX, 0NBJ3ZX, 0NBJ4ZX, 0NBK0ZX, 0NBK3ZX, 0NBK4ZX, 0NBL0ZX, 0NBL3ZX, 0NBL4ZX, 0NBM0ZX, 0NBM3ZX, 0NBM4ZX, 0NBN0ZX, 0NBN3ZX, 0NBN4ZX, 0NBP0ZX, 0NBP3ZX, 0NBP4ZX, 0NBQ0ZX, 0NBQ3ZX, 0NBQ4ZX, 0NBR4ZX, 0NBT4ZX, 0NBV4ZX, 0NBX0ZX, 0NBX3ZX, 0NBX4ZX, 0NJ00ZZ, 0NJ04ZZ, 0NJB0ZZ, 0NJB4ZZ, 0NJW0ZZ, 0NJW4ZZ, 0P900ZX, 0P904ZX, 0P910ZX, 0P914ZX, 0P920ZX, 0P924ZX, 0P930ZX, 0P934ZX, 0P940ZX, 0P944ZX, 0P950ZX, 0P954ZX, 0P960ZX, 0P964ZX, 0P970ZX, 0P974ZX, 0P980ZX, 0P984ZX, 0P990ZX, 0P994ZX, 0P9B0ZX, 0P9B4ZX, 0P9C0ZX, 0P9C4ZX, 0P9D0ZX, 0P9D4ZX, 0P9F0ZX, 0P9F4ZX, 0P9G0ZX, 0P9G4ZX, 0P9H0ZX, 0P9H4ZX, 0P9J0ZX, 0P9J4ZX, 0P9K0ZX, 0P9K4ZX, 0P9L0ZX, 0P9L4ZX, 0P9M0ZX, 0P9M4ZX, 0P9N0ZX, 0P9N4ZX, 0P9P0ZX, 0P9P4ZX, 0P9Q0ZX, 0P9Q4ZX, 0P9R0ZX, 0P9R4ZX, 0P9S0ZX, 0P9S4ZX, 0P9T0ZX, 0P9T4ZX, 0P9V0ZX, 0P9V4ZX, 0PB00ZX, 0PB03ZX, 0PB04ZX, 0PB10ZX, 0PB13ZX, 0PB14ZX, 0PB20ZX, 0PB23ZX, 0PB24ZX, 0PB30ZX, 0PB33ZX, 0PB34ZX, 0PB40ZX, 0PB43ZX, 0PB44ZX, 0PB50ZX, 0PB53ZX, 0PB54ZX, 0PB60ZX, 0PB63ZX, 0PB64ZX, 0PB70ZX, 0PB73ZX, 0PB74ZX, 0PB80ZX, 0PB83ZX, 0PB84ZX, 0PB90ZX, 0PB93ZX, 0PB94ZX, 0PBB0ZX, 0PBB3ZX, 0PBB4ZX, 0PBC0ZX, 0PBC3ZX, 0PBC4ZX, 0PBD0ZX, 0PBD3ZX, 0PBD4ZX, 0PBF0ZX, 0PBF3ZX, 0PBF4ZX, 0PBG0ZX, 0PBG3ZX, 0PBG4ZX, 0PBH0ZX, 0PBH3ZX, 0PBH4ZX, 0PBJ0ZX, 0PBJ3ZX, 0PBJ4ZX, 0PBK0ZX, 0PBK3ZX, 0PBK4ZX, 0PBL0ZX, 0PBL3ZX, 0PBL4ZX, 0PBM0ZX, 0PBM3ZX, 0PBM4ZX, 0PBN0ZX, 0PBN3ZX, 0PBN4ZX, 0PBP0ZX, 0PBP3ZX, 0PBP4ZX, 0PBQ0ZX, 0PBQ3ZX, 0PBQ4ZX, 0PBR0ZX, 0PBR3ZX, 0PBR4ZX, 0PBS0ZX, 0PBS3ZX, 0PBS4ZX, 0PBT0ZX, 0PBT3ZX, 0PBT4ZX, 0PBV0ZX, 0PBV3ZX, 0PBV4ZX, 0PJY0ZZ, 0PJY4ZZ, 0Q900ZX, 0Q904ZX, 0Q910ZX, 0Q914ZX, 0Q920ZX, 0Q924ZX, 0Q930ZX, 0Q934ZX, 0Q940ZX, 0Q944ZX, 0Q950ZX, 0Q954ZX, 0Q960ZX, 0Q964ZX, 0Q970ZX, 0Q974ZX, 0Q980ZX, 0Q984ZX, 0Q990ZX, 0Q994ZX, 0Q9B0ZX, 0Q9B4ZX, 0Q9C0ZX, 0Q9C4ZX, 0Q9D0ZX, 0Q9D4ZX, 0Q9F0ZX, 0Q9F4ZX, 0Q9G0ZX, 0Q9G4ZX, 0Q9H0ZX, 0Q9H4ZX, 0Q9J0ZX, 0Q9J4ZX, 0Q9K0ZX, 0Q9K4ZX, 0Q9L0ZX, 0Q9L4ZX, 0Q9M0ZX, 0Q9M4ZX, 0Q9N0ZX, 0Q9N4ZX, 0Q9P0ZX, 0Q9P4ZX, 0Q9Q0ZX, 0Q9Q4ZX, 0Q9R0ZX, 0Q9R4ZX, 0Q9S0ZX, 0Q9S4ZX, 0QB00ZX, 0QB03ZX, 0QB04ZX, 0QB10ZX, 0QB13ZX, 0QB14ZX, 0QB20ZX, 0QB23ZX, 0QB24ZX, 0QB30ZX, 0QB33ZX, 0QB34ZX, 0QB40ZX, 0QB43ZX, 0QB44ZX, 0QB50ZX, 0QB53ZX, 0QB54ZX, 0QB60ZX, 0QB63ZX, 0QB64ZX, 0QB70ZX, 0QB73ZX, 0QB74ZX, 0QB80ZX, 0QB83ZX, 0QB84ZX, 0QB90ZX, 0QB93ZX, 0QB94ZX, 0QBB0ZX, 0QBB3ZX, 0QBB4ZX, 0QBC0ZX, 0QBC3ZX, 0QBC4ZX, 0QBD0ZX, 0QBD3ZX, 0QBD4ZX, 0QBF0ZX, 0QBF3ZX, 0QBF4ZX, 0QBG0ZX, 0QBG3ZX, 0QBG4ZX, 0QBH0ZX, 0QBH3ZX, 0QBH4ZX, 0QBJ0ZX, 0QBJ3ZX,

© 2023 Wang Y et al. *JAMA Network Open.*

		0QBJ4ZX, 0QBK0ZX, 0QBK3ZX, 0QBK4ZX, 0QBL0ZX, 0QBL3ZX, 0QBL4ZX, 0QBM0ZX, 0QBM3ZX, 0QBM4ZX, 0QBN0ZX, 0QBN3ZX, 0QBN4ZX, 0QBP0ZX, 0QBP3ZX, 0QBP4ZX, 0QBQ0ZX, 0QBQ3ZX, 0QBQ4ZX, 0QBR0ZX, 0QBR3ZX, 0QBR4ZX, 0QBS0ZX, 0QBS3ZX, 0QBS4ZX, 0QJY0ZZ, 0QJY4ZZ, 0R904ZX, 0R914ZX, 0R934ZX, 0R944ZX, 0R954ZX, 0R964ZX, 0R994ZX, 0R9A4ZX, 0R9B4ZX, 0R9C0ZX, 0R9C4ZX, 0R9D0ZX, 0R9D4ZX, 0R9E4ZX, 0R9F4ZX, 0R9G4ZX, 0R9H4ZX, 0R9J4ZX, 0R9K4ZX, 0R9L4ZX, 0R9M4ZX, 0R9N4ZX, 0R9P4ZX, 0R9Q4ZX, 0R9R4ZX, 0R9S4ZX, 0R9T4ZX, 0R9U4ZX, 0R9V4ZX, 0R9W4ZX, 0R9X4ZX, 0RB04ZX, 0RB14ZX, 0RB34ZX, 0RB44ZX, 0RB54ZX, 0RB64ZX, 0RB94ZX, 0RBA4ZX, 0RBB4ZX, 0RBC0ZX, 0RBC3ZX, 0RBC4ZX, 0RBD0ZX, 0RBD3ZX, 0RBD4ZX, 0RBE4ZX, 0RBF4ZX, 0RBG4ZX, 0RBH4ZX, 0RBJ4ZX, 0RBK4ZX, 0RBL4ZX, 0RBM4ZX, 0RBN4ZX, 0RBP4ZX, 0RBQ4ZX, 0RBR4ZX, 0RBS4ZX, 0RBT4ZX, 0RBU4ZX, 0RBV4ZX, 0RBW4ZX, 0RBX4ZX, 0RJ00ZZ, 0RJ04ZZ, 0RJ10ZZ, 0RJ14ZZ, 0RJ30ZZ, 0RJ34ZZ, 0RJ40ZZ, 0RJ44ZZ, 0RJ50ZZ, 0RJ54ZZ, 0RJ60ZZ, 0RJ64ZZ, 0RJ90ZZ, 0RJ94ZZ, 0RJA0ZZ, 0RJA4ZZ, 0RJB0ZZ, 0RJB4ZZ, 0RJC0ZZ, 0RJC4ZZ, 0RJD0ZZ, 0RJD4ZZ, 0RJE0ZZ, 0RJE4ZZ, 0RJF0ZZ, 0RJF4ZZ, 0RJG0ZZ, 0RJG4ZZ, 0RJH0ZZ, 0RJH4ZZ, 0RJJ0ZZ, 0RJJ4ZZ, 0RJK0ZZ, 0RJK4ZZ, 0RJL0ZZ, 0RJL4ZZ, 0RJM0ZZ, 0RJM4ZZ, 0RJN0ZZ, 0RJN4ZZ, 0RJP0ZZ, 0RJP4ZZ, 0RJQ0ZZ, 0RJQ4ZZ, 0RJR0ZZ, 0RJR4ZZ, 0RJS0ZZ, 0RJS4ZZ, 0RJT0ZZ, 0RJT4ZZ, 0RJU0ZZ, 0RJU4ZZ, 0RJV0ZZ, 0RJV4ZZ, 0RJW0ZZ, 0RJW4ZZ, 0RJX0ZZ, 0RJX4ZZ, 0S904ZX, 0S924ZX, 0S934ZX, 0S944ZX, 0S954ZX, 0S964ZX, 0S974ZX, 0S984ZX, 0S994ZX, 0S9B4ZX, 0S9C4ZX, 0S9D4ZX, 0S9F4ZX, 0S9G4ZX, 0S9H4ZX, 0S9J4ZX, 0S9K4ZX, 0S9L4ZX, 0S9M4ZX, 0S9N4ZX, 0S9P4ZX, 0S9Q4ZX, 0SB04ZX, 0SB24ZX, 0SB34ZX, 0SB44ZX, 0SB54ZX, 0SB64ZX, 0SB74ZX, 0SB84ZX, 0SB94ZX, 0SBB4ZX, 0SBC4ZX, 0SBD4ZX, 0SBF4ZX, 0SBG4ZX, 0SBH4ZX, 0SBJ4ZX, 0SBK4ZX, 0SBL4ZX, 0SBM4ZX, 0SBN4ZX, 0SBP4ZX, 0SBQ4ZX, 0SJ00ZZ, 0SJ04ZZ, 0SJ20ZZ, 0SJ24ZZ, 0SJ30ZZ, 0SJ34ZZ, 0SJ40ZZ, 0SJ44ZZ, 0SJ50ZZ, 0SJ54ZZ, 0SJ60ZZ, 0SJ64ZZ, 0SJ70ZZ, 0SJ74ZZ, 0SJ80ZZ, 0SJ84ZZ, 0SJ90ZZ, 0SJ94ZZ, 0SJB0ZZ, 0SJB4ZZ, 0SJC0ZZ, 0SJC4ZZ, 0SJD0ZZ, 0SJD4ZZ, 0SJF0ZZ, 0SJF4ZZ, 0SJG0ZZ, 0SJG4ZZ, 0SJH0ZZ, 0SJH4ZZ, 0SJJ0ZZ, 0SJJ4ZZ, 0SJK0ZZ, 0SJK4ZZ, 0SJL0ZZ, 0SJL4ZZ, 0SJM0ZZ, 0SJM4ZZ, 0SJN0ZZ, 0SJN4ZZ, 0SJP0ZZ, 0SJP4ZZ, 0SJQ0ZZ, 0SJQ4ZZ, 0T900ZX, 0T904ZX, 0T910ZX, 0T914ZX, 0T930ZX, 0T934ZX, 0T940ZX, 0T944ZX, 0T960ZX, 0T964ZX, 0T970ZX, 0T974ZX, 0T980ZX, 0T984ZX, 0T9B0ZX, 0T9B4ZX, 0T9B7ZX, 0T9B8ZX, 0T9C0ZX, 0T9C4ZX, 0T9C7ZX, 0T9C8ZX, 0T9D4ZX, 0TB00ZX, 0TB04ZX, 0TB10ZX, 0TB14ZX, 0TB30ZX, 0TB34ZX, 0TB40ZX, 0TB44ZX, 0TB60ZX, 0TB64ZX, 0TB70ZX, 0TB74ZX, 0TBB0ZX, 0TBB3ZX, 0TBB4ZX, 0TBB7ZX, 0TBB8ZX, 0TBC0ZX, 0TBC3ZX, 0TBC4ZX, 0TBC7ZX, 0TBC8ZX, 0TBD4ZX, 0TH502Z, 0TH532Z, 0TH542Z, 0TH902Z, 0TH932Z, 0TH942Z,

© 2023 Wang Y et al. *JAMA Network Open.*

		0THB02Z, 0THB32Z, 0THB42Z, 0THD02Z, 0THD32Z, 0THD42Z, 0THDX2Z, 0TJ50ZZ, 0TJ54ZZ, 0TJ90ZZ, 0TJ94ZZ, 0TJB0ZZ, 0TJB4ZZ, 0TJD0ZZ, 0TJD4ZZ, 0U900ZX, 0U904ZX, 0U908ZX, 0U910ZX, 0U914ZX, 0U918ZX, 0U920ZX, 0U924ZX, 0U928ZX, 0U940ZX, 0U944ZX, 0U948ZX, 0U950ZX, 0U954ZX, 0U957ZX, 0U958ZX, 0U960ZX, 0U964ZX, 0U967ZX, 0U968ZX, 0U970ZX, 0U974ZX, 0U977ZX, 0U978ZX, 0U990ZX, 0U994ZX, 0U997ZX, 0U998ZX, 0U9C0ZX, 0U9C4ZX, 0U9C7ZX, 0U9C8ZX, 0U9F0ZX, 0U9F4ZX, 0U9F7ZX, 0U9F8ZX, 0U9G0ZX, 0U9G4ZX, 0U9G7ZX, 0U9G8ZX, 0U9GXZX, 0U9J0ZX, 0U9JXZX, 0U9K0ZX, 0U9K4ZX, 0U9K7ZX, 0U9K8ZX, 0U9KXZX, 0U9L0ZX, 0U9LXZX, 0U9M0ZX, 0U9MXZX, 0UB00ZX, 0UB03ZX, 0UB04ZX, 0UB07ZX, 0UB08ZX, 0UB10ZX, 0UB13ZX, 0UB14ZX, 0UB17ZX, 0UB18ZX, 0UB20ZX, 0UB23ZX, 0UB24ZX, 0UB27ZX, 0UB28ZX, 0UB40ZX, 0UB43ZX, 0UB44ZX, 0UB47ZX, 0UB48ZX, 0UB50ZX, 0UB53ZX, 0UB54ZX, 0UB57ZX, 0UB58ZX, 0UB60ZX, 0UB63ZX, 0UB64ZX, 0UB67ZX, 0UB68ZX, 0UB70ZX, 0UB73ZX, 0UB74ZX, 0UB77ZX, 0UB78ZX, 0UB90ZX, 0UB93ZX, 0UB94ZX, 0UB97ZX, 0UB98ZX, 0UBC0ZX, 0UBC3ZX, 0UBC4ZX, 0UBC7ZX, 0UBC8ZX, 0UBF0ZX, 0UBF3ZX, 0UBF4ZX, 0UBF7ZX, 0UBF8ZX, 0UBG0ZX, 0UBG3ZX, 0UBG4ZX, 0UBG7ZX, 0UBG8ZX, 0UBGXZX, 0UBJ0ZX, 0UBJXZX, 0UBK0ZX, 0UBK3ZX, 0UBK4ZX, 0UBK7ZX, 0UBK8ZX, 0UBKXZX, 0UBL0ZX, 0UBLXZX, 0UBM0ZX, 0UBMXZX, 0UDB7ZX, 0UDB8ZX, 0UJ30ZZ, 0UJ34ZZ, 0UJ80ZZ, 0UJ84ZZ, 0UJD0ZZ, 0UJD4ZZ, 0UJH0ZZ, 0UJH4ZZ, 0UJM0ZZ, 0V900ZX, 0V904ZX, 0V910ZX, 0V914ZX, 0V920ZX, 0V924ZX, 0V930ZX, 0V934ZX, 0V954ZX, 0V964ZX, 0V974ZX, 0V990ZX, 0V994ZX, 0V9B0ZX, 0V9B4ZX, 0V9C0ZX, 0V9C4ZX, 0V9F4ZX, 0V9G4ZX, 0V9H4ZX, 0V9J4ZX, 0V9K4ZX, 0V9L4ZX, 0V9N4ZX, 0V9P4ZX, 0V9Q4ZX, 0V9S0ZX, 0V9S4ZX, 0V9SXZX, 0V9T0ZX, 0V9T4ZX, 0V9TXZX, 0VB00ZX, 0VB04ZX, 0VB10ZX, 0VB14ZX, 0VB20ZX, 0VB24ZX, 0VB30ZX, 0VB34ZX, 0VB54ZX, 0VB64ZX, 0VB74ZX, 0VB90ZX, 0VB94ZX, 0VBB0ZX, 0VBB4ZX, 0VBC0ZX, 0VBC4ZX, 0VBF4ZX, 0VBG4ZX, 0VBH4ZX, 0VBJ4ZX, 0VBK4ZX, 0VBL4ZX, 0VBN4ZX, 0VBP4ZX, 0VBQ4ZX, 0VBS0ZX, 0VBS3ZX, 0VBS4ZX, 0VBSXZX, 0VBT0ZX, 0VBT3ZX, 0VBT4ZX, 0VBTXZX, 0VJ40ZZ, 0VJ44ZZ, 0VJ84ZZ, 0VJD0ZZ, 0VJD4ZZ, 0VJM0ZZ, 0VJM4ZZ, 0VJR0ZZ, 0VJR4ZZ, 0VJS0ZZ, 0VJS4ZZ, 0W904ZX, 0W910ZX, 0W914ZX, 0W924ZX, 0W934ZX, 0W944ZX, 0W954ZX, 0W964ZX, 0W984ZX, 0W994ZX, 0W9B4ZX, 0W9C0ZX, 0W9C4ZX, 0W9D0ZX, 0W9D4ZX, 0W9F0ZX, 0W9F4ZX, 0W9G0ZX, 0W9G4ZX, 0W9H0ZX, 0W9H4ZX, 0W9J0ZX, 0W9J4ZX, 0W9K4ZX, 0W9L4ZX, 0W9M4ZX, 0W9N4ZX, 0WB04ZX, 0WB24ZX, 0WB30ZX, 0WB34ZX, 0WB44ZX, 0WB54ZX, 0WB64ZX, 0WB84ZX, 0WBC0ZX, 0WBC4ZX, 0WBF0ZX, 0WBF3ZX, 0WBF4ZX, 0WBFXZX, 0WBH0ZX, 0WBH4ZX, 0WBK4ZX, 0WBL4ZX, 0WBM4ZX, 0WBN0ZX, 0WBN3ZX, 0WBN4ZX, 0WBNXZX, 0WJ04ZZ, 0WJ10ZZ, 0WJ14ZZ, 0WJ24ZZ, 0WJ34ZZ, 0WJ44ZZ, 0WJ54ZZ, 0WJ60ZZ, 0WJ64ZZ, 0WJ80ZZ, 0WJ84ZZ, 0WJ90ZZ, 0WJ94ZZ, 0WJB0ZZ,

© 2023 Wang Y et al. *JAMA Network Open.*

		0WJB4ZZ, 0WJC0ZZ, 0WJC4ZZ, 0WJD4ZZ, 0WJF0ZZ, 0WJF4ZZ, 0WJG0ZZ, 0WJG4ZZ, 0WJH0ZZ, 0WJH4ZZ, 0WJJ0ZZ, 0WJJ4ZZ, 0WJK4ZZ, 0WJL4ZZ, 0WJM4ZZ, 0WJN0ZZ, 0WJN4ZZ, 0WJP0ZZ, 0WJP4ZZ, 0WJQ0ZZ, 0WJQ4ZZ, 0WJR0ZZ, 0WJR4ZZ, 0X924ZX, 0X934ZX, 0X944ZX, 0X954ZX, 0X964ZX, 0X974ZX, 0X984ZX, 0X994ZX, 0X9B4ZX, 0X9C4ZX, 0X9D4ZX, 0X9F4ZX, 0X9G4ZX, 0X9H4ZX, 0X9J4ZX, 0X9K4ZX, 0XB24ZX, 0XB34ZX, 0XB44ZX, 0XB54ZX, 0XB64ZX, 0XB74ZX, 0XB84ZX, 0XB94ZX, 0XBB4ZX, 0XBC4ZX, 0XBD4Z
	**HCPC**	G0342, G0343, G0412, G0413, G0414, G0415, 11450, 11451, 11462, 11463, 11470, 11471, 11771, 11772, 11960, 11970, 11971, 13160, 14000, 14001, 14020, 14021, 14040, 14041, 14060, 14061, 14301, 14350, 15050, 15100, 15110, 15115, 15120, 15130, 15135, 15150, 15155, 15200, 15220, 15240, 15260, 15570, 15572, 15574, 15576, 15600, 15610, 15620, 15630, 15650, 15730, 15731, 15733, 15734, 15736, 15738, 15740, 15750, 15756, 15757, 15758, 15760, 15769, 15770, 15771, 15773, 15780, 15781, 15782, 15783, 15788, 15789, 15792, 15793, 15819, 15820, 15821, 15822, 15823, 15830, 15832, 15833, 15834, 15835, 15836, 15837, 15838, 15839, 15840, 15841, 15842, 15845, 15920, 15922, 15931, 15933, 15934, 15935, 15936, 15937, 15940, 15941, 15944, 15945, 15946, 15950, 15951, 15952, 15953, 15956, 15958, 17106, 17107, 17108, 19020, 19110, 19112, 19120, 19125, 19300, 19301, 19302, 19303, 19305, 19306, 19307, 19316, 19318, 19325, 19328, 19330, 19340, 19342, 19350, 19355, 19357, 19361, 19364, 19367, 19368, 19369, 19370, 19371, 19380, 20150, 20661, 20662, 20663, 20664, 20680, 20690, 20692, 20693, 20694, 20696, 20802, 20805, 20808, 20816, 20822, 20824, 20827, 20838, 20910, 20912, 20920, 20922, 20924, 20955, 20956, 20957, 20962, 20969, 20970, 20972, 20973, 21010, 21011, 21012, 21013, 21014, 21015, 21016, 21025, 21026, 21029, 21030, 21031, 21032, 21034, 21040, 21044, 21045, 21046, 21047, 21048, 21049, 21050, 21060, 21070, 21073, 21077, 21079, 21080, 21081, 21082, 21083, 21084, 21086, 21087, 21088, 21100, 21110, 21120, 21121, 21122, 21123, 21125, 21127, 21137, 21138, 21139, 21141, 21142, 21143, 21145, 21146, 21147, 21150, 21151, 21154, 21155, 21159, 21160, 21172, 21175, 21179, 21180, 21181, 21182, 21183, 21184, 21188, 21193, 21194, 21195, 21196, 21198, 21199, 21206, 21208, 21209, 21210, 21215, 21230, 21235, 21240, 21242, 21243, 21244, 21245, 21246, 21247, 21248, 21249, 21255, 21256, 21260, 21261, 21263, 21267, 21268, 21270, 21275, 21280, 21282, 21295, 21296, 21325, 21330, 21335, 21336, 21337, 21338, 21339, 21340, 21343, 21344, 21345, 21346, 21347, 21348, 21360, 21365, 21366, 21385, 21386, 21387, 21390, 21395, 21400, 21401, 21406, 21407, 21408, 21421, 21422, 21423, 21431, 21432, 21433, 21435, 21436, 21440, 21445, 21450, 21451, 21452, 21453, 21454, 21461, 21462, 21465, 21470, 21485, 21490, 21497, 21501, 21502, 21510, 21552, 21554, 21555, 21556, 21557, 21558, 21600, 21601, 21602, 21603, 21610, 21615, 21616, 21620, 21627, 21630, 21632, 21685, 21700, 21705, 21720, 21725, 21740, 21742, 21743, 21750, 21820, 21825, 21925, 21930, 21931, 21932, 21933, 21935,

© 2023 Wang Y et al. *JAMA Network Open.*

		21936, 22010, 22015, 22100, 22101, 22102, 22110, 22112, 22114, 22206, 22207, 22210, 22212, 22214, 22220, 22222, 22224, 22310, 22315, 22318, 22319, 22325, 22326, 22327, 22532, 22533, 22548, 22551, 22554, 22556, 22558, 22586, 22590, 22595, 22600, 22610, 22612, 22630, 22633, 22800, 22802, 22804, 22808, 22810, 22812, 22818, 22819, 22830, 22849, 22850, 22852, 22855, 22856, 22857, 22861, 22862, 22864, 22865, 22867, 22869, 22900, 22901, 22902, 22903, 22904, 22905, 23000, 23020, 23035, 23040, 23044, 23066, 23071, 23073, 23075, 23076, 23077, 23078, 23100, 23101, 23105, 23106, 23107, 23120, 23125, 23130, 23140, 23145, 23146, 23150, 23155, 23156, 23170, 23172, 23174, 23180, 23182, 23184, 23190, 23195, 23200, 23210, 23220, 23333, 23334, 23335, 23395, 23397, 23400, 23405, 23406, 23410, 23412, 23415, 23420, 23430, 23440, 23450, 23455, 23460, 23462, 23465, 23466, 23470, 23472, 23473, 23474, 23480, 23485, 23490, 23491, 23500, 23505, 23515, 23520, 23525, 23530, 23532, 23540, 23545, 23550, 23552, 23570, 23575, 23585, 23600, 23605, 23615, 23616, 23620, 23625, 23630, 23650, 23655, 23660, 23665, 23670, 23675, 23680, 23800, 23802, 23900, 23920, 23921, 23935, 24000, 24006, 24066, 24071, 24073, 24075, 24076, 24077, 24079, 24100, 24101, 24102, 24105, 24110, 24115, 24116, 24120, 24125, 24126, 24130, 24134, 24136, 24138, 24140, 24145, 24147, 24149, 24150, 24152, 24155, 24160, 24164, 24201, 24300, 24301, 24305, 24310, 24320, 24330, 24331, 24332, 24340, 24341, 24342, 24343, 24344, 24345, 24346, 24357, 24358, 24359, 24360, 24361, 24362, 24363, 24365, 24366, 24370, 24371, 24400, 24410, 24420, 24430, 24435, 24470, 24495, 24498, 24500, 24505, 24515, 24516, 24530, 24535, 24538, 24545, 24546, 24560, 24565, 24566, 24575, 24576, 24577, 24579, 24582, 24586, 24587, 24600, 24605, 24615, 24620, 24635, 24650, 24655, 24665, 24666, 24670, 24675, 24685, 24800, 24802, 24900, 24920, 24925, 24930, 24931, 24935, 24940, 25000, 25001, 25020, 25023, 25024, 25025, 25028, 25031, 25035, 25040, 25066, 25071, 25073, 25075, 25076, 25077, 25078, 25085, 25100, 25101, 25105, 25107, 25109, 25110, 25111, 25112, 25115, 25116, 25118, 25119, 25120, 25125, 25126, 25130, 25135, 25136, 25145, 25150, 25151, 25170, 25210, 25215, 25230, 25240, 25248, 25250, 25251, 25259, 25260, 25263, 25265, 25270, 25272, 25274, 25275, 25280, 25290, 25295, 25300, 25301, 25310, 25312, 25315, 25316, 25320, 25332, 25335, 25337, 25350, 25355, 25360, 25365, 25370, 25375, 25390, 25391, 25392, 25393, 25394, 25400, 25405, 25415, 25420, 25425, 25426, 25430, 25431, 25440, 25441, 25442, 25443, 25444, 25445, 25446, 25447, 25449, 25450, 25455, 25490, 25491, 25492, 25500, 25505, 25515, 25520, 25525, 25526, 25530, 25535, 25545, 25560, 25565, 25574, 25575, 25600, 25605, 25606, 25607, 25608, 25609, 25622, 25624, 25628, 25630, 25635, 25645, 25650, 25651, 25652, 25660, 25670, 25671, 25675, 25676, 25680, 25685, 25690, 25695, 25800, 25805, 25810, 25820, 25825, 25830, 25900, 25905, 25907, 25909, 25915, 25920, 25922, 25924, 25927, 25929, 25931, 26020, 26025, 26030, 26034, 26035, 26037, 26040, 26045, 26055, 26060, 26070, 26075, 26080, 26100, 26105, 26110, 26111, 26113, 26115, 26116, 26117, 26118, 26121, 26123, 26130, 26135, 26140, 26145, 26160, 26170, 26180, 26185, 26200, 26205, 26210, 26215, 26230, 26235, 26236, 26250, 26260,

© 2023 Wang Y et al. *JAMA Network Open.*

		26262, 26320, 26340, 26350, 26352, 26356, 26357, 26358, 26370, 26372, 26373, 26390, 26392, 26410, 26412, 26415, 26416, 26418, 26420, 26426, 26428, 26432, 26433, 26434, 26437, 26440, 26442, 26445, 26449, 26450, 26455, 26460, 26471, 26474, 26476, 26477, 26478, 26479, 26480, 26483, 26485, 26489, 26490, 26492, 26494, 26496, 26497, 26498, 26499, 26500, 26502, 26508, 26510, 26516, 26517, 26518, 26520, 26525, 26530, 26531, 26535, 26536, 26540, 26541, 26542, 26545, 26546, 26548, 26550, 26551, 26553, 26554, 26555, 26556, 26560, 26561, 26562, 26565, 26567, 26568, 26580, 26587, 26590, 26591, 26593, 26596, 26600, 26605, 26607, 26608, 26615, 26641, 26645, 26650, 26665, 26670, 26675, 26676, 26685, 26686, 26700, 26705, 26706, 26715, 26720, 26725, 26727, 26735, 26740, 26742, 26746, 26750, 26755, 26756, 26765, 26770, 26775, 26776, 26785, 26820, 26841, 26842, 26843, 26844, 26850, 26852, 26860, 26862, 26910, 26951, 26952, 26990, 26991, 26992, 27000, 27001, 27003, 27005, 27006, 27025, 27027, 27030, 27033, 27035, 27036, 27041, 27043, 27045, 27047, 27048, 27049, 27050, 27052, 27054, 27057, 27059, 27060, 27062, 27065, 27066, 27067, 27070, 27071, 27075, 27076, 27077, 27078, 27080, 27087, 27090, 27091, 27097, 27098, 27100, 27105, 27110, 27111, 27120, 27122, 27125, 27130, 27132, 27134, 27137, 27138, 27140, 27146, 27147, 27151, 27156, 27158, 27161, 27165, 27170, 27175, 27176, 27177, 27178, 27179, 27181, 27185, 27187, 27200, 27202, 27215, 27216, 27217, 27218, 27220, 27222, 27226, 27227, 27228, 27230, 27232, 27235, 27236, 27238, 27240, 27244, 27245, 27246, 27248, 27252, 27253, 27254, 27258, 27259, 27265, 27266, 27267, 27268, 27269, 27279, 27280, 27282, 27284, 27286, 27290, 27295, 27301, 27303, 27305, 27306, 27307, 27310, 27324, 27325, 27326, 27327, 27328, 27329, 27330, 27331, 27332, 27333, 27334, 27335, 27337, 27339, 27340, 27345, 27347, 27350, 27355, 27356, 27357, 27360, 27364, 27365, 27372, 27380, 27381, 27385, 27386, 27390, 27391, 27392, 27393, 27394, 27395, 27396, 27397, 27400, 27403, 27405, 27407, 27409, 27412, 27415, 27416, 27418, 27420, 27422, 27424, 27425, 27427, 27428, 27429, 27430, 27435, 27437, 27438, 27440, 27441, 27442, 27443, 27445, 27446, 27447, 27448, 27450, 27454, 27455, 27457, 27465, 27466, 27468, 27470, 27472, 27475, 27477, 27479, 27485, 27486, 27487, 27488, 27495, 27496, 27497, 27498, 27499, 27500, 27501, 27502, 27503, 27506, 27507, 27508, 27509, 27510, 27511, 27513, 27514, 27516, 27517, 27519, 27520, 27524, 27530, 27532, 27535, 27536, 27538, 27540, 27550, 27552, 27556, 27557, 27558, 27560, 27562, 27566, 27580, 27590, 27591, 27592, 27594, 27596, 27598, 27600, 27601, 27602, 27603, 27604, 27607, 27610, 27612, 27614, 27615, 27616, 27618, 27619, 27620, 27625, 27626, 27630, 27632, 27634, 27635, 27637, 27638, 27640, 27641, 27645, 27646, 27647, 27650, 27652, 27654, 27656, 27658, 27659, 27664, 27665, 27675, 27676, 27680, 27681, 27685, 27686, 27687, 27690, 27691, 27695, 27696, 27698, 27700, 27702, 27703, 27704, 27705, 27707, 27709, 27712, 27715, 27720, 27722, 27724, 27725, 27726, 27727, 27730, 27732, 27734, 27740, 27742, 27745, 27750, 27752, 27756, 27758, 27759, 27760, 27762, 27766, 27767, 27768, 27769, 27780, 27781, 27784, 27786, 27788, 27792, 27808, 27810, 27814, 27816, 27818, 27822, 27823, 27824, 27825, 27826, 27827, 27828, 27829,

© 2023 Wang Y et al. *JAMA Network Open.*

		27830, 27831, 27832, 27840, 27842, 27846, 27848, 27870, 27871, 27880, 27881, 27882, 27884, 27886, 27888, 27889, 27892, 27893, 27894, 28003, 28005, 28008, 28010, 28011, 28020, 28022, 28024, 28035, 28039, 28041, 28043, 28045, 28046, 28047, 28050, 28052, 28054, 28055, 28060, 28062, 28070, 28072, 28080, 28086, 28088, 28090, 28092, 28100, 28102, 28103, 28104, 28106, 28107, 28108, 28110, 28111, 28112, 28113, 28114, 28116, 28118, 28119, 28120, 28122, 28124, 28126, 28130, 28140, 28150, 28153, 28160, 28171, 28173, 28175, 28192, 28193, 28200, 28202, 28208, 28210, 28220, 28222, 28225, 28226, 28230, 28232, 28234, 28238, 28240, 28250, 28260, 28261, 28262, 28264, 28270, 28272, 28280, 28285, 28286, 28288, 28289, 28291, 28292, 28295, 28296, 28297, 28298, 28299, 28300, 28302, 28304, 28305, 28306, 28307, 28308, 28309, 28310, 28312, 28313, 28315, 28320, 28322, 28340, 28341, 28344, 28345, 28360, 28400, 28405, 28406, 28415, 28420, 28430, 28435, 28436, 28445, 28446, 28450, 28455, 28456, 28465, 28470, 28475, 28476, 28485, 28490, 28495, 28496, 28505, 28510, 28515, 28525, 28530, 28531, 28540, 28545, 28546, 28555, 28570, 28575, 28576, 28585, 28600, 28605, 28606, 28615, 28645, 28675, 28705, 28715, 28725, 28730, 28735, 28737, 28740, 28750, 28755, 28760, 28800, 28805, 28810, 28890, 29800, 29804, 29805, 29806, 29807, 29819, 29820, 29821, 29822, 29823, 29824, 29825, 29827, 29828, 29830, 29834, 29835, 29836, 29837, 29838, 29840, 29843, 29844, 29845, 29846, 29847, 29848, 29850, 29851, 29855, 29856, 29860, 29861, 29862, 29863, 29866, 29867, 29868, 29870, 29871, 29873, 29874, 29875, 29876, 29877, 29879, 29880, 29881, 29882, 29883, 29884, 29885, 29886, 29887, 29888, 29889, 29891, 29892, 29893, 29894, 29895, 29897, 29898, 29899, 29900, 29901, 29902, 29904, 29905, 29906, 29907, 29914, 29915, 29916, 30115, 30117, 30118, 30120, 30124, 30125, 30130, 30150, 30160, 30320, 30400, 30410, 30420, 30430, 30435, 30450, 30460, 30462, 30465, 30520, 30540, 30545, 30580, 30600, 30620, 30630, 30915, 30920, 31020, 31030, 31032, 31040, 31050, 31051, 31070, 31075, 31080, 31081, 31084, 31085, 31086, 31087, 31090, 31200, 31201, 31205, 31225, 31230, 31300, 31360, 31365, 31367, 31368, 31370, 31375, 31380, 31382, 31390, 31395, 31400, 31420, 31551, 31552, 31553, 31554, 31580, 31584, 31587, 31590, 31591, 31592, 31610, 31611, 31613, 31614, 31750, 31755, 31760, 31766, 31770, 31775, 31780, 31781, 31785, 31786, 31800, 31805, 31820, 31825, 31830, 32035, 32036, 32096, 32097, 32098, 32100, 32110, 32120, 32124, 32140, 32141, 32150, 32151, 32160, 32200, 32215, 32220, 32225, 32310, 32320, 32440, 32442, 32445, 32480, 32482, 32484, 32486, 32488, 32491, 32503, 32504, 32505, 32540, 32650, 32651, 32652, 32653, 32654, 32655, 32656, 32658, 32659, 32661, 32662, 32663, 32664, 32665, 32666, 32669, 32670, 32671, 32672, 32673, 32800, 32810, 32815, 32820, 32851, 32852, 32853, 32854, 32900, 32905, 32906, 32940, 33020, 33025, 33030, 33031, 33050, 33120, 33130, 33140, 33202, 33203, 33206, 33207, 33208, 33212, 33213, 33214, 33215, 33216, 33217, 33218, 33220, 33221, 33222, 33223, 33227, 33228, 33229, 33230, 33231, 33233, 33234, 33235, 33236, 33237, 33238, 33240, 33241, 33243, 33244, 33249, 33250, 33251, 33254, 33255, 33256, 33261, 33262, 33263, 33264, 33265, 33266, 33270, 33271, 33272, 33273, 33274, 33275,

© 2023 Wang Y et al. *JAMA Network Open.*

		33300, 33305, 33310, 33315, 33320, 33321, 33322, 33330, 33335, 33390, 33391, 33404, 33405, 33406, 33410, 33411, 33412, 33413, 33414, 33415, 33416, 33417, 33418, 33420, 33422, 33425, 33426, 33427, 33430, 33440, 33460, 33463, 33464, 33465, 33468, 33470, 33471, 33474, 33475, 33476, 33478, 33496, 33500, 33501, 33502, 33503, 33504, 33505, 33506, 33507, 33510, 33511, 33512, 33513, 33514, 33516, 33533, 33534, 33535, 33536, 33542, 33545, 33548, 33600, 33602, 33606, 33608, 33610, 33611, 33612, 33615, 33617, 33619, 33620, 33621, 33622, 33641, 33645, 33647, 33660, 33665, 33670, 33675, 33676, 33677, 33681, 33684, 33688, 33690, 33692, 33694, 33697, 33702, 33710, 33720, 33722, 33724, 33726, 33730, 33732, 33735, 33736, 33737, 33750, 33755, 33762, 33764, 33766, 33767, 33770, 33771, 33774, 33775, 33776, 33777, 33778, 33779, 33780, 33781, 33782, 33783, 33786, 33788, 33800, 33802, 33803, 33813, 33814, 33820, 33822, 33824, 33840, 33845, 33851, 33852, 33853, 33858, 33859, 33863, 33864, 33871, 33875, 33877, 33880, 33881, 33883, 33886, 33910, 33915, 33916, 33917, 33920, 33922, 33925, 33926, 33935, 33945, 33971, 33974, 34001, 34051, 34101, 34111, 34151, 34201, 34203, 34401, 34421, 34451, 34471, 34490, 34501, 34502, 34510, 34520, 34530, 34701, 34702, 34703, 34704, 34705, 34706, 34707, 34708, 34710, 34712, 34718, 34830, 34831, 34832, 35001, 35002, 35005, 35011, 35013, 35021, 35022, 35045, 35081, 35082, 35091, 35092, 35102, 35103, 35111, 35112, 35121, 35122, 35131, 35132, 35141, 35142, 35151, 35152, 35180, 35182, 35184, 35188, 35189, 35190, 35201, 35206, 35207, 35211, 35216, 35221, 35226, 35231, 35236, 35241, 35246, 35251, 35256, 35261, 35266, 35271, 35276, 35281, 35286, 35301, 35302, 35303, 35304, 35305, 35311, 35321, 35331, 35341, 35351, 35355, 35361, 35363, 35371, 35372, 35501, 35506, 35508, 35509, 35510, 35511, 35512, 35515, 35516, 35518, 35521, 35522, 35523, 35525, 35526, 35531, 35533, 35535, 35536, 35537, 35538, 35539, 35540, 35556, 35558, 35560, 35563, 35565, 35566, 35570, 35571, 35583, 35585, 35587, 35601, 35606, 35612, 35616, 35621, 35623, 35626, 35631, 35632, 35633, 35634, 35636, 35637, 35638, 35642, 35645, 35646, 35647, 35650, 35654, 35656, 35661, 35663, 35665, 35666, 35671, 35691, 35693, 35694, 35695, 35701, 35702, 35703, 35800, 35820, 35840, 35860, 35870, 35875, 35876, 35879, 35881, 35883, 35884, 35901, 35903, 35905, 35907, 36260, 36261, 36262, 36818, 36819, 36820, 36821, 36823, 36825, 36830, 36831, 36832, 36833, 36835, 36838, 37140, 37145, 37160, 37180, 37181, 37215, 37216, 37217, 37218, 37500, 37565, 37600, 37605, 37606, 37607, 37615, 37616, 37617, 37618, 37619, 37650, 37660, 37700, 37718, 37722, 37735, 37760, 37761, 37780, 37785, 37788, 37790, 38100, 38101, 38115, 38120, 38305, 38308, 38380, 38381, 38382, 38520, 38525, 38530, 38531, 38542, 38550, 38555, 38562, 38564, 38700, 38720, 38724, 38740, 38745, 38760, 38765, 38770, 38780, 38794, 39000, 39010, 39200, 39220, 39501, 39503, 39540, 39541, 39545, 39560, 39561, 40500, 40510, 40520, 40525, 40527, 40530, 40650, 40652, 40654, 40700, 40701, 40702, 40720, 40761, 40814, 40816, 40818, 40819, 40840, 40842, 40843, 40844, 40845, 41006, 41007, 41008, 41009, 41015, 41016, 41017, 41018, 41112, 41113, 41114, 41116, 41120, 41130, 41135, 41140, 41145, 41150, 41153, 41155, 41510, 41512, 41520,

© 2023 Wang Y et al. *JAMA Network Open.*

		41823, 41827, 41872, 41874, 42107, 42120, 42140, 42145, 42200, 42205, 42210, 42215, 42220, 42225, 42226, 42227, 42235, 42260, 42305, 42335, 42340, 42408, 42409, 42410, 42415, 42420, 42425, 42426, 42440, 42450, 42500, 42505, 42507, 42509, 42510, 42600, 42665, 42725, 42810, 42815, 42820, 42821, 42825, 42826, 42830, 42831, 42835, 42836, 42842, 42844, 42845, 42860, 42870, 42890, 42892, 42894, 42950, 42953, 42955, 42961, 42962, 42970, 42971, 42972, 43020, 43030, 43045, 43100, 43101, 43107, 43108, 43112, 43113, 43116, 43117, 43118, 43121, 43122, 43123, 43124, 43130, 43135, 43180, 43279, 43280, 43281, 43282, 43284, 43285, 43286, 43287, 43288, 43300, 43305, 43310, 43312, 43313, 43314, 43320, 43325, 43327, 43328, 43330, 43331, 43332, 43333, 43334, 43335, 43336, 43337, 43340, 43341, 43351, 43352, 43360, 43361, 43400, 43405, 43410, 43415, 43420, 43425, 43496, 43500, 43501, 43502, 43510, 43520, 43605, 43610, 43611, 43620, 43621, 43622, 43631, 43632, 43633, 43634, 43640, 43641, 43644, 43645, 43651, 43652, 43653, 43770, 43771, 43772, 43773, 43774, 43775, 43800, 43810, 43820, 43825, 43830, 43831, 43832, 43840, 43842, 43843, 43845, 43846, 43847, 43848, 43850, 43855, 43860, 43865, 43870, 43880, 43886, 43887, 43888, 44005, 44010, 44020, 44021, 44025, 44050, 44055, 44110, 44111, 44120, 44125, 44126, 44127, 44130, 44140, 44141, 44143, 44144, 44145, 44146, 44147, 44150, 44151, 44155, 44156, 44157, 44158, 44160, 44180, 44186, 44187, 44188, 44202, 44204, 44205, 44206, 44207, 44208, 44210, 44211, 44212, 44227, 44300, 44310, 44312, 44314, 44316, 44320, 44322, 44340, 44345, 44346, 44602, 44603, 44604, 44605, 44615, 44620, 44625, 44626, 44640, 44650, 44660, 44661, 44680, 44700, 44800, 44820, 44850, 44900, 44950, 44960, 44970, 45000, 45020, 45100, 45108, 45110, 45111, 45112, 45113, 45114, 45116, 45119, 45120, 45121, 45123, 45126, 45130, 45135, 45136, 45150, 45160, 45171, 45172, 45190, 45395, 45397, 45400, 45402, 45500, 45505, 45540, 45541, 45550, 45560, 45562, 45563, 45800, 45805, 45820, 45825, 46040, 46045, 46060, 46070, 46200, 46250, 46255, 46257, 46258, 46260, 46261, 46262, 46270, 46275, 46280, 46285, 46288, 46700, 46705, 46707, 46710, 46712, 46715, 46716, 46730, 46735, 46740, 46742, 46744, 46746, 46748, 46750, 46751, 46753, 46760, 46761, 46930, 46945, 46946, 46947, 46948, 47010, 47015, 47100, 47120, 47122, 47125, 47130, 47135, 47140, 47141, 47142, 47144, 47300, 47350, 47360, 47361, 47362, 47370, 47371, 47380, 47381, 47400, 47420, 47425, 47460, 47480, 47562, 47563, 47564, 47570, 47600, 47605, 47610, 47612, 47620, 47700, 47701, 47711, 47712, 47715, 47720, 47721, 47740, 47741, 47760, 47765, 47780, 47785, 47800, 47801, 47802, 47900, 48000, 48001, 48020, 48100, 48105, 48120, 48140, 48145, 48146, 48148, 48150, 48152, 48153, 48154, 48155, 48500, 48510, 48520, 48540, 48545, 48547, 48548, 48554, 48556, 49000, 49002, 49010, 49020, 49040, 49060, 49062, 49203, 49204, 49205, 49215, 49250, 49255, 49323, 49402, 49419, 49425, 49426, 49491, 49492, 49495, 49496, 49500, 49501, 49505, 49507, 49520, 49521, 49525, 49540, 49550, 49553, 49555, 49557, 49560, 49561, 49565, 49566, 49570, 49572, 49580, 49582, 49585, 49587, 49590, 49600, 49605, 49606, 49610, 49611, 49650, 49651, 49652, 49653, 49654, 49655, 49656, 49657, 49900, 49904, 49906, 50010, 50020, 50040, 50045,

© 2023 Wang Y et al. *JAMA Network Open.*

		50060, 50065, 50070, 50075, 50080, 50081, 50100, 50120, 50125, 50130, 50135, 50205, 50220, 50225, 50230, 50234, 50236, 50240, 50250, 50280, 50290, 50320, 50340, 50360, 50365, 50370, 50380, 50400, 50405, 50500, 50520, 50525, 50526, 50540, 50541, 50542, 50543, 50544, 50545, 50546, 50547, 50548, 50562, 50590, 50600, 50605, 50610, 50620, 50630, 50650, 50660, 50700, 50715, 50722, 50725, 50727, 50728, 50740, 50750, 50760, 50770, 50780, 50782, 50783, 50785, 50800, 50810, 50815, 50820, 50825, 50830, 50840, 50845, 50860, 50900, 50920, 50930, 50940, 50945, 50947, 50948, 51020, 51030, 51040, 51045, 51050, 51060, 51065, 51080, 51500, 51520, 51525, 51530, 51535, 51550, 51555, 51565, 51570, 51575, 51580, 51585, 51590, 51595, 51596, 51597, 51800, 51820, 51840, 51841, 51845, 51860, 51865, 51880, 51900, 51920, 51925, 51940, 51960, 51980, 51990, 51992, 52400, 52450, 52500, 52601, 52630, 52640, 52647, 52648, 52649, 52700, 53010, 53040, 53080, 53085, 53210, 53215, 53220, 53230, 53235, 53240, 53250, 53400, 53405, 53410, 53415, 53420, 53425, 53430, 53431, 53440, 53442, 53444, 53445, 53446, 53447, 53448, 53449, 53450, 53460, 53500, 53502, 53505, 53510, 53515, 53520, 53850, 53852, 53854, 53860, 54110, 54111, 54112, 54115, 54120, 54125, 54130, 54135, 54205, 54300, 54304, 54308, 54312, 54316, 54318, 54322, 54324, 54326, 54328, 54332, 54336, 54340, 54344, 54348, 54352, 54360, 54380, 54385, 54390, 54400, 54401, 54405, 54406, 54408, 54410, 54411, 54415, 54416, 54417, 54420, 54430, 54435, 54437, 54438, 54440, 54512, 54520, 54522, 54530, 54535, 54550, 54560, 54600, 54640, 54650, 54660, 54670, 54680, 54690, 54692, 54830, 54840, 54860, 54861, 54865, 54900, 54901, 55040, 55041, 55060, 55110, 55120, 55150, 55175, 55180, 55200, 55250, 55400, 55500, 55520, 55530, 55535, 55540, 55550, 55600, 55605, 55650, 55680, 55720, 55725, 55801, 55810, 55812, 55815, 55821, 55831, 55840, 55842, 55845, 55860, 55862, 55865, 55866, 55873, 55875, 55880, 56620, 56625, 56630, 56631, 56632, 56633, 56634, 56637, 56640, 56805, 57010, 57106, 57107, 57109, 57110, 57111, 57120, 57200, 57210, 57220, 57230, 57240, 57250, 57260, 57265, 57268, 57270, 57280, 57282, 57283, 57284, 57285, 57287, 57288, 57289, 57291, 57292, 57295, 57296, 57300, 57305, 57307, 57308, 57310, 57311, 57320, 57330, 57335, 57423, 57425, 57426, 57520, 57522, 57530, 57531, 57540, 57545, 57550, 57555, 57556, 57700, 57720, 58140, 58145, 58146, 58150, 58152, 58180, 58200, 58210, 58240, 58260, 58262, 58263, 58267, 58270, 58275, 58280, 58285, 58290, 58291, 58292, 58294, 58346, 58400, 58410, 58520, 58540, 58541, 58542, 58543, 58544, 58545, 58546, 58548, 58550, 58552, 58553, 58554, 58565, 58570, 58571, 58572, 58573, 58575, 58600, 58605, 58660, 58662, 58670, 58671, 58672, 58673, 58674, 58700, 58720, 58740, 58750, 58752, 58760, 58770, 58800, 58805, 58820, 58822, 58825, 58900, 58920, 58925, 58940, 58943, 58950, 58951, 58952, 58953, 58954, 58956, 58957, 58958, 58960, 59100, 59120, 59121, 59130, 59135, 59136, 59140, 59150, 59151, 59812, 59820, 59821, 59830, 59850, 59851, 59852, 59855, 59856, 59857, 59870, 60200, 60210, 60212, 60220, 60225, 60240, 60252, 60254, 60260, 60270, 60271, 60280, 60281, 60500, 60502, 60505, 60520, 60521, 60522, 60540, 60545, 60600, 60605, 60650, 61105, 61108, 61120, 61140, 61150, 61151, 61154, 61156,

© 2023 Wang Y et al. *JAMA Network Open.*

		61215, 61250, 61253, 61304, 61305, 61312, 61313, 61314, 61315, 61320, 61321, 61322, 61323, 61330, 61333, 61340, 61343, 61345, 61450, 61458, 61460, 61500, 61501, 61510, 61512, 61514, 61516, 61518, 61519, 61520, 61521, 61522, 61524, 61526, 61530, 61531, 61533, 61534, 61535, 61536, 61537, 61538, 61539, 61540, 61541, 61543, 61544, 61545, 61546, 61548, 61550, 61552, 61556, 61557, 61558, 61559, 61563, 61564, 61566, 61567, 61570, 61571, 61575, 61576, 61580, 61581, 61582, 61583, 61584, 61585, 61586, 61590, 61591, 61592, 61595, 61596, 61597, 61598, 61600, 61601, 61605, 61606, 61607, 61608, 61613, 61615, 61616, 61618, 61619, 61680, 61682, 61684, 61686, 61690, 61692, 61697, 61698, 61700, 61702, 61703, 61705, 61708, 61710, 61711, 61720, 61735, 61750, 61751, 61760, 61770, 61790, 61791, 61796, 61798, 61850, 61860, 61863, 61867, 61880, 61885, 61886, 62000, 62005, 62010, 62100, 62115, 62117, 62120, 62121, 62140, 62141, 62142, 62143, 62145, 62146, 62147, 62161, 62162, 62164, 62165, 62180, 62190, 62192, 62200, 62201, 62220, 62223, 62225, 62230, 62256, 62258, 62287, 62292, 62294, 62351, 62380, 63001, 63003, 63005, 63011, 63012, 63015, 63016, 63017, 63020, 63030, 63040, 63042, 63045, 63046, 63047, 63050, 63051, 63055, 63056, 63064, 63075, 63077, 63081, 63085, 63087, 63090, 63101, 63102, 63170, 63172, 63173, 63185, 63190, 63191, 63194, 63195, 63196, 63197, 63198, 63199, 63200, 63250, 63251, 63252, 63265, 63266, 63267, 63268, 63270, 63271, 63272, 63273, 63275, 63276, 63277, 63278, 63280, 63281, 63282, 63283, 63285, 63286, 63287, 63290, 63300, 63301, 63302, 63303, 63304, 63305, 63306, 63307, 63600, 63620, 63655, 63662, 63664, 63700, 63702, 63704, 63706, 63707, 63709, 63710, 63740, 63741, 63744, 63746, 64568, 64569, 64570, 64575, 64580, 64581, 64702, 64704, 64708, 64712, 64713, 64714, 64716, 64718, 64719, 64721, 64722, 64726, 64732, 64734, 64736, 64738, 64740, 64742, 64744, 64746, 64755, 64760, 64763, 64766, 64771, 64772, 64774, 64776, 64782, 64784, 64786, 64788, 64790, 64792, 64802, 64804, 64809, 64818, 64820, 64821, 64822, 64823, 64831, 64834, 64835, 64836, 64840, 64856, 64857, 64858, 64861, 64862, 64864, 64865, 64866, 64868, 64885, 64886, 64890, 64891, 64892, 64893, 64895, 64896, 64897, 64898, 64905, 64907, 64910, 64911, 64912, 65091, 65093, 65101, 65103, 65105, 65110, 65112, 65114, 65125, 65130, 65135, 65140, 65150, 65155, 65175, 65235, 65260, 65265, 65272, 65273, 65275, 65280, 65285, 65286, 65290, 65400, 65420, 65426, 65436, 65450, 65600, 65710, 65730, 65750, 65755, 65756, 65770, 65772, 65775, 65780, 65781, 65782, 65785, 65810, 65815, 65820, 65850, 65860, 65865, 65870, 65875, 65880, 65900, 65920, 65930, 66130, 66150, 66155, 66160, 66170, 66172, 66174, 66175, 66179, 66180, 66183, 66184, 66185, 66225, 66250, 66500, 66505, 66600, 66605, 66625, 66630, 66635, 66680, 66682, 66700, 66710, 66711, 66720, 66740, 66762, 66770, 66820, 66821, 66825, 66830, 66840, 66850, 66852, 66920, 66930, 66940, 66982, 66983, 66984, 66985, 66986, 66987, 66988, 67005, 67010, 67015, 67025, 67027, 67030, 67031, 67036, 67039, 67040, 67041, 67042, 67043, 67107, 67108, 67110, 67113, 67115, 67120, 67121, 67141, 67145, 67208, 67210, 67218, 67220, 67229, 67250, 67255, 67311, 67312, 67314, 67316, 67318, 67343, 67400, 67405, 67412, 67413, 67414, 67420, 67430, 67440, 67445,

© 2023 Wang Y et al. *JAMA Network Open.*

		67450, 67550, 67560, 67570, 67808, 67835, 67880, 67882, 67900, 67901, 67902, 67903, 67904, 67906, 67908, 67909, 67911, 67912, 67914, 67915, 67916, 67917, 67921, 67922, 67923, 67924, 67935, 67950, 67961, 67966, 67971, 67973, 67974, 67975, 68130, 68320, 68325, 68326, 68328, 68330, 68335, 68340, 68360, 68362, 68500, 68505, 68520, 68540, 68550, 68700, 68720, 68745, 68750, 68770, 69110, 69120, 69140, 69145, 69150, 69155, 69310, 69320, 69440, 69450, 69501, 69502, 69505, 69511, 69530, 69535, 69550, 69552, 69554, 69601, 69602, 69603, 69604, 69620, 69631, 69632, 69633, 69635, 69636, 69637, 69641, 69642, 69643, 69644, 69645, 69646, 69650, 69660, 69661, 69662, 69666, 69667, 69670, 69676, 69700, 69711, 69714, 69715, 69717, 69718, 69720, 69725, 69740, 69745, 69805, 69806, 69905, 69910, 69915, 69930, 69950, 69955, 69960, 69970, 77750, 77761, 77762, 77763, 92986, 92987, 92990

Note: HCPCS, Healthcare Common Procedure Coding System

© 2023 Wang Y et al. *JAMA Network Open.*

eTable 4. Codes for Bleeding Events

Conditions	ICD-10-CM	ICD-9-CM
**Major bleeding**		
Non-traumatic intracranial bleed	I60, I61, I62, S064, S065	430, 431, 4320, 4329, 4321, 4389, 4380, 43811, 43812, 48313, 43814, 43819, 43831, 43832, 43830, 43841, 43842, 43840, 43821, 43822, 43820, 43851, 43852, 43850, 43853, 43881, 43882, 43883, 43884, 43889, 4389
Sequelae of nontraumatic intracranial bleed	I690, I691, I692	85241, 9070, 85242, 85243, 85244, 85245, 85246, 85221, 85222, 85223, 85224, 85225, 85226, 85201, 85202, 85203, 85204, 85205, 85206, 85301, 85302, 85303, 85304, 85305, 85306, 85142, 85162, 85143, 85163, 85144, 85164, 85145, 85165, 85146, 85166
Upper GI bleeding	I85, I864, K920, K3182, K250, K252, K254, K256, K260, K262, K264, K266, K270, K272, K274, K276, K280, K282, K284, K286, K2901, K2921, K2931, K2941, K2951, K2961, K2971, K2981, K2991, K2081, K209, K2101, K2211, K226, I983, I942, K922	4560, 4561, 4562, 4568, 5780, 53784, 5310, 5312, 5314, 5316, 5320, 5322, 5324, 5326, 5330, 5332, 5334, 5336, 5340, 5342, 5344, 5346, 53501, 53531, 53511, 53541, 53521, 53551, 53561, 53021, 5307, 53082, 53783, 5789
Lower GI bleeding	K921, K5521, K50011, K50111, K50811, K50911, K51011, K51211, K51311, K51411, K51511, K51811, K51911, K5701, K5711, K5713, K5731, K5733, K5741, K5751, K5753, K5781, K5791, K5793, K6381, K625, K661, K922<br>K625	5781, 56985, 5550, 5551, 5552, 5559, 5566, 5562, 5563, 5564, 5565, 5568, 5569, 56203, 5695, 56202, 56212, 56213, 56986, 5693, 4552, 4555, 4558, 5780, 5789
Coagulation defect or other hemorrhagic disorders	D689	2867, 28652, 28653, 2869, 23871
Genitourinary tract	N02, R31, N421	5812, 5813, 5811, 58189, 5819, 59971, 59972, 59970, 6021
Hemorrhage from respiratory passages	R04	7848, 7847, 78630, 78631, 78639
Hemorrhage in the eye/orbit	H356, H4702, H3130, H3131, H3141, H113, H0532, H431, H210, H450	36281, 37742, 36361, 36362, 36372, 37272, 37643, 37923

© 2023 Wang Y et al. *JAMA Network Open.*

Liver hemorrhage	K762	570
Gynecological (not pregnancy related)	N92, N930, N938, N939, N950	6264, 6269, 6262, 6266, 6263, 6265, 6270, 6268, 6267, 6271, 6272, 6273, 6278, 6279, 6238
Gastric, duodenal, peptic ulcer with hemorrhage	K250, K252, K254, K256, K260, K262, K264, K266, K270, K272, K274, K276, K280, K282, K284, K286, K290	5316, 5320, 5322, 5324, 5326, 5330, 5332, 5334, 5336
Hemoperitoneum	K661	56881
**Non-major bleeding**		
Otorrhagia	H922	No corresponding icd-9 codes
Hemopericardium	I230, I312, S260	4230
Bleeding due to anticoagulation	D683	28659
Acute posthemorrhagic anemia	D62	2851
Hemothorax	J942	51189
Hemorrhage into the bladder wall.	N3289	5967
Hemarthrosis	M250	7191
Puncture site bleeding due to PCI	T85838, T82838	No corresponding icd-9 codes
Gingival bleeding	K068	5238
Other hemorrhages unspecified	D699, D473, R58, D698	4590

© 2023 Wang Y et al. *JAMA Network Open.*

eTable 5. Association Between Patient Characteristics at Baseline and Bleeding Risk and the Initial Prescription for Prasugrel vs Ticagrelor Among Patients With ACS Undergoing PCI

		Prasugrel (n=3421) N (%)	Ticagrelor (n=11818) N (%)	aOR (95% CI) model 1[a]	aOR (95% CI) model 2[a]
**Age, mean (SD), year**		53.93 (7.26)	54.40 (7.13)	1.00 (0.99-1.00)	1.00 (0.99-1.00)
**Female**		671 (19.61)	2623 (22.19)	1.20 (1.09-1.32)[s]	1.18 (1.08-1.30)[s]
**ACS type**	**NSTEMI**	1130 (33.03)	3782 (32.00)	Ref	Ref
	**STEMI**	1832 (53.55)	6263 (53.00)	0.98 (0.90-1.07)	0.98 (0.90-1.07)
	**Other MI**	459 (13.42)	1773 (15.00)	0.87 (0.77-0.98)	0.87 (0.77-0.98)
**Copay of the $P2Y_{12}$ inhibitor, median (IQR), USD**		0 (0-25)	0 (0-25)	0.99 (0.99-1.00)	1.00 (1.00-1.00)
**Health plan type**	**PPO**	2101 (61.41)	6886 (58.27)	Ref	Ref
	**Comprehensive**	145 (4.24)	346 (2.93)	1.86 (1.51-2.24)[s]	-
	**HMO**	472 (13.80)	1552 (13.13)	1.02 (0.91-1.14)	-
	**Others**	703 (20.55)	3034 (25.67)	0.78 (0.71-0.86)[s]	-
**Region**	**Northeast**	432 (12.63)	2067 (17.57)	Ref	-
	**Northcentral**	650 (19.00)	2782 (23.54)	1.10 (0.96-1.26)	-
	**South**	1935 (56.56)	5612 (47.49)	1.64 (1.45-1.84)[s]	-
	**West**	388 (11.34)	1291 (10.92)	1.46 (1.25-1.67)[s]	-
**Hypertension**		8311 (70.32)	2386 (69.75)	0.95 (0.87-1.03)	-
**Diabetes**		1200 (35.08)	4235 (35.84)	0.98 (0.89-1.03)	-
**Dyslipidemia**		2523 (73.75)	8580 (72.60)	1.06 (0.97-1.16)	-
**Heart failure**		337 (9.85)	1293 (10.94)	0.92 (0.81-1.05)	-
**Peripheral vascular disease**		100 (2.92)	441 (3.73)	0.78 (0.63-0.98)[s]	-
**Arterial fibrillation**		118 (3.45)	467 (3.95)	0.87 (0.71-1.08)	-
**ARC-HBR composite major bleeding risk**		1172 (34.26)	4184 (35.40)	0.98 (0.90-1.07)	-
**ARC-HBR individual major bleeding risk item**[b]	**Recent major surgery**	49 (1.43)	231 (1.95)	-	0.82 (0.54-1.24)
	**Liver cirrhosis**	28 (0.82)	125 (1.06)	-	0.85 (0.61-1.20)
	**Anemia**	154 (4.50)	677 (5.73)	-	0.81 (0.67-0.98)[s]
	**Severe chronic kidney disease**	765 (22.36)	2752 (23.29)	-	0.98 (0.88-1.08)
	**Recent major bleeding**	204 (5.96)	685 (5.80)	-	1.14 (0.97-1.34)
	**Arteriovenous malformation**	6 (0.18)	31 (0.26)	-	0.87 (0.33-2.23)
	**Stroke**	87 (2.54)	420 (3.47)	-	0.79 (0.62-1.00)[s]
	**Cancer**	114 (3.33)	466 (3.94)	-	0.89 (0.72-1.10)
	**Thrombocytopenia**	39 (1.14)	168 (1.40)	-	0.88 (0.61-1.26)
	**Anti-coagulation use**	43 (1.26)	167 (1.41)	-	1.00 (0.70-1.41)

© 2023 Wang Y et al. *JAMA Network Open.*

Abbreviations

ACS, Acute Coronary Syndrome; NSTEMI, non-ST-elevation myocardial infarction; STEMI, ST-elevation myocardial infarction; PCI, Percutaneous Coronary Intervention; SD, Standard Deviation; IQR: Interquartile Range; aHR, adjusted Hazard Ratio; CI, Confidence Interval; HMO: Health Maintenance Organization; PPO: Preferred Provider Organization; ARC-HBR: Academic Research Consortium for High Bleeding Risk

[a] Model 1 only included ARC-HBR composite bleeding risk (binary) and model 2 included individual ARC-HBR major bleeding risk items.

[b] The ARC-HBR criteria definition and measurement window are defined in **Supplementary Table 2**

[s] Significant statistical results, two-side test (P<0.05)

© 2023 Wang Y et al. *JAMA Network Open.*

eTable 6. Association Between Patient Characteristics at Baseline and Bleeding Risk and Deescalation to Clopidogrel Among Patients Started on Prasugrel or Ticagrelor

		Prasugrel model 1[a]	Prasugrel model 2[a]	Ticagrelor model 1[a]	Ticagrelor model 2[a]
		**aHR (95% CI)**	**aHR (95% CI)**	**aHR (95% CI)**	**aHR (95% CI)**
**Age, year**		1.00 (0.99-1.02)	1.00 (0.99-1.02)	1.02 (1.01-1.02)[s]	1.01 (1.01-1.02)[s]
**Female**		0.92 (0.72-1.19)	0.94 (0.72-1.21)	0.75 (0.67-0.84)[s]	0.76 (0.67-0.85)[s]
**ACS type**	**NSTEMI**	Ref	Ref	Ref	Ref
	**STEMI**	1.14 (0.90-1.44)	1.13 (0.89-1.42)	0.93 (0.83-1.04)	0.94 (0.84-1.05)
	**Other MI**	1.05 (0.74-1.50)	1.05 (0.74-1.49)	0.96 (0.82-1.13)	0.97 (0.82-1.14)
**Copay of the $P2Y_{12}$ inhibitor, USD**		1.00 (1.00-1.00)	1.00 (1.00-1.00)	1.00 (1.00-1.00)	1.00 (1.00-1.00)
**Health plan type**	**PPO**	Ref	Ref	Ref	Ref
	**Comprehensive**	0.67 (0.37-1.22)	0.67 (0.37-1.22)	0.93 (0.68-1.26)	0.94 (0.69-1.27)
	**HMO**	1.04 (0.77-1.39)	1.05 (0.78-1.40)	1.09 (0.94-1.27)	1.09 (0.94-1.27)
	**Others**	0.88 (0.67-1.16)	0.89 (0.68-1.17)	0.99 (0.88-1.12)	1.00 (0.88-1.13)
**Region**	**Northeast**	Ref	Ref	Ref	Ref
	**Northcentral**	1.43 (0.98-2.07)	1.46 (1.00-2.12)[s]	1.30 (1.11-1.53)[s]	1.31 (1.12-1.54)[s]
	**South**	1.10 (0.78-1.54)	1.10 (0.79-1.55)	1.01 (0.87-1.18)	1.02 (0.88-1.19)
	**West**	1.46 (0.97-2.19)	1.45 (0.96-2.19)	1.46 (1.21-1.76)[s]	1.48 (1.23-1.79)[s]
**Hypertension**		0.99 (0.79-1.24)	0.99 (0.78-1.25)	1.04 (0.92-1.18)	1.04 (0.92-1.16)
**Diabetes**		0.90 (0.71-1.14)	0.96 (0.74-1.23)	0.99 (0.89-1.11)	0.99 (0.89-1.13)
**Dyslipidemia**		0.82 (0.65-1.03)	0.82 (0.65-1.04)	0.86 (0.77-0.96)[s]	0.86 (0.77-0.97)[s]
**Heart failure**		1.10 (0.79-1.53)	1.11 (0.79-1.55)	1.11 (0.95-1.30)	1.10 (0.94-1.28)
**Peripheral vascular disease**		1.20 (0.67-2.16)	1.16 (0.64-2.10)	1.12 (0.87-1.45)	1.11 (0.86-1.44)
**Arterial fibrillation**		0.80 (0.44-1.47)	0.69 (0.36-1.30)	1.11 (0.86-1.43)	1.01 (0.78-1.31)
**ARC-HBR composite major bleeding risk**		1.38 (1.10-1.74)[s]	-	1.06 (0.94-1.18)	-
**ARC-HBR individual major bleeding risk item**[b]	**Recent major surgery**	-	2.23 (0.98-5.09)	-	0.93 (0.57-1.50)
	**Liver cirrhosis**	-	0.87 (0.32-2.38)	-	0.89 (0.59-1.35)
	**Anemia**	-	0.92 (0.55-1.54)	-	1.30 (1.05-1.60)[s]
	**Severe chronic kidney disease**	-	1.13 (0.85-1.50)	-	1.00 (0.87-1.14)
	**Recent major bleeding**	-	1.20 (0.78-1.83)	-	0.89 (0.71-1.12)
	**Arteriovenous malformation**	-	2.12 (0.23-19.53)	-	1.91 (0.77-4.75)
	**Stroke**	-	1.58 (0.90-2.78)	-	1.08 (0.83-1.42)
	**Cancer**	-	1.15 (0.64-2.09)	-	1.21 (0.95-1.54)
	**Thrombocytopenia**	-	0.91 (0.33-2.53)	-	0.85 (0.56-1.31)
	**Anti-coagulation use**	-	2.20 (1.19-4.05)[s]	-	1.72 (1.28-2.31)[s]

© 2023 Wang Y et al. *JAMA Network Open.*

Abbreviations

ACS, Acute Coronary Syndrome; NSTEMI, non-ST-elevation myocardial infarction; STEMI, ST-elevation myocardial infarction; PCI, Percutaneous Coronary Intervention; aHR, adjusted Hazard Ratio; CI, Confidence Interval; HMO: Health Maintenance Organization; PPO: Preferred Provider Organization; ARC-HBR: Academic Research Consortium for High Bleeding Risk

[a] Model 1 only included ARC-HBR composite bleeding risk (binary) and model 2 included individual ARC-HBR major bleeding risk items.

[b] The ARC-HBR criteria definition and measurement window are defined in **Supplementary Table 2**

[s] Significant statistical results, two-side test (P<0.05)

© 2023 Wang Y et al. *JAMA Network Open.*
