# Supplementary material for: Assessing the Clinical Treatment Dynamics of Antiplatelet Therapy Following Acute Coronary Syndrome and Percutaneous Coronary Intervention in the US
Source: JAMA Netw Open. 2023 Apr 17;6(4):e238585. doi: 10.1001/jamanetworkopen.2023.8585 (PMC10111179; doi:10.1001/jamanetworkopen.2023.8585)
Supplement: Supplement 2. — Data Sharing Statement [file jamanetwopen-e238585-s002.pdf]

Data Sharing Statement

Wang. Assessing the Clinical Treatment Dynamics of Antiplatelet Therapy Following Acute Coronary Syndrome and Percutaneous Coronary Intervention in the US. *JAMA Netw Open*. Published April 17, 2023. doi:10.1001/jamanetworkopen.2023.8585

Data
Data available: No

Additional Information
Explanation for why data not available: The claims data contains personal identifying information and according to the data privacy agreement are not to be shared with others unless there is approval from the IRB.
